# Impact of Intrinsic Density Functional Theory Errors on the Predictive Power of Nitrogen Cycle Electrocatalysis Models

**DOI:** 10.1021/acscatal.1c05333

**Published:** 2022-04-06

**Authors:** Ricardo Urrego-Ortiz, Santiago Builes, Federico Calle-Vallejo

**Affiliations:** † Departamento de Ingeniería de Procesos, Universidad EAFIT, Carrera 49 No 7 sur 50, 050022 Medellín, Colombia; ‡ Department of Materials Science and Chemical Physics & Institute of Theoretical and Computational Chemistry (IQTCUB), University of Barcelona, C/Martí i Franquès 1, 08028 Barcelona, Spain

Oxidized nitrogen species can pollute both the atmosphere^1−3^ and water bodies.^4−6^ Their concentrations are worryingly increasing because of anthropogenic activities such as the combustion of fossil fuels and intensive agriculture.^7−11^ An alternative to remediate their negative impact is to reduce them into unharmful molecular nitrogen (N_2_) or valuable ammonia (NH_3_),^12−14^ thereby dynamizing the nitrogen cycle. In principle, electrocatalysis could be used as a green technology for these processes if the necessary energy input comes from renewable sources.^12,15^ However, the design of active, selective, and stable catalysts for the reduction of nitrogen oxides is not trivial. In that regard, density functional theory (DFT) calculations could serve as a supplement, support, or guide to experiments.^16−23^

DFT is widely used in computational chemistry for the modeling of solids. Specifically, exchange-correlation functionals at the generalized gradient approximation (GGA) have shown high accuracy with low computational requirements when predicting the ground-state properties of bulk and surface metals.^24,25^ However, when predicting gas-phase energetics, the limitations of GGA functionals are well-known (e.g., overbinding energy of N_2_ and O_2_)^25−27^ and predictions in line with experiments are only expected on the basis of error cancellation, i.e., when similar compounds appear in opposite sides of chemical reactions.^28−30^ The inaccuracies may be reduced by the use of meta-GGA functionals, which represent a step up in the hierarchy of exchange-correlation approximations.^31^ Because functionals at the meta-GGA level take into account the kinetic energy density of the Kohn–Sham orbitals, they are supposedly better than GGAs for molecules, while metals are still accurately described.^32,33^

Gas-phase errors are problematic in heterogeneous catalysis, where an accurate description of the gas phase is paramount for adsorption and desorption steps. Such steps happen each at least once in every catalytic reaction. In spite of their gas-phase errors, GGA functionals are extensively used in catalysis given their low computational requirements. Previous efforts have been devoted to (i) benchmarking their performance for predicting the enthalpies and entropies of adsorption of various systems^34−37^ and (ii) combining different functionals to boost their accuracy.^38,39^ Considering recent error analysis on nitrogen-containing organic compounds,^40^ if DFT at the GGA level is used to model reactions involving nitrogen oxides, it is expected that the calculated energies will entail large errors, in particular for highly oxidized species, such as nitrate and nitrite. Thus, accurately assessing the energetics of reactions such as nitrate reduction or electrochemical nitrogen oxidation remains challenging.

Herein, we show that large errors are encountered in the GGA and meta-GGA formation enthalpies of 11 oxidized nitrogen species in the gas phase. Importantly, the errors scale with the number of oxygens in the structure and the scaling factor is approximately constant for all the functionals studied. This exposes an intrinsic GGA and meta-GGA limitation that must be overcome if accurate predictions are sought after for the modeling of catalytic redox processes among nitrogen-containing species. Furthermore, we show the effects of intrinsic gas-phase errors on adsorption-energy scaling relations and volcano plots for two electrocatalytic reactions and propose an inexpensive scheme to systematically correct such errors.

## Computational Details

**Figure 1:**
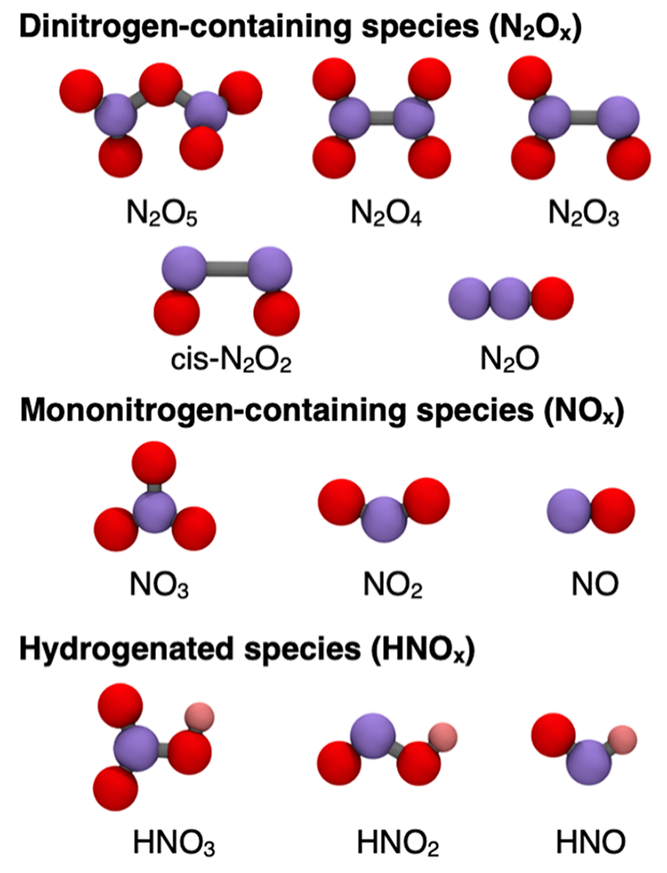
Schematics of the nitrogen species in this work. Purple, red, and pink spheres represent nitrogen, oxygen, and hydrogen atoms, respectively. The skeletal formulas of these molecules, where single and multiple bonds are depicted, can be found in Figure S2.

All the energies were calculated with DFT using the Vienna ab initio simulation package (VASP).^41^ The gas-phase calculations were performed for seven exchange-correlation functionals: four GGAs (PBE,^42^ PW91,^43^ RPBE,^44^ and BEEF-vdW^45^), one meta-GGA (TPSS^33^), and two hybrids (PBE0^46^ and B3LYP^47^). The adsorption energies on porphyrins were calculated with PBE and RPBE. Molecular representations of the nitrogen-containing compounds studied here are shown in Figure 1. Besides, Figure S2 provides the skeletal formulas of the oxidized nitrogen species, in which their single and multiple bonds are apparent.

For metalloporphyrins (see the schematic in Figure S3), spin-unrestricted calculations with and without adsorbates were performed, and the most stable spin state was selected in each case to assess the adsorption energies (Table S4). The computational hydrogen electrode was used to describe the energetics of proton–electron transfers.^48^ Further computational details, including the assessment of the free energies of adsorption and a comparison between experimental and computational zero point energies (ZPEs), are provided in section S1 of the Supporting Information. As the experimental and calculated ZPEs are nearly identical, we conclude that the discrepancies in the formation energies stem mainly from the total energies calculated with DFT.

## Detection and Correction of the Gas-Phase Errors

The formation energies of nitrogen compounds from their elements in their respective standard states can be calculated from eq 1.
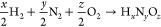
1where H_*x*_N_*y*_O_*z*_ is an oxidized nitrogen compound. We note that when the compounds do not contain hydrogen (i.e., N_2_O_*x*_ and NO_*x*_; see Figure 1), *x* = 0 in eq 1.

The total errors in the description of the oxidized nitrogen compounds (ε_H_*x*_N_*y*_O_*z*__^*T*^) are determined as the difference between the DFT-calculated and experimental enthalpies of formation (Δ_*f*_*H*_H_*x*_N_*y*_O_*z*__^DFT^ and Δ_*f*_*H*_H_*x*_N_*y*_O_*z*__^exp^), as in eq 2.
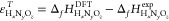
2

Experimental values were taken from thermodynamic tables.^49,50^ The total error of H_*x*_N_*y*_O_*z*_ encompasses the errors of the reactants and products of eq 1. Thus, the total error can be estimated from these individual errors as^40^
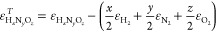
3where ε_H_2__, ε_N_2__, and ε_O_2__ are the errors of the reactants in eq 1 (H_2_, N_2_, and O_2_) and ε_H_*x*_N_*y*_O_*z*__ is the gas-phase error of the oxidized nitrogen compound itself, namely, the product of eq 1. Since H_2_ is generally well described by DFT, ε_H_2__ ≈ 0. Conversely, the triplet state of O_2_ is poorly described by GGA functionals,^25^ such that ε_O_2__ is typically large.^51^ O_2_ can be swiftly corrected using a semiempirical approach based on the formation energy of H_2_O.^28,40,48^ In addition, ε_N_2__ is usually substantial and can be calculated from the ammonia synthesis reaction, as explained elsewhere^40^ and in section S4.

If only the errors in O_2_ are corrected, the convoluted error of a specific nitrogen compound and that of N_2_ () can be calculated by combining eqs 2 and 3:
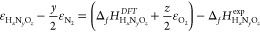
4

Furthermore, if the errors in O_2_ and N_2_ are simultaneously corrected, ε_H_*x*_N_*y*_O_*z*__ is found to be
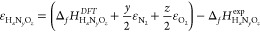
5

**Figure 2:**
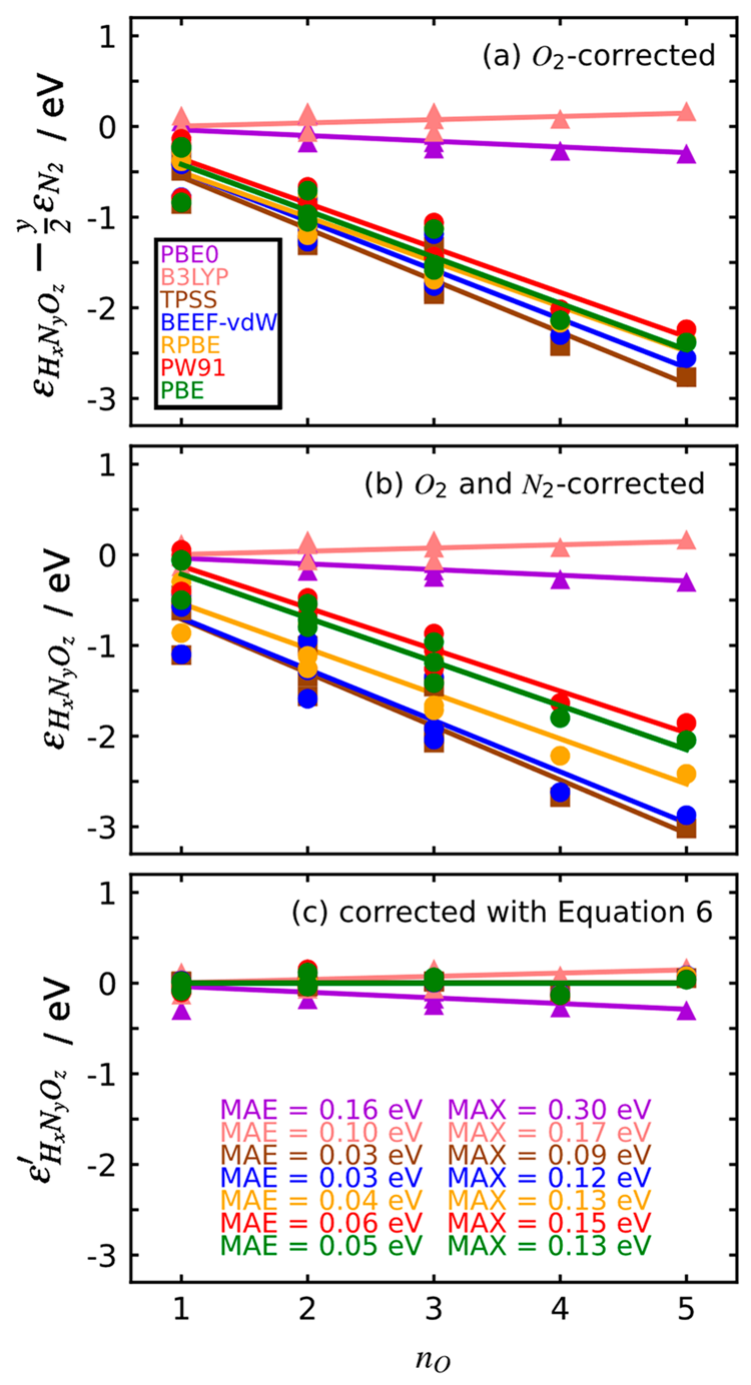
DFT errors in the formation enthalpies of nitrogen compounds as a function of their number of oxygen atoms (*n*_O_). Circles (●) are the calculated data points for the GGAs, squares (■) for TPSS, and triangles (▲) for hybrids. Least-squares linear fits are shown as continuous lines. (a) Errors obtained with the DFT calculations and the corrected energies of O_2_ using eq 4 (Table S2). (b) Errors obtained after correcting O_2_ and N_2_, which correspond to the isolated errors of each nitrogen-containing molecule in eq 5 (Table S3). (c) Residual errors (eq 7) after correcting GGA and meta-GGA functionals using eq 6 (Table S11). In all panels the hybrids are used as a benchmark and were not corrected at all (Table S2). The MAEs and MAX for panels a and b are given in section S2. For each value of *n*_O_, the species are as follows: 1, N_2_O, NO, HNO; 2, cis-N_2_O_2_, HNO_2_, NO_2_; 3, N_2_O_3_, HNO_3_, NO_3_; 4, N_2_O_4_; 5, N_2_O_5_.

Equations 4 and 5 can be used to progressively isolate the errors of all oxidized nitrogen compounds. Figure 2 shows these errors as functions of the number of oxygen atoms in the molecule (*n*_O_). Indeed, Figure 2a (O_2_ is corrected) and b (O_2_ and N_2_ are corrected) shows that such errors are large for GGA and meta-GGA functionals, with values as large as −3.0 eV. More importantly, the errors are linear functions of the number of oxygen atoms in the molecules and the linear trends have, on average, slopes of −0.5 eV/O atom for the GGA and meta-GGA functionals (see the specific values in Table S2). This implies the following:

(i) Progressively adding oxygen atoms to a nitrogen-containing molecule increases the magnitude of the DFT errors by roughly 0.5 eV each time, which is too large for accurate predictions of reaction energies and associated properties such as equilibrium potentials.
(ii) The errors are intrinsic; that is, they are due to the generalized gradient approximation. As such, they cannot be avoided by switching to other GGA or meta-GGA functionals. Conversely, the hybrid functionals PBE0 and B3LYP were not corrected at all because their trends in Figure 2 display nearly flat slopes of −0.06 and 0.04 eV/O atom, respectively. Besides, they have mean absolute errors (MAEs) of 0.16 and 0.10 eV. This is consistent with hybrid functionals being generally able to reproduce the experimental energetics of small molecules more closely than GGAs.^27,46,52,53^
(iii) DFT-based modeling of redox processes among nitrogen-containing compounds in Figure 1 entails sizable errors, in particular when there are large differences in the oxidation states of the reactants and products (e.g., nitrate reduction to N_2_).
(iv) Because the errors are systematic, a model can be made that simultaneously corrects all errors based on *n*_O_. One such method is detailed in the next paragraphs. We note that the dependence of the DFT errors on *n*_O_ can be rationalized by the presence of multiple (i.e., double or triple) bonds in H_*x*_N_*y*_O_*z*_, since it is known that DFT-GGAs often fail to accurately describe molecules with such bonds.^25,36^Figure S2 shows the skeletal structures of the molecules in Figure 1, in which single and multiple bonds are apparent. As all of them have unsaturated bonds and, in several cases, single and multiple bonds are intercalated, resonant structures are possible, which likely induce the large errors observed.^54^ This is in line with previous works showing that compounds comprising multiple bonds, such as nitrates or carboxylic acids, display large errors.^30,40^

When the linear trends in Figure 2a are used to correct the intrinsic errors in the formation enthalpies of the nitrogen compounds of the GGA and meta-GGA functionals, the averages of the MAEs and maximum absolute errors (MAX) are 0.18 and 0.40 eV (Table S12). Similarly, if the linear trends in Figure 2b are used to correct the calculated formation enthalpies, large errors are also obtained, with the averages of the MAEs and MAX being 0.18 and 0.38 eV (Table S13). The considerably lower errors in Figure 2c (average MAE of 0.04 eV and average MAX of 0.12 eV) are obtained by splitting the nitrogen-containing molecules into the three groups shown in Figure 1: (i) dinitrogen-containing species (N_2_O_*x*_), (ii) mononitrogen-containing species (NO_*x*_), and (iii) hydrogenated species (HNO_*x*_). We note that similar categories have previously been used to rationalize energetic and structural differences of oxidized nitrogen species.^55^ For each of these three groups, *n*_O_ is still linearly related to the errors and can be used to correct the DFT-calculated enthalpy (Figure S1). In this order of ideas, the corrected formation enthalpies are given by eq 6:
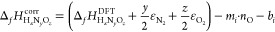
6where Δ_*f*_*H*_corr_ is the corrected enthalpy of the oxidized nitrogen species and *m*_*i*_ and *b*_*i*_ are, respectively, the slope and intercept of the regression line of group *i* = N_2_O_*x*_, NO_*x*_, and HNO_*x*_. The functional-dependent values of *m*_*i*_ and *b*_*i*_ are reported in Table 1. Regardless of the functional, the NO_*x*_ group has steeper slopes compared to the N_2_O_*x*_ and HNO_*x*_ groups. In fact, the average slopes for the three groups are −0.67 (NO_*x*_), −0.43 (N_2_O_*x*_), and −0.45 eV/O atom (HNO_*x*_). Further details of the fitting procedure appear in section S2. In analogy to eq 2, we calculate the residual errors (ε′) as
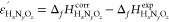
7

**Table 1:** Parameters to Correct the Formation Enthalpies of the Nitrogen-Containing Species for Each GGA and meta-GGA Functional Studied Using Eq 6^a^

Parameter	PBE	PW91	RPBE	BEEF-vdW	TPSS
*m*_N_2_O_*x*__	–0.42	–0.40	–0.41	–0.46	–0.49
*b*_N_2_O_*x*__	0.00	0.10	–0.46	–0.67	–0.61
*m*_HNO_*x*__	–0.45	–0.43	–0.42	–0.46	–0.50
*b*_HNO_*x*__	0.39	0.41	0.10	0.01	0.04
*m*_NO_*x*__	–0.67	–0.66	–0.65	–0.67	–0.68
*b*_NO_*x*__	0.59	0.69	0.23	0.09	0.05

a The slopes (*m*_*i*_) are in eV/O atom, and the intercepts (*b*_*i*_) are in eV.

We take N_2_O_5_ calculated with PBE to illustrate the use of the corrections from Table 1 and eq 6. In this case, the experimental value is Δ_*f*_*H*_N_2_O_5__^exp^ = 0.12 eV and the DFT-calculated formation enthalpy after the O_2_ correction is . This nitrogen oxide belongs to the group of dinitrogen oxides (N_2_O_*x*_); thus, *y*/2 = 1 and ε_N_2__^PBE^ = 0.34 eV.^40^ Hence, we have the following:. N_2_O_5_ belongs to the group of dinitrogen-containing species (N_2_O_*x*_) and contains five oxygen atoms; thus, *n*_O_ = 5. From Table 1 for N_2_O_*x*_, *m* = −0.42 eV/O and *b* = 0.00 *e*V. Hence, Δ_*f*_*H*_N_2_O_5__^corr, PBE^ = −1.93–5·(−0.42) – (0.00) = 0.16 eV, which deviates from experiments by 0.04 eV (ε_N_2_O_5__^′^ = 0.04 eV). Thus, after applying the corrections, the error changes from ε_N_2_O_5__^*T*^ = −2.38 eV to ε_N_2_O_5__^′^ = 0.04 eV.

**Figure 3:**
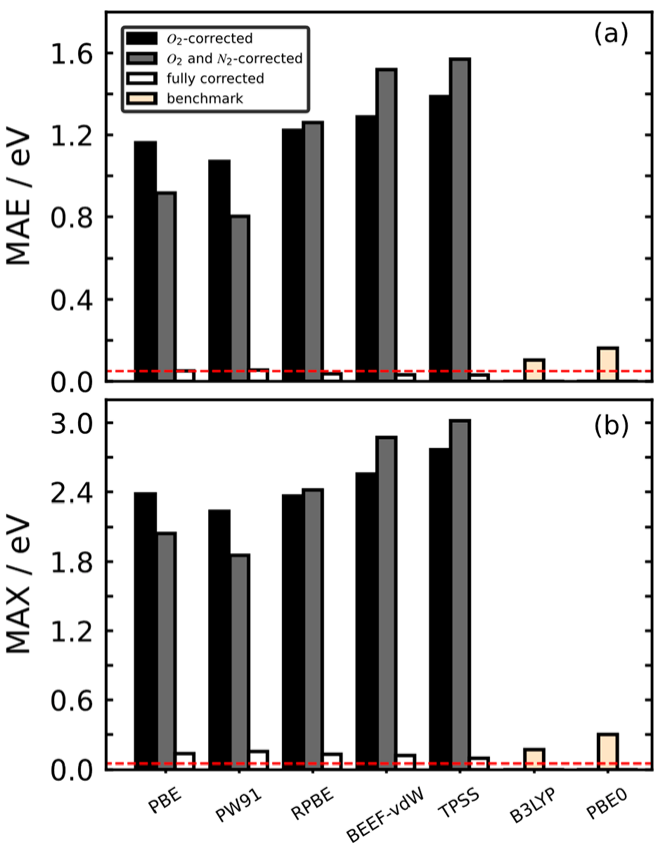
(a) Mean and (b) maximum absolute errors (MAE and MAX) for selected functionals after correcting O_2_ (black), O_2_ and N_2_ (gray), and after applying the correction method based on Table 1 and eq 6 (white) to the nitrogen species in Figure 1. The results for hybrid functionals (B3LYP and PBE0) are used as a benchmark, so that they were corrected in neither panel a nor panel b. The red line represents chemical accuracy (1 kcal/mol).

Figure 3 shows for all functionals under study the MAEs and MAX upon subsequently applying these corrections to the nitrogen species in Figure 1. The final MAEs and MAX of the corrected GGA and meta-GGA functionals are smaller than those of the hybrids and in all cases close to chemical accuracy (1 kcal/mol, red lines in Figure 3). In Figure 3, correcting the error in N_2_ does not necessarily improve the gas-phase errors. Indeed, for PBE and PW91, the errors are lowered after correcting N_2_, but the values for RPBE, BEEF-vdW, and TPSS increase. This behavior is not random but depends on ε_N_2__: if it has the same sign as the errors of the oxidized nitrogen species, ε_N_2__ cancels out a portion of those (eq 5).^40^ In addition, the small change for RPBE stems from its small ε_N_2__ of −0.05 eV.

## Impact on Catalysis

The errors in the previous section are calculated only for gaseous compounds and not for clean or adsorbate-covered active sites. In the following, we will assume that the errors of the active sites with and without adsorbates are similar. This was shown to be a good approximation for the modeling of CO_2_ electroreduction to CO on Cu, Ag, and Au electrodes after applying gas-phase corrections.^30^ However, we cannot discard the idea that significant errors might in some cases subsist after correcting the gas phase.^56^

**Figure 4:**
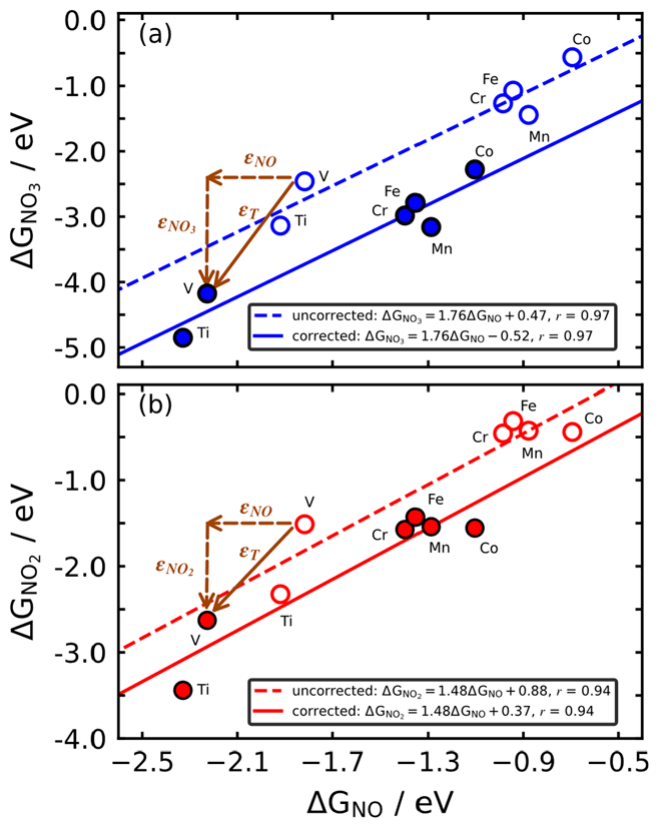
Adsorption energies of (a) NO_3_ and (b) NO_2_ as a function of those of NO on M-porphyrins (M = Ti, V, Cr, Mn, Fe, and Co) calculated with RPBE. Dashed lines and open circles correspond to uncorrected DFT calculations, whereas solid lines and full circles correspond to the results upon gas-phase corrections. The brown arrows show the magnitude and direction of the corrections ε_H_*x*_N_*y*_O_*z*__ for V porphyrin, which are identical for the rest of the materials in the trends.

The importance of the proposed gas-phase corrections for the modeling of catalytic processes within the N cycle is apparent when the adsorption-energy scaling relations^57−59^ among nitrogen oxides are considered. Figure 4 provides the free energies of adsorption of NO_3_ and NO_2_ as a function of that of NO on the metal center of six porphyrins with MN_4_ sites (M: Ti, V, Cr, Mn, Fe, and Co) using RPBE.

Since all the species involved in the scaling relations have an associated gas-phase error, once their energies are corrected, each point is vectorially displaced in the plot. Figure 4 illustrates this for the particular case of a V porphyrin. In Figure 4a the NO correction displaces the data point to the left by 0.41 eV (ε_NO_^RPBE^ = −0.41 eV; see Table S3), while the NO_3_ correction displaces the data point downward by 1.72 eV (ε_NO_3__^RPBE^ = −1.72 eV; Table S3), resulting in a net diagonal displacement of 1.77 eV. In Figure 4b the NO correction is identical and that of NO_2_ displaces the data point downward by 1.12 eV (ε_NO_2__^RPBE^ = −1.12 eV; Table S3), resulting in a net diagonal displacement of 1.19 eV. Since each point in the scaling relation is shifted by a constant amount after applying the corrections, the slopes of the scaling relations remain constant, but the intercepts change. For instance, in Figure 4b the intercept is initially 0.88 eV, and the slope of the uncorrected scaling relation is 1.48 eV. The errors in NO_2_ and NO are −1.12 and −0.41 eV, so that upon corrections one gets an offset of (0.88 – 1.12 + 1.48·0.41) = 0.37 eV.

**Figure 5:**
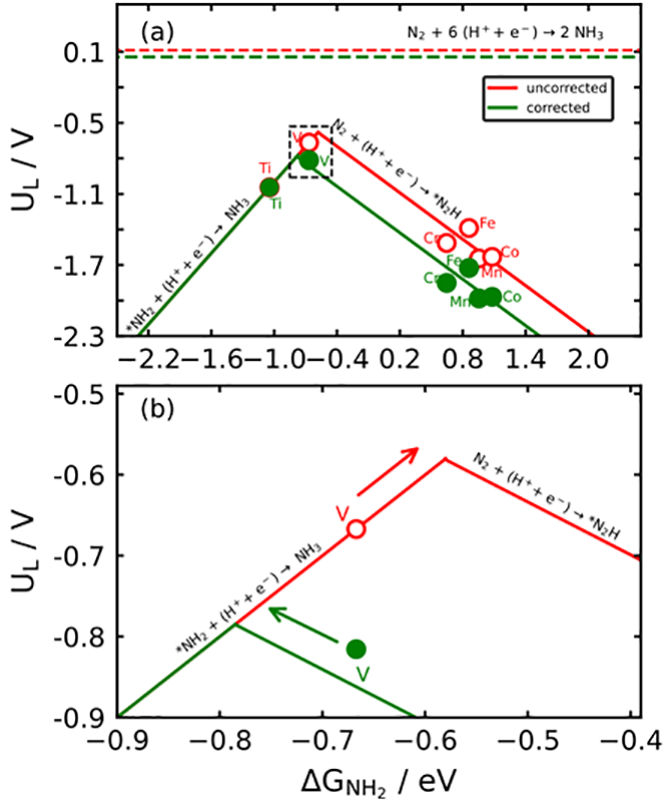
Volcano plot for the electrochemical ammonia synthesis for metalloporphyrins using PBE. We provide a wide-range analysis in panel a, while a focus into the dashed region around the volcano tops is shown in panel b. Red lines and open circles correspond to the uncorrected DFT calculations. Green lines and solid circles represent the results upon correcting the gas-phase errors of N_2_. The red/green dashed lines are the equilibrium potential before/after correcting the N_2_ errors. The arrows in panel b indicate that to reduce the limiting potential, *NH_2_ binding on V porphyrin has to be weakened or strengthened depending on the inclusion or exclusion of gas-phase corrections.

Scaling relations are extensively used to build the so-called Sabatier volcano plots.^58−60^ Those activity plots help find the adsorption energies of key intermediates that ensure optimal catalysis. Because gas-phase corrections modify the offsets of scaling relations (as in Figure 4), the volcano plots based on them are appreciably modified as well. This is exemplified for the electrochemical ammonia synthesis reaction (N_2_ + 6H^+^ + 6*e*^–^ → 2NH_3_) on metalloporphyrins in Figure 5 calculated with PBE.

First of all, in Figure 5a the equilibrium potential of electrochemical ammonia synthesis is presented before (red dashed line) and after (green dashed line) correcting the N_2_ error. Before correcting the equilibrium potential, PBE yields 0.113 V vs RHE. Once the proposed corrections are applied, the equilibrium potential is 0.057 V vs RHE, in agreement with experiments. Admittedly, the error in the equilibrium potentials is not large, but it is amplified by a factor of 6 when assessing the corresponding reaction energies. For PBE this results in a deviation with respect to experiments of the reaction energy as large as 0.34 eV.

As each side of the volcano corresponds to a different electrochemical step, the gas-phase corrections are different. Specifically, the potential on the right leg of the volcano is typically limited by^23^ N_2_^+*^ + H^+^ + *e*^–^ → *N_2_H, which involves the error in N_2_. In turn, the potential on the left leg is usually limited by *NH_2_ + H^+^+*e*^*–*^ → NH_3_+*, which means that the values on that side need not be corrected, as NH_3_ is generally well described by DFT.^25^ Since the legs of the Sabatier volcanoes are based on scaling relations, their slopes are identical before and after correcting the gas-phase errors, yet their offsets vary. This causes the following:

(i) A change in the limiting potential (*U*_*L*_ in V vs RHE; see Table S15). For instance, the limiting potentials of the corrected porphyrins on the right leg of the volcano in Figure 5a (in green) are shifted downward by 0.34 eV with respect to the uncorrected data points (in red), because of the error in N_2_. However, Ti porphyrins experience no change in *U*_*L*_. In turn, V porphyrins experience an intermediate downward shift of 0.15 eV stemming from the switch of potential-limiting steps (Figure 5b).

(ii) A shift in the location of the volcano apex. Such a shift can change activity orderings, the specific volcano leg a material belongs to, and the energy difference between the calculated data points and the apex. This is apparent in Figure 5b for V porphyrin, which switches from the left to the right of the volcano when the gas phase is corrected (see section S5). Before the corrections, V porphyrin is below the volcano apex by 0.09 V. Upon the corrections, it is below by 0.03 V. In terms of Δ*G*_NH_2__, before the corrections V porphyrin is 0.09 eV to the left of the apex. Afterward, it is 0.12 eV to the right. This means that the guidelines for optimizing this porphyrin derived from the two volcanoes are the exact opposite. For comparison, an analogous analysis is provided in Figure S4 for RPBE. We conclude that large shifts in the top of the volcano are normally associated with large gas-phase errors. We emphasize that knowing the precise location of a material with respect to the volcano apex is crucial for its optimization. This has been profusely illustrated for Pt-based catalysts for O_2_ reduction, where Pt(111) is located 0.1 eV to the left of the top in terms of *OH binding energy,^61,62^ such that catalysts are engineered to bind *OH more weakly than Pt(111) by no more than 0.1 eV.

To further illustrate the effect of gas-phase errors in electrocatalysis of the nitrogen cycle, we used RPBE with and without gas-phase corrections to model nitric oxide reduction to hydroxylamine (NO(g) + 4H^+^ + 3*e*^–^ → NH_3_OH^+^(aq)) on porphyrin catalysts. Hydroxylamine is a value-added chemical with numerous uses in industry, such that its electrochemical production from nitrate or NO is an economically appealing way of balancing the N cycle.^63,64^ Generally, we find that the lowest-energy pathway on metalloporphyrins is NO → *NHO → *ONH_2_ → NH_3_OH^+^ (see Table S16).

**Figure 6:**
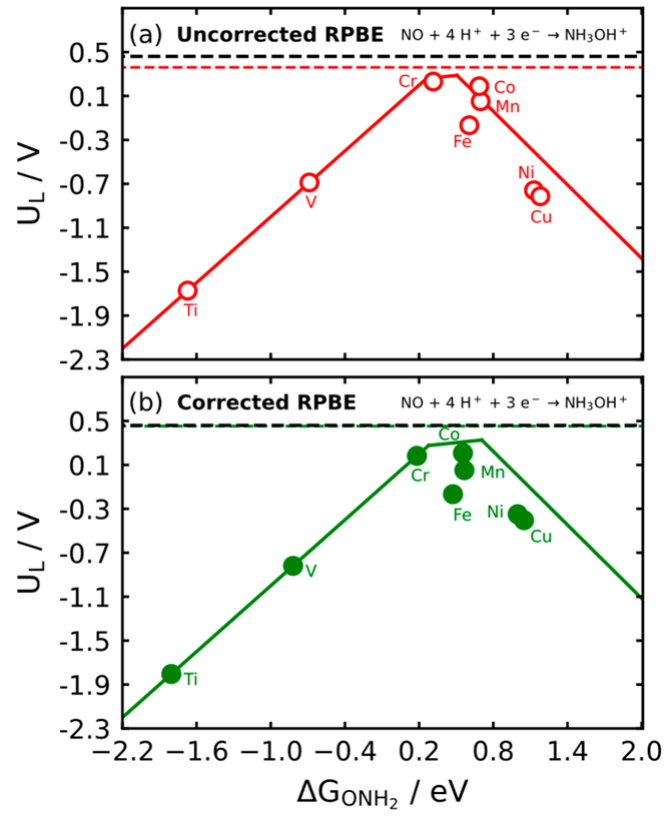
Volcano plot for NO reduction to hydroxylamine on metalloporphyrins calculated with (a) RPBE and (b) RPBE and gas-phase corrections. The red and green dashed lines represent the calculated equilibrium potential, while the black dashed line represents the experimental value. Materials on the left are generally limited by *ONH_2_ hydrogenation, those on the right by NO hydrogenation to *NHO, and those in the middle by *NHO hydrogenation to *ONH_2_; see Table S16.

The energetics of protonated hydroxylamine (NH_3_OH^+^(aq)) was calculated on the basis of its acid–base equilibrium with neutral hydroxylamine (NH_2_OH, p*K*_a_ = 7.68),^65^ the formation free energy of which in the gas phase is −0.03 eV.^66^ Besides, we found a gas-phase error in NH_2_OH of −0.13 eV. As shown in Figure 6, the volcano plot for this reaction has the usual right (weak binding) and left (strong binding) regions and an intermediate binding region. In Figure 6, we observe the following:

(i) The equilibrium potentials and reaction energies change upon applying gas-phase corrections: 0.36 and 0.45 V vs RHE for uncorrected and corrected RPBE, while the experimental value is 0.46 V vs RHE. The respective reaction energies are −1.08, −1.36, and −1.38 eV.

(ii) The ordering of catalytic activities changes upon applying gas-phase corrections. For uncorrected RPBE it is Cr > Co > Mn > Fe > V > Ni > Cu > Ti. Conversely, the ordering for corrected RPBE is Co > Cr > Mn > Fe > Ni > Cu > V > Ti.

(iii) The potential-limiting steps of some materials change upon applying gas-phase corrections. This is the case of the most active porphyrins, namely those of Cr (step 2 vs 3 in uncorrected and corrected RPBE) and Co (step 1 vs 2). Such changes are worth noting, as the recipes for optimizing those materials change significantly (as shown before in Figure 5b).

(iv) Quantitative differences are found for the limiting potentials of the electrocatalysts under study, as shown in Tables S17–S19. The differences are linked to the gas-phase corrections of NO and NH_2_OH (−0.41 and −0.13 eV), which is apparent when the potential-limiting steps are the same with and without gas-phase corrections.

(v) The length of the intermediate binding region changes upon gas-phase corrections. In terms of Δ*G*_ONH_2__, such region has a length of 0.25 and 0.43 eV without and with corrections, respectively.

## Concluding Remarks

Using DFT to predict the gas-phase formation enthalpies of oxidized nitrogen species often results in large negative errors when using GGA and meta-GGA functionals. The DFT errors scale approximately linearly with the number of oxygen atoms in the compounds, with slopes for GGA and meta-GGA functionals close to −0.5 eV/O atom. This is considerably steeper than those found for hybrid functionals (−0.06 and 0.04 eV/O atom for PBE0 and B3LYP). The similar slopes among GGA and meta-GGA functionals indicate that DFT predictions worsen progressively when adding O atoms to the structure and suggest that such errors are probably unavoidable at those rungs of Jacob’s ladder of density functional approximations.^31^

If the data set is subdivided into dinitrogen-containing species (N_2_O_*x*_), mononitrogen-containing species (NO_*x*_), and hydrogenated species (HNO_*x*_), the resulting formation enthalpies can be swiftly corrected with high accuracy. In fact, the MAEs of the GGA and meta-GGA functionals are reduced, on average, from 1.23 to 0.04 eV.

Furthermore, gas-phase errors significantly alter adsorption-energy scaling relations and the volcano plots built upon them. We exemplified that for electrochemical ammonia synthesis and NO reduction to hydroxylamine. The magnitude and direction of the displacements depend on the separate gas-phase errors and the slope of the scaling relations. We noticed changes in (i) the equilibrium potentials and reaction energies, (ii) the location of the volcano peaks, (iii) the location of catalysts on the regions of the volcano, (iv) the predicted limiting potentials, and (v) the catalytic activity orderings. The changes have a direct connection with the magnitudes of the gas-phase errors.

We hope that, as the electrocatalysis of the N cycle regains more and more attention, computational chemists will become increasingly aware of the fact that GGA and meta-GGA functionals have intrinsic gas-phase errors that may impair their catalytic activity, selectivity, and stability predictions. Finally, we note that further experimental and computational efforts are necessary to detect and correct possible errors in the active sites with and without adsorbates.

## Supporting Information Available

Further computational details, tabulated data, assessing and correcting gas-phase errors, additional volcano-type analyses, and direct coordinates of the calculated systems (PDF)
