# Supplementary material for: Impact of Intrinsic Density Functional Theory Errors on the Predictive Power of Nitrogen Cycle Electrocatalysis Models
Source: ACS Catal. 2022 Apr 6;12(8):4784–91. doi: 10.1021/acscatal.1c05333 (PMC9017217; doi:10.1021/acscatal.1c05333)
Supplement: Supplementary file 1 — cs1c05333_si_001.pdf [file cs1c05333_si_001.pdf]

Supplementary Information

Impact of intrinsic DFT errors on the predictive power of nitrogen cycle electrocatalysis models

Ricardo Urrego-Ortiz,[a] Santiago Builes,[a,†] and Federico Calle-Vallejo [b,†]

[a] *Departamento de Ingeniería de Procesos, Universidad EAFIT, Carrera 49 No 7 sur 50, 050022, Medellín, Colombia.*

[b] *Department of Materials Science and Chemical Physics & Institute of Theoretical and Computational Chemistry (IQTCUB), University of Barcelona, C/Martí i Franquès 1, 08028 Barcelona, Spain.*

[†] Emails: sbuiles@eafit.edu.co, f.calle.vallejo@ub.edu

Table of contents

S1. Further computational details and tabulated data 2

S2. Pinpointing and correcting gas-phase errors 8

S3. Structures of the nitrogen oxides and metalloporphyrins 13

S4. Assessing ε_{N_2} 14

S5. Additional volcano-type analyses 14

S6. Direct coordinates 17

S7. References 33

S1. Further computational details and tabulated data

S1.1. Gas-phase calculations: The formation enthalpies of the gaseous compounds were calculated from the elements in their standard states ($N_{2(g)}$, $O_{2(g)}$, $H_{2(g)}$) and were approximated using DFT calculations as follows:

$$\Delta_f H_{DFT} \approx \Delta_f E_{DFT} + \Delta_f ZPE \tag{S1}$$

The zero-point energies (ZPEs) were calculated from the DFT-calculated vibrational frequencies obtained using the harmonic oscillator approximation. Heat capacity contributions to the formation enthalpies were neglected in Equation S1 since their effect is insignificant in the range of 0 to 298.15 K.[1–3] The relaxations were carried out using the conjugate gradient algorithm. All atoms were free to relax in all directions during the geometry optimization and the convergence criterion for the maximal forces on the atoms was 0.01 eV/ Å. The distance between periodic images was at least 10 Å in all the gas-phase calculations. Monkhorst-Pack grids[4] were used to sample the reciprocal space in all calculations, only considering the Γ-point. Ion-electron interactions were described using the projector augmented wave (PAW) method.[5] The kinetic energy cut-off for all the gas-phase calculations was 400 eV, which was shown in previous studies to provide converged reaction energies.[2,6,7] Gaussian smearing with an electronic temperature of 0.001 eV was used. In all cases, the energies were extrapolated to 0 K. We carried out spin unrestricted calculations for the mononitrogen oxides and cis-N_2O_2.

In Table S1, the DFT-calculated formation enthalpies of the nitrogen species studied are shown along with their experimental enthalpies and their calculated ΔZPE values (in parenthesis). In this case, for the (meta-)GGA functionals the DFT-calculated formation enthalpy was computed using the semiempirical correction of $O_{2(g)}$,[2,6,8,9] while for the hybrid functionals (PBE0 and B3LYP) no corrections were included. Table S2 shows the difference between DFT-calculated and experimental formation enthalpies of all the nitrogen oxides studied in this work. This is referred to as ($\varepsilon_{H_xN_yO_z} - \frac{y}{2}\varepsilon_{N_2}$) according to Equation 4 in the main text. This can be thought of as the total error after correcting O_2 ($\varepsilon_T + \frac{z}{2}\varepsilon_{O_2}$, see right-hand side of Equation 4 in the main text).

Table S1. DFT-calculated formation enthalpies ($\Delta_f H_{DFT}$) of 11 nitrogen-containing molecules using the corrected DFT energies of O_2 except for B3LYP and PBE0, which were calculated with no corrections. The experimental formation enthalpies are also shown. The $\Delta_f ZPE$ values are in parenthesis. All values are in eV.

species	PBE	PW91	RPBE	BEEF-vdW	TPSS	PBE0	B3LYP	EXP
N_2O_5	-2.27 (0.31)	-2.12 (0.32)	-2.25 (0.30)	-2.44 (0.29)	-2.65 (0.29)	-0.18 (0.32)	0.29 (0.29)	0.12
N_2O_4	-2.04 (0.26)	-1.92 (0.26)	-2.07 (0.25)	-2.21 (0.24)	-2.33 (0.24)	-0.17 (0.27)	0.18 (0.25)	0.09
N_2O_3	-0.66 (0.16)	-0.58 (0.16)	-0.75 (0.15)	-0.86 (0.15)	-0.96 (0.14)	0.76 (0.16)	0.94 (0.15)	0.86
cis-N_2O_2	0.72 (0.06)	0.84 (0.06)	0.57 (0.05)	0.50 (0.05)	0.46 (0.05)	1.90 (0.05)	1.93 (0.04)	1.77
N_2O	0.01 (0.10)	0.06 (0.10)	0.04 (0.10)	0.07 (0.10)	0.00 (0.08)	0.55 (0.09)	0.72 (0.10)	0.85
NO_3	-0.84 (0.11)	-0.71 (0.11)	-0.95 (0.10)	-1.02 (0.10)	-1.11 (0.09)	0.49 (0.05)	0.68 (0.05)	0.74
NO_2	-0.62 (0.06)	-0.51 (0.06)	-0.75 (0.06)	-0.77 (0.06)	-0.87 (0.05)	0.17 (0.06)	0.28 (0.05)	0.34
NO	0.70 (0.00)	0.80 (0.00)	0.55 (0.00)	0.52 (-0.01)	0.45 (-0.01)	0.92 (-0.01)	0.92 (-0.01)	0.94
HNO_3	-2.52 (0.34)	-2.45 (0.33)	-2.50 (0.33)	-2.57 (0.33)	-2.71 (0.33)	-1.56 (0.34)	-1.23 (0.33)	-1.39
HNO_2	-1.53 (0.23)	-1.49 (0.22)	-1.57 (0.22)	-1.61 (0.22)	-1.70 (0.22)	-0.86 (0.23)	-0.71 (0.23)	-0.82
HNO	0.81 (0.11)	0.83 (0.11)	0.76 (0.11)	0.76 (0.11)	0.71 (0.11)	1.09 (0.12)	1.15 (0.11)	1.03

Table S2. Total error in the DFT-calculated formation enthalpies (in eV) of the 11 molecules studied in this work using the corrected DFT energies of O_2 except for B3LYP and PBE0, for which no corrections are used. The respective total errors ($\varepsilon_{H_xN_yO_z} - \frac{y}{2}\varepsilon_{N_2}$) are calculated using Equation 4 in the main text. The MAEs and MAX are also shown.

species	PBE	PW91	RPBE	BEEF-vdW	TPSS	PBE0	B3LYP
N_2O_5	-2.38	-2.24	-2.36	-2.55	-2.77	-0.30	0.17
N_2O_4	-2.14	-2.02	-2.17	-2.30	-2.42	-0.27	0.08
N_2O_3	-1.52	-1.44	-1.61	-1.72	-1.82	-0.09	0.08
cis-N_2O_2	-1.05	-0.93	-1.20	-1.27	-1.31	0.13	0.16
N_2O	-0.84	-0.79	-0.81	-0.78	-0.86	-0.30	-0.13
NO_3	-1.58	-1.45	-1.69	-1.76	-1.85	-0.24	-0.06
NO_2	-0.97	-0.85	-1.09	-1.12	-1.22	-0.18	-0.06
NO	-0.24	-0.14	-0.38	-0.42	-0.49	-0.01	-0.02
HNO_3	-1.13	-1.06	-1.11	-1.19	-1.33	-0.17	0.16
HNO_2	-0.71	-0.67	-0.75	-0.79	-0.88	-0.03	0.12
HNO	-0.22	-0.20	-0.27	-0.27	-0.32	0.06	0.12
MAE	1.16	1.07	1.22	1.29	1.39	0.16	0.10
MAX	2.38	2.24	2.36	2.55	2.77	0.30	0.17

Table S3 shows the isolated gas-phase errors of the nitrogen compounds in this work ($\varepsilon_{H_xN_yO_z}$). These errors are obtained after correcting the O_2 and N_2 errors (see Equation 5 in the main text). The MAE and MAX are shown for each functional. The error in N_2 is calculated from the ammonia synthesis reaction, as explained in previous works and detailed in section S4.[6] In all cases, the mean and maximum absolute errors (MAE and MAX) are reported.

Table S3. Isolated errors ($\varepsilon_{H_xN_yO_z}$) of 11 nitrogen-containing molecules, obtained after applying the N_2 correction to the values in Table S2 (which already incorporate the O_2 correction). The MAE and MAX are shown in each case. All values are in eV.

	PBE	PW91	RPBE	BEEF-vdW	TPSS
N_2O_5	-2.04	-1.86	-2.42	-2.87	-3.02
N_2O_4	-1.80	-1.63	-2.22	-2.62	-2.67
N_2O_3	-1.19	-1.06	-1.66	-2.04	-2.07
cis-N_2O_2	-0.71	-0.55	-1.25	-1.59	-1.56
N_2O	-0.50	-0.41	-0.86	-1.10	-1.11
NO_3	-1.41	-1.26	-1.72	-1.92	-1.98
NO_2	-0.80	-0.66	-1.12	-1.27	-1.34
NO	-0.07	0.05	-0.41	-0.58	-0.62
HNO_3	-0.96	-0.87	-1.14	-1.35	-1.45
HNO_2	-0.54	-0.48	-0.78	-0.95	-1.00
HNO	-0.05	-0.01	-0.30	-0.43	-0.45
MAE	0.92	0.80	1.26	1.52	1.57
MAX	2.04	1.86	2.42	2.87	3.02

S1.2. Adsorption energies: the computational details of the calculations on porphyrins are the same as for the gas-phase calculations (code, convergence criteria, energy cutoff, etc.). All porphyrins were calculated in boxes of 20 Å × 20 Å × 20 Å. The free energies of adsorption were calculated using Equation S2:

$$\Delta G_{DFT} \approx \Delta E_{DFT} + \Delta ZPE - T\Delta S \quad \text{(S2)}$$

The gas-phase entropies were taken from experiments[10,11] while for the adsorbed species only their vibrational entropy contribution was considered. Both the vibrational entropies and the

ZPEs were obtained using the DFT-calculated vibrational frequencies. The free energies of adsorption of the NO_x species (x = 1, 2, 3) on the metalloporphyrins were calculated with respect to their gas-phase counterparts, as shown in Equation S3:

$$* + NO_{x\,(g)} \rightarrow * NO_x \tag{S3}$$

where * refers to the clean metalloporphyrin and $*NO_x$ denotes the adsorbed mononitrogen species. These adsorption free energies were used to construct the scaling relations in Figure 4 in the main text. Similarly, Equations S4 and S5 were used to construct the volcano plots in Figure 5 of the main text:

$$* + N_{2\,(g)} + H^+ + e^- \rightarrow * N_2H \tag{S4}$$

$$* NH_2 + H^+ + e^- \rightarrow NH_{3\,(g)} + * \tag{S5}$$

For Equations S4 and S5, the proton-electron transfers were described using the computational hydrogen electrode.[9] Spin-unrestricted calculations were carried out for metalloporphyrins with and without adsorbates. In each case, several spin values were evaluated, and the most stable state was selected. Table S4 shows such spin states when using PBE and RPBE; Table S5 shows the ZPEs of the gas-phase molecules in Equations S3-S5 calculated with PBE and RPBE, and their experimental TS values.[10,11] Table S6 contains the ZPEs of the adsorbed species in Equations S3-S5 calculated with RPBE and PBE. Table S7 shows the TS values of the adsorbed species involved in Equations S3-S5 calculated with RPBE and PBE. Tables S8 and S9 contain ΔE_{DFT} for several adsorbed species and electrochemical steps.

Table S4. Most stable spin states (in μ_B) for all metalloporphyrins under study with and without adsorbates.

	RPBE						PBE			
porphyrin metal	clean	*NO	$*NO_2$	$*NO_3$	$*NH_2$	$*N_2H$	clean	*NO	$*NH_2$	$*N_2H$
Co	1	0	0	0	0	0	1	0	0	0
Cr	4	1	3	3	3	1	4	1	3	1
Fe	2	1	1	3	1	1	2	1	1	1
Mn	3	0	4	4	2	0	3	0	2	0
V	3	0	2	2	2	2	3	0	2	2
Ti	2	1	1	1	1	1	2	1	1	1

Table S5. ZPE and TS values of the gaseous species involved in Equations S3–S5. The entropy values were taken from experimental data[10,11] at 298.15 K. All values are in eV.

	RPBE	PBE	EXP
species	ZPE	ZPE	TS
NO_3	0.32	0.33	0.78
NO_2	0.23	0.23	0.74
NO	0.12	0.12	0.65
H_2	0.27	0.27	0.40
N_2	0.15	0.15	0.59
NH_3	0.91	0.91	0.60

Table S6. ZPEs of the adsorbed species in Equations S3–S5. All values are in eV.

porphyrin	RPBE					PBE		
metal center	*NO	*NO_2	*NO_3	*NH_2	*N_2H	*NO	*NH_2	*N_2H
Co	0.17	0.31	0.40	0.67	0.48	0.18	0.67	0.48
Cr	0.21	0.28	0.40	0.65	0.47	0.21	0.65	0.47
Fe	0.18	0.29	0.39	0.67	0.47	0.19	0.67	0.47
Mn	0.21	0.24	0.39	0.66	0.48	0.22	0.67	0.48
V	0.19	0.27	0.42	0.66	0.46	0.20	0.66	0.45
Ti	0.17	0.27	0.41	0.64	0.44	0.18	0.64	0.48

Table S7. TS values of the adsorbed species in Equations S3–S5. All values are in eV.

porphyrin	RPBE					PBE		
metal center	*NO	*NO_2	*NO_3	*NH_2	*N_2H	*NO	*NH_2	*N_2H
Co	0.10	0.21	0.27	0.13	0.16	0.10	0.12	0.11
Cr	0.13	0.18	0.29	0.15	0.17	0.12	0.14	0.17
Fe	0.09	0.24	0.31	0.11	0.10	0.15	0.11	0.22
Mn	0.12	0.19	0.32	0.12	0.18	0.11	0.11	0.12
V	0.14	0.16	0.25	0.12	0.16	0.12	0.12	0.19
Ti	0.15	0.20	0.26	0.14	0.20	0.14	0.13	0.14

Table S8. Adsorption energies (ΔE_{DFT}) for each porphyrin in Equation S3 calculated with RPBE and PBE. All values are in eV.

	RPBE			PBE
porphyrin metal center	$\Delta E_{DFT,*NO}$	$\Delta E_{DFT,*NO_2}$	$\Delta E_{DFT,*NO_3}$	$\Delta E_{DFT,*NO}$
Co	-1.30	-1.05	-1.16	-1.61
Cr	-1.60	-1.07	-1.83	-2.01
Fe	-1.56	-0.87	-1.61	-1.90
Mn	-1.50	-0.98	-1.97	-1.92
V	-2.40	-2.13	-3.08	-2.76
Ti	-2.47	-2.90	-3.74	-2.76

Table S9. Reaction energies (ΔE_{DFT}) of Equations S4 and S5 calculated with RPBE and PBE. All values are in eV.

	RPBE		PBE	
porphyrin metal center	$\Delta E_{DFT,eq.S4}$	$\Delta E_{DFT,eq.S5}$	$\Delta E_{DFT,eq.S4}$	$\Delta E_{DFT,eq.S5}$
Co	-1.09	1.10	-0.91	0.75
Cr	-0.68	1.20	-0.52	0.71
Fe	-0.88	1.06	-0.68	0.63
Mn	-0.98	1.28	-0.78	0.78
V	0.67	0.08	0.83	-0.29
Ti	1.01	-0.15	1.17	-0.55

S1.3. ZPE analysis: the ZPEs necessary for the computation of the DFT-formation enthalpies (Equation S1), might contribute significantly to the gas-phase errors. If that were the case, the gas-phase errors could be further decomposed into those associated to the DFT total energies and those associated to the ZPEs. First of all, the values in parenthesis in Table S1 show that the calculated ZPE of a given molecule is similar for all the functionals, regardless of the level of theory (GGA, meta-GGA, hybrid). Thus, in the following, we will compare experimental values taken from refs[12,13] and PBE-calculated values only. Table S10 shows that the DFT-calculated ZPEs of all substances in this study are remarkably similar to the experimental values, such that the average difference is as low as 0.01 eV, and the maximum difference is only 0.02 eV. In the light of these results, we are confident that, as a first approximation, a further decomposition of the gas-phase errors into those arising from electronic energies and those from ZPEs is not necessary.

Table S10. Experimental and DFT-calculated (with PBE) zero-point energies of the molecules in this study and their absolute differences. All values are in eV. MAE: mean absolute error. NF: not found.

molecule	ZPE_{exp}	ZPE_{DFT}	$\lvert ZPE_{exp} - ZPE_{DFT} \rvert$
N_2	0.15	0.15	0.01
O_2	0.10	0.10	0.00
H_2	0.27	0.27	0.00
NH_3	0.89	0.91	0.02
N_2O_5	NF	0.71	-
N_2O_4	0.61	0.61	0.01
N_2O_3	0.44	0.45	0.01
cis-N_2O_2	0.28	0.31	0.02
N_2O	0.29	0.30	0.01
NO_3	0.34	0.35	0.01
NO_2	0.23	0.23	0.00
NO	0.12	0.12	0.00
HNO_3	0.70	0.69	0.00
HNO_2	0.53	0.53	0.01
HNO	0.36	0.37	0.01
MAE			0.01

S2. Pinpointing and correcting gas-phase errors

S2.1. Accounting for the N_2 error to pinpoint other errors: to be able to correct the gas-phase errors of the nitrogen-containing species, it is first necessary to identify and isolate the error in N_2. In line with previous works,[6] we consider here the formation reaction to be:

$$\frac{x}{2}H_2 + \frac{y}{2}N_2 + \frac{z}{2}O_2 \rightarrow H_xN_yO_z \tag{S6}$$

The total error for H_2 is usually negligible and that of O_2 is large but easily corrected in a semiempirical way by means of H_2 and H_2O.[2,7,9] Once O_2 is corrected, the error in the DFT-calculated formation enthalpy of a gaseous nitrogen-containing compound $H_xN_yO_z$ can be expressed as a combination of the N_2 error and the error of the compound itself. These errors appear in Table S2. Thus, once the error in N_2 is corrected, that of $H_xN_yO_z$ is isolated, see Table S3 and Equation S7.

$$\varepsilon_{H_xN_yO_z} = \Delta_f H^{DFT}_{H_xN_yO_z} - \Delta_f H^{EXP}_{H_xN_yO_z} + \frac{z}{2}\varepsilon_{O_2} + \frac{y}{2}\varepsilon_{N_2} \tag{S7}$$

where $\Delta_f H^{DFT}_{H_xN_yO_z} - \Delta_f H^{EXP}_{H_xN_yO_z} + \frac{z}{2}\varepsilon_{O_2}$ is the total error in the formation enthalpy of the compound calculated with the corrected energies of O_2 (i.e., the difference between the experimental and DFT-calculated enthalpies once O_2 is corrected, see the values in Table S2), $\frac{y}{2}$ is the stoichiometric coefficient of N_2 in the formation reaction (Equation 1 in the main text and Equation S6 in this document). $\frac{y}{2} = 1$ for N_2O_x, and 0.5 for NO_x and HNO_x. Finally, ε_{N_2} is the error in N_2 calculated from the ammonia synthesis reaction.[6] Once the errors are isolated, the molecules are assigned to the corresponding chemical groups. Then, the errors are correlated with the number of oxygen atoms in the structure, as shown in Figure S1 for all the GGAs. Importantly, although there is an overall linear trend (Figure 2 in the main text), dividing them into groups allows for a more accurate correction scheme.

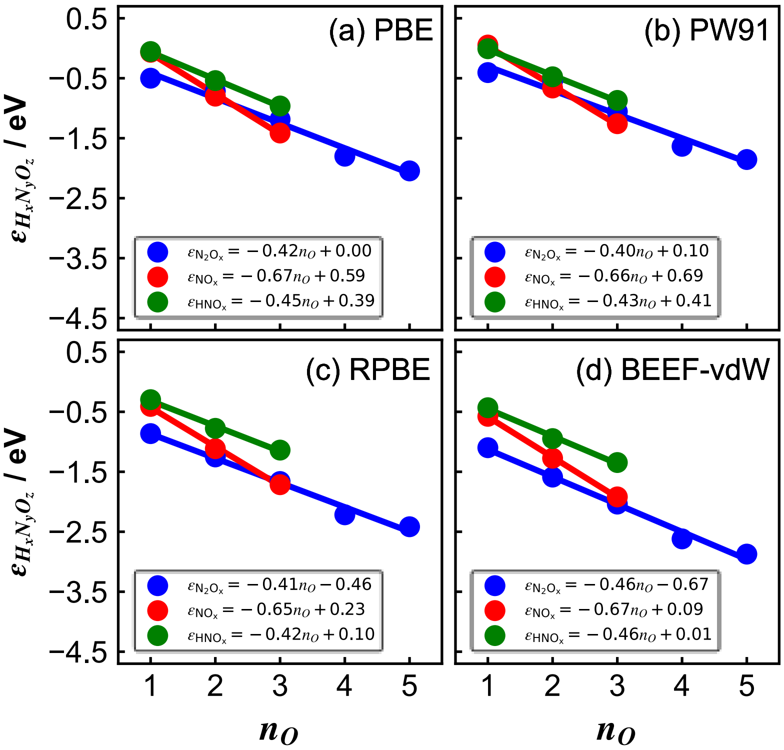

Figure S1. Trends in the isolated errors of each group of nitrogen-containing compounds as a function of their number of oxygen atoms for (a) PBE, (b) PW91, (c) RPBE, and (d) BEEF-vdW. Blue, red, and green correspond to N_2O_x, NO_x and HNO_x, respectively. The regression equations (reported also in Table 1) are provided in each panel.

The linear dependence in Figure S1 allows us to predict the DFT-based error of each compound in our dataset by simply: (i) counting the number of oxygen atoms it contains, (ii)

identifying its chemical group, and (iii) using the corresponding parameters in Figure S1. For instance, the PBE error (in eV) of a nitrogen oxide $H_xN_yO_z$ belonging to the N_2O_x group is:

$$\varepsilon_{N_2O_x} \approx -0.42 \cdot n_O + 0.00 \tag{S8}$$

where n_O is the number of oxygen atoms in the molecule, and -0.42 eV/O atom and 0.00 eV are the respective slope and intercept of the group in Figure S1a. The slopes and intercepts in Figure S1a are reported in Table 1 of the main text for the group N_2O_x using PBE.

For clarity, the procedure used to find the parameters of the HNO_x group with RPBE is also detailed in the following. First, Equation S7 is applied to all members of the HNO_x group. For this group, $\frac{y}{2} = 0.5$. In addition, $\varepsilon_{N_2} = -0.05$ eV.[6] After correcting the errors in O_2 and N_2, the errors of all hydrogenated species can be isolated. Next, the DFT errors of these compounds are correlated with their number of oxygen atoms. This is shown in panel (c) of Figure S1 along with the linear fit: the slope and the intercept are -0.42 eV/O atom and 0.10 eV and are reported in Table 1. In Table S11 the residual errors of each N-compound after applying the corrections are summarized for all functionals, except PBE0 and B3LYP, which were not corrected.

Table S11. Residual errors of the nitrogen compounds after correcting their energies with the values in Table 1 of the main text. MAE and MAX values are provided in each case. All values are in eV.

Species	PBE	PW91	RPBE	BEEF-vdW	TPSS
N_2O_5	0.04	0.04	0.08	0.09	0.06
N_2O_4	-0.13	-0.14	-0.13	-0.12	-0.09
N_2O_3	0.06	0.04	0.02	0.00	0.01
cis-N_2O_2	0.12	0.15	0.02	0.00	0.03
N_2O	-0.09	-0.10	0.00	0.03	-0.01
NO_3	0.02	0.02	0.02	0.01	0.02
NO_2	-0.04	-0.04	-0.04	-0.02	-0.03
NO	0.02	0.02	0.02	0.01	0.02
HNO_3	0.01	0.01	0.02	0.02	0.02
HNO_2	-0.02	-0.02	-0.04	-0.04	-0.04
HNO	0.01	0.01	0.02	0.02	0.02
MAE	0.05	0.06	0.04	0.03	0.03
MAX	0.13	0.15	0.13	0.12	0.09

S2.2. Corrections using the linear trends in Figure 2: it is possible to use the linear trends in Figure 2a in the main text to correct the DFT errors in the formation enthalpy of the nitrogen-containing compounds. However, the residual errors of this approach are sizable. We provide in Table S12 the slope and intercept of the lines in Figure 2a of the main text. The MAE and MAX after using these lines to correct the errors in the nitrogen oxides are also shown. Similarly, Table S13 summarizes the results when the trends in Figure 2b are used to correct the errors.

Table S12. Parameters of the lines in Figure 2a of the main text, and residual errors obtained after using them to correct the DFT-calculated formation enthalpies of the nitrogen compounds. The slopes are in eV/O atom, and the intercepts, MAE and MAX in eV.

functional	slope	intercept	MAE	MAX
PBE	-0.51	0.09	0.18	0.42
PW91	-0.49	0.13	0.17	0.43
RPBE	-0.49	-0.01	0.20	0.38
BEEF-vdW	-0.54	0.04	0.20	0.40
TPSS	-0.57	0.01	0.18	0.37

The average of the MAEs is ~0.18 eV and that of the MAX is ~0.40 eV, which are larger than those found with the aforementioned classification method (average MAE and MAX of 0.04 and 0.12 eV, Table S11).

Table S13. Parameters of the lines in Figure 2b of the main text, and residual errors obtained after using them to correct the DFT-calculated formation enthalpies of the nitrogen compounds. The slopes are in eV/O atom, and the intercepts, MAE and MAX in eV.

	slope	intercept	MAE	MAX
PBE	-0.48	0.27	0.14	0.29
PW91	-0.46	0.34	0.13	0.28
RPBE	-0.50	-0.04	0.21	0.39
BEEF-vdW	-0.57	-0.13	0.23	0.48
TPSS	-0.59	-0.12	0.21	0.44

In this case, the average of the MAEs is 0.18 eV and that of the MAX is 0.38 eV, which are large compared to those obtained using the classification method.

We note that the average of the MAX decreased slightly from the values in Table S12 (from 0.40 to 0.38 eV) and the average of the MAE remained at 0.18 eV. This is because of a

compensation effect, as the MAE and MAX worsened after the N_2 correction for RPBE, BEEF-vdW and TPSS, while those of PBE and PW91 improved. This occurs because the error in N_2 for RPBE, BEEF-vdW and TPSS has the same sign as the errors of the nitrogen oxides and, therefore, partially cancels them out when it is not accounted for. This behavior was previously reported for organic nitrates and nitrites.[6]

S2.3. Using the gas-phase corrections for other reactions: our approach finds the error in the total DFT energy of a compound based on its formation energy (Equation 5 in the main text). For example, the formation reaction of dinitrogen pentoxide is:

$$N_2 + \frac{5}{2}O_2 \rightarrow N_2O_5 \quad \text{(S9)}$$

Once the errors in N_2 (via $\frac{1}{2}N_2 + \frac{3}{2}H_2 \rightarrow NH_3$) and O_2 (via $H_2 + \frac{1}{2}O_2 \rightarrow H_2O$) are semiempirically corrected, the error in the heat of formation of N_2O_5 with respect to experiments is -2.04 eV for PBE, see Table S3. This means that subtracting as much as -2.04 eV from the total energy of N_2O_5 should lead to a corrected formation energy in agreement with experiments. Once this is done, it is possible to combine different gas-phase species and still obtain accurate gas-phase energies. Let us consider the following reaction and the PBE functional:

$$NO_2 + NO_3 \rightarrow N_2O_5 \quad \text{(S10)}$$

The enthalpy of this reaction can be written in terms of the DFT-calculated enthalpies as:

$$\Delta_r H = H_{DFT,N_2O_5} - H_{DFT,NO_2} - H_{DFT,NO_3} \quad \text{(S11)}$$

Equation S11 can also be written in terms of formation enthalpies ($\Delta_r H = \Delta H_{DFT,N_2O_5} - \Delta H_{DFT,NO_2} - \Delta H_{DFT,NO_3}$) and the analysis would be identical. The corrected formation enthalpy ($\Delta_r H_{corr}$ in eV) for PBE is obtained by subtracting to each term the corresponding error (see Table S3):

$$\Delta_r H_{corr} = [H_{DFT,N_2O_5} - (-2.04)] - [H_{DFT,NO_2} - (-0.80)] - [H_{DFT,NO_3} - (-1.41)] \quad \text{(S12)}$$

$$\Delta_r H_{corr} = H_{DFT,N_2O_5} - H_{DFT,NO} - H_{DFT,NO_2} - 0.17 \quad \text{(S13)}$$

With Equation S13, the total energies and the ZPEs of the nitrogen oxides we find that $\Delta_r H_{corr} = -0.96\ eV$, which is identical to the experimental value.

S3. Structures of the nitrogen oxides and metalloporphyrins

Figure S2 shows the single, double and triple bonds present in the oxidized nitrogen-based species in this study.

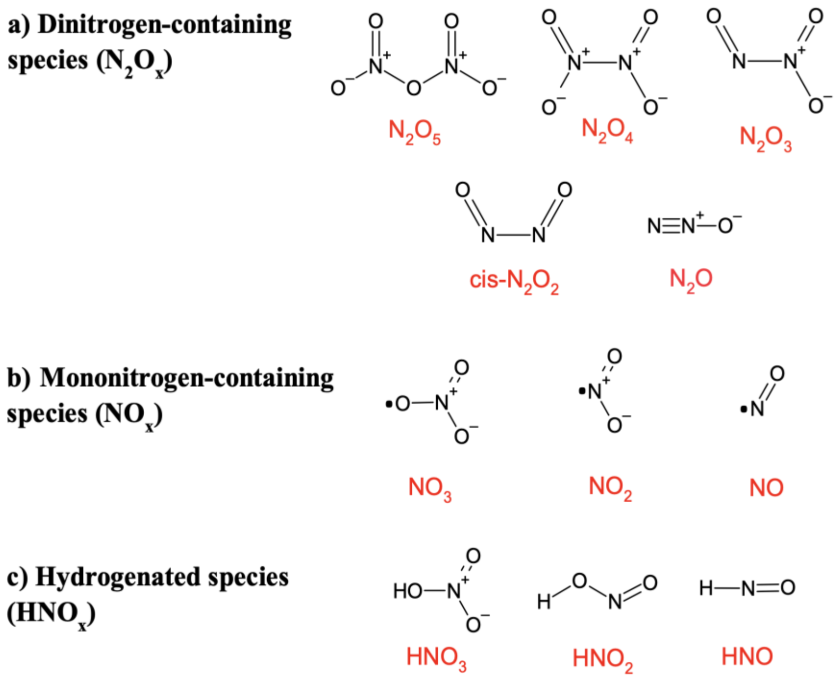

Figure S2. Nitrogen-containing species studied in this work. The dots in (b) represent unpaired electrons. The positive and negative signs on the nitrogen and oxygen atoms represent their respective formal charge.

In Figure S3, the structure of the metalloporphyrins analyzed is presented. In all cases, the metallic centers and the nitrogen atoms are in a planar square arrangement and the ring is flat.

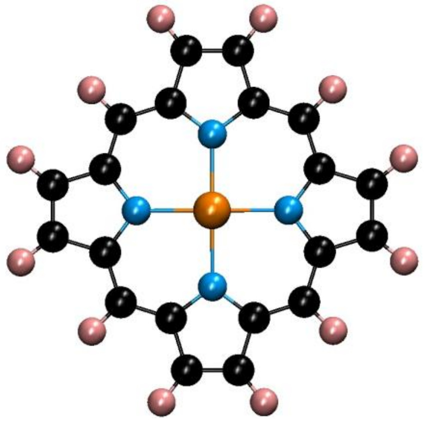

Figure S3. Structure of the studied metalloporphyrins. The metal center (M: Ti, V, Cr, Mn, Fe, Co) is shown in orange, N atoms in blue, C atoms in black, and H atoms in pink.

S4. Assessing ε_{N_2}

N_2 is a reactant in the formation reaction of all oxidized nitrogen compounds (Equation 1 in the main text), so it contributes to the total error in the DFT-calculated formation enthalpy. Because DFT tends to accurately predict the energetics of NH_3 and H_2,[14] the gas-phase ammonia synthesis reaction (ASR, $0.5\,N_2 + 1.5\,H_2 \rightarrow NH_3$) can be used to estimate the error in N_2. To do so, we first employ the definition of the total error (ε_T) shown in the main text (Equation 2) for the ASR:

$$\varepsilon_{ASR}^{T} = \Delta_f H_{ASR}^{DFT} - \Delta_f H_{ASR}^{EXP} \tag{S14}$$

where $\varepsilon_{T,ASR}$ corresponds to the individual errors of the reactants and the products according to their stoichiometry, in the following way:

$$\varepsilon_{ASR}^{T} = \varepsilon_{NH_3} - 1.5\,\varepsilon_{H_2} - 0.5\,\varepsilon_{N_2} \tag{S15}$$

Since H_2 and NH_3 are likely well-described ($\varepsilon_{NH_3} \approx \varepsilon_{H_2} \approx 0$)[14] the error in N_2 can be expressed by combining Equations S14 and S15:

$$\varepsilon_{N_2} \approx -2 \cdot \varepsilon_{ASR}^{T} = -2 \cdot (\Delta_f H_{ASR}^{DFT} - \Delta_f H_{ASR}^{EXP}) \tag{S16}$$

For PBE, for instance, the DFT-calculated formation enthalpy of the ASR is -0.64 eV while the experimental value is -0.48 eV.[10] From Equation S14: $\varepsilon_{ASR}^{T,PBE} = -0.64\,eV - (-0.48\,eV) = -0.17\,eV$, and from Equation S16: $\varepsilon_{N_2}^{PBE} = -2 \cdot (-0.17\text{ eV}) = 0.34\,eV$. This procedure was performed for all exchange-correlation functionals corrected (i.e., all the studied xc-functionals except for the hybrids). In Table S14, the calculated N_2 errors are shown for the meta-GGA and GGA functionals.

Table S14. ε_{N_2} values calculated for the meta-GGA and GGA functionals analyzed.[6] All values are in eV.

PBE	PW91	RPBE	BEEF-vdW	TPSS
0.34	0.38	-0.05	-0.32	-0.25

S5. Additional volcano-type analyses

S5.1. Electrochemical ammonia synthesis: in Table S15 we list the differences in the limiting potentials before and after applying gas-phase corrections for PBE.

Table S15. Differences in the limiting potentials of metalloporphyrins for ammonia synthesis reaction calculated with PBE with and without gas-phase corrections.

Metal center	$U_L^{corrected} - U_L^{uncorrected}$ / V
Ti	0.00
V	-0.15
Cr	-0.34
Mn	-0.34
Fe	-0.34
Co	-0.34

Furthermore, Figure S4 is analogous to Figure 5 in the main text but for RPBE. The differences between the corrected and uncorrected volcanoes are not large, in view of the small gas-phase error of N_2 (-0.05 eV).[6]

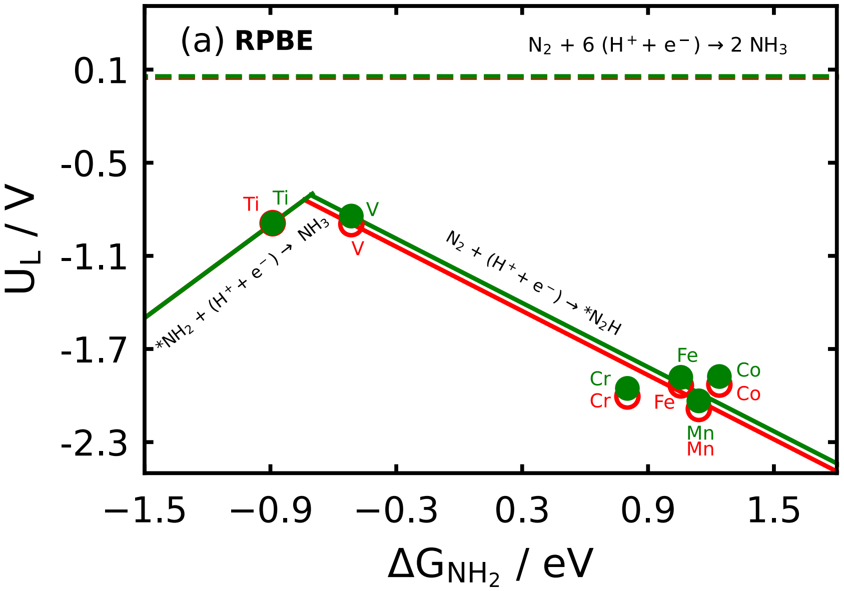

Figure S4. Volcano plot for electrochemical ammonia synthesis on metalloporphyrins using (a) RPBE and (b) PBE. Red lines and open circles correspond to the uncorrected DFT calculations. Green lines and solid circles correspond to results upon correcting the gas phase errors of N_2. The red/green dashed lines are the equilibrium potential before/after correcting the N_2 errors.

S5.2. Nitric oxide reduction to hydroxylamine: in Table S16 we report the pathways and intermediates of the nitric oxide reduction to hydroxylamine for each metalloporphyrin. In Table

S17, the electrochemical potential for each step is shown, calculated without gas-phase corrections. In Table S17, we present the electrochemical potentials including gas-phase corrections. In Tables S17 and S18, we mark the potential-limiting steps and the activity ordering of the metal centers in the NO reduction to hydroxylamine, where 1 corresponds to the most active center and 8 refers to the least active metal. Importantly, some of the limiting steps, their actual potentials, and the activity ordering change after including gas-phase corrections. The differences in limiting potentials are shown in Table S19.

Table S16. Most thermodynamically favorable pathways for the electrochemical reduction of nitric oxide to hydroxylamine on different metalloporphyrins. The most common pathway is: NO→*NHO→*ONH_2→NH_3OH^+.

active center	1st	2nd	3rd	4th
Ti	NO	*NHO	*ONH_2	NH_3OH^+
V	NO	*NOH	*HNOH	NH_3OH^+
Cr	NO	*NHO	*ONH_2	NH_3OH^+
Mn	NO	*NHO	*ONH_2	NH_3OH^+
Fe	NO	*NHO	*HNOH	NH_3OH^+
Co	NO	*NHO	*HNOH	NH_3OH^+
Ni	NO	*NHO	*ONH_2	NH_3OH^+
Cu	NO	*NHO	*ONH_2	NH_3OH^+

Table S17. Potentials (in V vs RHE) of each step (U_1-U_3) for the reduction of NO to hydroxylamine calculated with RPBE and no gas-phase corrections. The limiting potential (U_L) is marked in bold in each case. The last column provides a catalytic activity ordering for each metallic center, where 1 is the most active site and 8 is the least active.

active center	U_1	U_2	U_3	activity ordering
Ti	2.56	0.20	**-1.67**	8
V	1.62	0.15	**-0.69**	5
Cr	0.54	**0.23**	0.31	1
Mn	0.33	**0.05**	0.70	3
Fe	0.64	**-0.17**	0.61	4
Co	**0.19**	0.21	0.69	2
Ni	**-0.76**	0.71	1.13	6
Cu	**-0.81**	0.72	1.18	7

Table S18. Potentials (in V vs RHE) of each step (U_1-U_3) for the reduction of NO to hydroxylamine calculated with RPBE and gas-phase corrections. The limiting potential (U_L) is marked in bold in each case. The last column provides a catalytic activity ordering for each metallic center, where 1 is the most active site and 8 is the least active.

active center	U_1	U_2	U_3	activity ordering
Ti	2.97	0.20	**-1.80**	8
V	2.03	0.15	**-0.82**	7
Cr	0.95	0.23	**0.18**	2
Mn	0.74	**0.05**	0.57	3
Fe	1.05	**-0.17**	0.47	4
Co	0.60	**0.21**	0.55	1
Ni	**-0.35**	0.71	1.00	5
Cu	**-0.40**	0.72	1.05	6

Table S19. Differences in the limiting potentials of metalloporphyrins for NO reduction to NH_3OH^+ calculated with RPBE with and without gas-phase corrections.

Metal center	$U_L^{corrected} - U_L^{uncorrected}$ / V
Ti	-0.13
V	-0.13
Cr	-0.05
Mn	0.00
Fe	0.00
Co	0.02
Ni	0.41
Cu	0.41

S6. Direct coordinates

The CONTCAR files below contain the optimized geometries of the nitrogen oxides calculated with PBE, and the optimized geometries of metalloporphyrins, with and without adsorbates, calculated with RPBE. The degrees of freedom were set to T T T for all atoms in all systems.

S6.1 Nitrogen oxides for PBE

N2O5
```
   1.00000000000000
    15.00    0.00    0.00
     0.00   15.00    0.00
     0.00    0.00   15.00
   O    N
     5    2
Selective dynamics
Direct
  0.1154407139533819  0.1056361897031214  0.0001001084789832
  0.9676817827853208  0.0987498647150652  0.0221791138251037
  0.2631658319494841  0.0988965878207171  0.9777117809863548
  0.0317002111482843  0.9771091554121469  0.9678350493056126
  0.1993636330092183  0.9771901829951601  0.0321729701480460
  0.0279079458727255  0.0518442367599607  0.9969373583111291
  0.2030332146149170  0.0519337825938268  0.0030702856114347
```

N2O4
```
   1.00000000000000
    15.00    0.00    0.00
     0.00   15.00    0.00
     0.00    0.00   15.00
   O    N
     4    2
Selective dynamics
Direct
  0.0342096922403837  0.0742215208314732  0.0000000000000000
  0.0342096922403837  0.9257784791685268  0.0000000000000000
  0.8469903077596158  0.0742215208314732  0.0000000000000000
  0.8469903077596158  0.9257784791685268  0.0000000000000000
  0.0031144441312577 -0.0000000000000000  0.0000000000000000
  0.8780855558687423 -0.0000000000000000  0.0000000000000000
```

N2O3
```
   1.00000000000000
    15.00    0.00    0.00
     0.00   15.00    0.00
     0.00    0.00   15.00
   O    N
     3    2
Selective dynamics
Direct
  0.0138421516542262  0.8778175362199636  0.0000000000000000
  0.0812071082100484  0.0436041655467427  0.0000000000000000
  0.9470346092515177  0.1060190712727007  0.0000000000000000
  0.9521600616827408  0.9243621076341101  0.0000000000000000
 -0.0001105974651955  0.0443171193264800  0.0000000000000000
```

cis-N2O2
```
   1.00000000000000
    15.00    0.00    0.00
```

```
   0.00   15.00    0.00
   0.00    0.00   15.00
  O    N
   2     2
Selective dynamics
Direct
 0.0000000000000000  0.0945027692355312  0.9660414097851315
 0.0000000000000000  0.9054972307644688  0.9660414097851315
 0.0000000000000000  0.0660555094618114  0.0390452568815392
 0.0000000000000000  0.9339444905381882  0.0390452568815392
```

N2O

```
  1.00000000000000
   15.00    0.00    0.00
    0.00   15.00    0.00
    0.00    0.00   15.00
  O    N
   1     2
Selective dynamics
Direct
 0.0000000000000000  0.0000000000000000  0.0752575724260098
 0.0000000000000000 -0.0000000000000000  0.9188745093618261
-0.0000000000000000  0.0000000000000000  0.9952812515454955
```

HNO3

```
  1.00000000000000
   15.00    0.00    0.00
    0.00   15.00    0.00
    0.00    0.00   15.00
  O    N    H
   3     1     1
Selective dynamics
Direct
 0.9814652387297124  0.9168489453317040  0.0000000000000000
 0.0791412008859033  0.0314256251762831  0.0000000000000000
 0.9336701091671277  0.0571920364306454  0.0000000000000000
 0.0000039923479045  0.0111655989015977  0.0000000000000000
 0.0418394588693561  0.8911077941597669  0.0000000000000000
```

HNO2

```
  1.00000000000000
   15.00    0.00    0.00
    0.00   15.00    0.00
    0.00    0.00   15.00
  O    N    H
   2     1     1
Selective dynamics
Direct
 0.0599906578101441  0.9586698637192375  0.0000000000000000
 0.9242127342169553  0.0116610788552274  0.0000000000000000
-0.0003442266384329  0.0353461442050247  0.0000000000000000
 0.1184875012780027  0.9880629132205059  0.0000000000000000
```

HNO

```
  1.00000000000000
   15.00    0.00    0.00
    0.00   15.00    0.00
  0.00 0.00   15.00
  H    N    O
   1     1     1
Selective dynamics
Direct
 0.0000000000000000  0.9354015896979062  0.0615607729293427
 0.0000000000000000  0.0039692570436241  0.0391522988457000
 0.0000000000000000  0.0044824865917993  0.9578802615582916
```

NO3

```
  1.00000000000000
   15.00    0.00    0.00
    0.00   15.00    0.00
    0.00    0.00   15.00
  O    N
   3     1
Selective dynamics
Direct
-0.0000000000000000 -0.0000000000000000  0.0838373822041596
 0.0000000000000000  0.0718654046897972  0.9577925621151280
 0.0000000000000000  0.9281345953102026  0.9577925621151280
-0.0000000000000000 -0.0000000000000000 -0.0002371373068913
```

NO2

```
  1.00000000000000
   15.00    0.000    0.00
    0.   15.0999999999999996    0.00
    0.00    0.00   14.9000000000000004
  O    N
   2     1
Selective dynamics
Direct
0.0000000000000000  0.0739312755593464  0.0314902146281935
 0.0000000000000000  0.9260687244406536  0.0314902146281935
-0.0000000000000000  0.0000000000000000 -0.0005240534174670
```

NO

```
  1.00000000000000
   15.00    0.00    0.00
    0.000   15.0999999999999996    0.00
    0.00    0.00   14.9000000000000004
  O    N
   1     1
Selective dynamics
Direct
 0.0000000000000000  0.0000000000000000 -0.0006955860028013
 0.0000000000000000  0.0000000000000000  0.0779304853316587
```

S6.2 Metalloporphyrins for RPBE

Co-porphyrin

```
  1.00000000000000
   20.00   0.00   0.00
    0.00   20.00   0.00
    0.00   0.00   20.00
  Co   C    N    H
   1    20     4    12
Selective dynamics
Direct
 0.0003289907543816 -0.0000106821154876  0.0000000000000000
 0.9994810225298479  0.2111951210027190  0.0341906493615724
-0.0001998714745550  0.1419774681342131  0.0550704820800781
 0.0000263632513188  0.1214589049804935  0.1214671114819427
 0.9995991172690819  0.7888042927149983  0.9658111790989610
 0.0000161917808807  0.8580187965868367  0.9449272635955184
 0.0001153451733967  0.8785366459453323  0.8785234720478859
 0.9995991172690819  0.7888042927149983  0.0341888209010393
 0.0000161917808807  0.8580187965868367  0.0550727364044814
 0.9994810225298479  0.2111951210027190  0.9658093506384280
 0.0001153451733967  0.8785366459453323  0.1214765279521140
 0.0001943708730756  0.0341987502804308  0.7887763258689102
-0.0001998714745550  0.1419774681342131  0.9449295179199215
 0.0003166005168638  0.0550647892105235  0.8579938087438689
 0.0002034946204806  0.9658191862454928  0.7887576009220267
 0.0003476138344332  0.9449275375119780  0.8579772555125339
 0.0000263632513188  0.1214589049804935  0.8785328885180571
 0.0001943708730756  0.0341987502804308  0.2112236741310900
 0.0003166005168638  0.0550647892105235  0.1420061912561306
 0.0002034946204806  0.9658191862454928  0.2112423990779738
 0.0003476138344332  0.9449275375119780  0.1420227444874661
-0.0000575967370942  0.0995457848641531  0.0000000000000000
 0.0002299241772721  0.9004464970612445  0.0000000000000000
 0.0004397791141761 -0.0000095535621429  0.9004170596864242
 0.0004397791141761 -0.0000095535621429  0.0995829403135756
 0.0000129700465526  0.9317813307440848  0.2537091069212618
 0.0000046213099201  0.0682691074590149  0.7463317475592297
 0.0000129700465526  0.9317813307440848  0.7462908930787382
 0.9993744027590582  0.2536397033001029  0.9317430860304236
 0.9993744027590582  0.2536397033001029  0.0682569139695762
 0.0000046213099201  0.0682691074590149  0.2536682524407702
 0.9993362253100371  0.7463529797747495  0.9317526188671680
 0.9993362253100371  0.7463529797747495  0.0682473811328321
-0.0000886827673184  0.1600469861058245  0.8399460504368039
-0.0000886827673184  0.1600469861058245  0.1600539495631959
-0.0000879732445411  0.8399376536603982  0.8399468811922558
-0.0000879732445411  0.8399376536603982  0.1600531188077444
```

Cr-porphyrin

```
  1.00000000000000
   20.00   0.00   0.00
    0.00   20.00   0.00
    0.00   0.00   20.00
  Cr   C    N    H
   1    20     4    12
Selective dynamics
Direct
 0.0002457463998989  0.0000001498036880  0.0000000000000000
-0.0002338693575488  0.2132679032684492  0.0343143929446857
 0.0000054300638757  0.1440415233768396  0.0555604720563511
 0.0000275831751220  0.1221125174175330  0.1221012579197305
 0.9995638508174263  0.7867321642623148  0.9656855408846571
-0.0000873197359287  0.8559587563739099  0.9444404178667276
 0.0000565792067695  0.8778886710844118  0.8778994146576350
 0.9995638508174263  0.7867321642623148  0.0343144591153426
-0.0000873197359287  0.8559587563739099  0.0555595821332718
-0.0002338693575488  0.2132679032684492  0.9656856070553145
 0.0000565792067695  0.8778886710844118  0.1221005853423645
 0.0000907254788852  0.0343167218050852  0.7867347261930099
 0.0000054300638757  0.1440415233768396  0.9444395279436485
 0.0001187067853674  0.0555692394747983  0.8559588673189629
 0.0001816349751739  0.9656864247471295  0.7867355853118567
 0.0002184927570681  0.9444320940014387  0.8559589232641640
 0.0000275831751220  0.1221125174175330  0.8778987420802694
 0.0000907254788852  0.0343167218050852  0.2132652738069902
 0.0001187067853674  0.0555692394747983  0.1440411326810365
 0.0001816349751739  0.9656864247471295  0.2132644146881432
 0.0002184927570681  0.9444320940014387  0.1440410767358359
 0.0001191106846044  0.1024503666766522  0.0000000000000000
 0.0001144539874568  0.8975507920334133  0.0000000000000000
 0.0002162094769538  0.0000012748173932  0.8975435335562203
 0.0002162094769538  0.0000012748173932  0.1024564664437798
 0.0001935947759114  0.9321098640545914  0.2561063267764656
 0.9999641861910132  0.0678923937720638  0.7438958283219606
 0.0001935947759114  0.9321098640545914  0.7438936732235270
-0.0003966570453936  0.2561115134412597  0.9321104110712021
-0.0003966570453936  0.2561115134412597  0.0678895889287981
 0.9999641861910132  0.0678923937720638  0.2561041716780391
 0.9992988778085582  0.7438876078211403  0.9321128417819211
 0.9992988778085582  0.7438876078211403  0.0678871582180859
-0.0000903341187593  0.1607465335610718  0.8392943265561774
-0.0000903341187593  0.1607465335610718  0.1607056734438226
-0.0000250967904966  0.8392546424636935  0.8392939822849953
-0.0000250967904966  0.8392546424636935  0.1607060177150050
```

Fe-porphyrin

```
  1.00000000000000
   20.00   0.00   0.00
    0.00   20.00   0.00
    0.00   0.00   20.00
  Fe   C    N    H
   1    20     4    12
Selective dynamics
Direct
 0.0005476019173455  0.0000004862914236 -0.0000000000000000
 0.9998625060839480  0.2112474786791690  0.0343823858906180
 0.0003656770425454  0.1424506696231057  0.0553038441448844
 0.0005103886998513  0.1214417917358366  0.1218969675057561
 0.9991969486727833  0.7887538192950720  0.9656175543846811
 0.9997293616306804  0.8575503971674565  0.9446962796986336
 0.9998124567457090  0.8785592652182324  0.8781029895182371
 0.9991969486727833  0.7887538192950720  0.0343824456153188
 0.9997293616306804  0.8575503971674565  0.0553037203013662
 0.9998625060839480  0.2112474786791690  0.9656176141093746
 0.9998124567457090  0.8785592652182324  0.1218970104817629
 0.0002433725390113  0.0341248030746757  0.7880146840448928
 0.0003656770425454  0.1424506696231057  0.9446961558551155
 0.0005273675506980  0.0551740524082251  0.8574160642295917
-0.0000281842870836  0.9658764386924767  0.7880151966550626
 0.0001259628280855  0.9448262883667919  0.8574165888097891
 0.0005103886998513  0.1214417917358366  0.8781030324942437
 0.0002433725390113  0.0341248030746757  0.2119853159551076
 0.0005273675506980  0.0551740524082251  0.1425839357704083
-0.0000281842870836  0.9658764386924767  0.2119848033449373
 0.0001259628280855  0.9448262883667919  0.1425834111902109
 0.0005832021610013  0.1001135403245798 -0.0000000000000000
 0.0000519072825784  0.8998871431407964 -0.0000000000000000
 0.0004731397513349 -0.0000001380425252  0.8999141116400220
 0.0004731397513349 -0.0000001380425252  0.1000858883599708
 0.9996324512325021  0.9319405270871158  0.2545304692621674
 0.0000994300389488  0.0680616943041429  0.7454685858163432
 0.9996324512325021  0.9319405270871158  0.7454695307378325
 0.9994071683204011  0.2539355077111695  0.9318612972019298
 0.9994071683204011  0.2539355077111695  0.0681387027980700
 0.0000994300389488  0.0680616943041429  0.2545314141836565
 0.9988451574125573  0.7460655108569979  0.9318602753817905
 0.9988451574125573  0.7460655108569979  0.0681397246182024
 0.0004410511914724  0.1600634419487927  0.8395314557204792
 0.0004410511914724  0.1600634419487927  0.1604685442795139
 0.9995066388660940  0.8399383669948738  0.8395314892774722
 0.9995066388660940  0.8399383669948738  0.1604685107225278
```

Mn-porphyrin

```
  1.00000000000000
   20.00   0.00   0.00
    0.00   20.00   0.00
    0.00   0.00   20.00
  Mn   C    N    H
   1    20     4    12
Selective dynamics
Direct
 0.0007365397189386  0.9999846019958916  0.0000000000000000
 0.9993757282968482  0.2119649514564040  0.0345007352841668
 0.9999569564575274  0.1434240779999847  0.0555478372962065
 0.0001689669514411  0.1217419605182976  0.1222385828767983
 0.9994973002751915  0.7880247877426012  0.9654980190336547
 0.0001639556002374  0.8565644347498148  0.9444459367018433
 0.0002593622678463  0.8782497850677012  0.8777513213632818
 0.9994973002751915  0.7880247877426012  0.0345019809663454
 0.0001639556002374  0.8565644347498148  0.0555540632981567
 0.9993757282968482  0.2119649514564040  0.9654992647158332
 0.0002593622678463  0.8782497850677012  0.1222486786367182
 0.0000789307943495  0.0341299818301164  0.7877579047555308
 0.9999569564575274  0.1434240779999847  0.9444521627037935
 0.0004038702662614  0.0555818748784023  0.8570653209494721
 0.0000913239232103  0.9658968230279723  0.7877428347348906
 0.0004392948471278  0.9444113436956909  0.8570471379995952
 0.0001689669514411  0.1217419605182976  0.8777614171232018
 0.0000789307943495  0.0341299818301164  0.2122420952444692
 0.0004038702662614  0.0555818748784023  0.1429346790505279
 0.0000913239232103  0.9658968230279723  0.2122571652651093
 0.0004392948471278  0.9444113436956909  0.1429528620004048
 0.0002434662680555  0.1013762584492793  0.0000000000000000
 0.0005282837136816  0.8986069485678012  0.0000000000000000
 0.0006523573551848  0.9999909847559928  0.8994508384722294
 0.0006523573551848  0.9999909847559928  0.1005491615277706
 0.9998119970139627  0.9320879207265957  0.2549013019750220
 0.9997734411724319  0.0679823340018340  0.7451475248336087
 0.9998119970139627  0.9320879207265957  0.7450986980249781
 0.9990216392765020  0.2547265172458563  0.9318401094423854
 0.9990216392765020  0.2547265172458563  0.0681598905576145
 0.9997734411724319  0.0679823340018340  0.2548524751663912
 0.9990116460008027  0.7452566206105488  0.9318508817319230
 0.9990116460008027  0.7452566206105488  0.0681491182680770
 0.9999631958260627  0.1602059432452107  0.8390356843464393
 0.9999631958260627  0.1602059432452107  0.1609643156535679
 0.9999181388246691  0.8397862539405098  0.8390258310926191
 0.9999181388246691  0.8397862539405098  0.1609741689073809
```

Ti-porphyrin

```
  1.00000000000000
   20.00   0.00   0.00
    0.00   20.00   0.00
    0.00   0.00   20.00
  Ti   C    N    H
   1    20     4    12
Selective dynamics
Direct
 0.0011850339159051  0.0000456650493864 -0.0000000000000000
 0.9987209258490597  0.2141682655313497  0.0345663152474446
 0.0007236636096287  0.1455106008582882  0.0561606006267684
 0.0010200129937012  0.1225947273660238  0.1229369822269481
 0.0003078954151126  0.7858051420148195  0.9654342621894390
-0.0003648436171644  0.8544972504055290  0.9438468695732046
-0.0005843498531198  0.8774262643186940  0.8770674120340612
 0.0003078954151126  0.7858051420148195  0.0345657378105611
-0.0003648436171644  0.8544972504055290  0.0561531304267952
 0.9987209258490597  0.2141682655313497  0.9654336847525558
-0.0005843498531198  0.8774262643186940  0.1229325879659393
 0.0007500153320506  0.0343461377360894  0.7854698710362291
 0.0007236636096287  0.1455106008582882  0.9438393993732316
 0.0013069761228350  0.0561995934458992  0.8545804341051760
 0.9991994217050463  0.9656605300169857  0.7854837582616687
-0.0006968804760873  0.9438135926714873  0.8545892970417888
 0.0010200129937012  0.1225947273660238  0.8770630177730524
 0.0007500153320506  0.0343461377360894  0.2145301289637704
 0.0013069761228350  0.0561995934458992  0.1454195658948240
 0.9991994217050463  0.9656605300169857  0.2145162417383308
-0.0006968804760873  0.9438135926714873  0.1454107029582110
 0.0023197234936972  0.1042309175453847 -0.0000000000000000
 0.9989068634625434  0.8958042134608674 -0.0000000000000000
 0.0005444086973417  0.0000030317230761  0.8960241563083990
 0.0005444086973417  0.0000030317230761  0.1039758436916006
 0.9981868761865993  0.9323756588588917  0.2575829308521285
 0.0011896695244680  0.0675987066416025  0.7423742571872693
 0.9981868761865993  0.9323756588588917  0.7424170691478716
 0.9973226504993185  0.2572886339786692  0.9322449266069296
 0.9973226504993185  0.2572886339786692  0.0677550733930700
 0.0011896695244680  0.0675987066416025  0.2576257428127309
 0.0005153869391584  0.7426481282301004  0.9322531338846957
 0.0005153869391584  0.7426481282301004  0.0677468661153045
 0.0004400431234965  0.1612066426526707  0.8384218094573656
 0.0004400431234965  0.1612066426526707  0.1615781905426346
-0.0004454324875303  0.8388271955220100  0.8384101595799678
-0.0004454324875303  0.8388271955220100  0.1615898404200322
```

V-porphyrin

```
  1.00000000000000
   20.00   0.00   0.00
    0.00   20.00   0.00
    0.00   0.00   20.00
  V    C    N    H
   1    20     4    12
```

```
Selective dynamics
Direct
  0.0016319257413545  0.0000098844853083 -0.0000000000000000
  0.9993280931276567  0.2129565722605226  0.0345914561964350
  0.0001605767588074  0.1443319241148569  0.0558651023592187
  0.0002263127869538  0.1220431204886917  0.1227595412954018
  0.9994240567839561  0.7870460698083206  0.9654052925037860
  0.0002514078667804  0.8556657366112230  0.9441242207743632
  0.0002630061179158  0.8779641089509169  0.8772281178403948
  0.9994240567839561  0.7870460698083206  0.0345947074962137
  0.0002514078667804  0.8556657366112230  0.0558757792256367
  0.9993280931276567  0.2129565722605226  0.9654085438035650
  0.0002630061179158  0.8779641089509169  0.1227718821596055
  0.9999647199959009  0.0342481507376672  0.7856980515937253
  0.0001605767588074  0.1443319241148569  0.9441348976407817
  0.0005558574063375  0.0557219139148078  0.8551347051398528
  0.9999801734710113  0.9657468646727648  0.7856969531684185
  0.0005793285859397  0.9442752120914635  0.8551228952248755
  0.0002263127869538  0.1220431204886917  0.8772404587045983
  0.9999647199959009  0.0342481507376672  0.2143019484062747
  0.0005558574063375  0.0557219139148078  0.1448652948601469
  0.9999801734710113  0.9657468646727648  0.2143030468315816
  0.0005793285859397  0.9442752120914635  0.1448771047751246
  0.0006672334959259  0.1027357517907249 -0.0000000000000000
  0.0007730637102282  0.8972524518678413 -0.0000000000000000
  0.0010386301204331 -0.0000076343074156  0.8967830088300512
  0.0010386301204331 -0.0000076343074156  0.1032169911699487
  0.9994622054446306  0.9322566181981267  0.2572134295157394
  0.9994525107486987  0.0677687407311127  0.7427974505881250
  0.9994622054446306  0.9322566181981267  0.7427865704842603
  0.9987156824576300  0.2559985447821273  0.9320904689436744
  0.9987156824576300  0.2559985447821273  0.0679095310563255
  0.9994525107486987  0.0677687407311127  0.2572025494118749
  0.9987882487260100  0.7439978825732788  0.9320902552179009
  0.9987882487260100  0.7439978825732788  0.0679097447820990
 -0.0002002519047927  0.1607967808282433  0.8387386107875976
 -0.0002002519047927  0.1607967808282433  0.1612613892124027
 -0.0001844199676364  0.8392008494713594  0.8387417756366687
 -0.0001844199676364  0.8392008494713594  0.1612582243633309
```

Co-porphyrin*NO

```
   1.00000000000000
   20.00   0.00   0.00
    0.00   20.00   0.00
    0.00   0.00   20.00
   Co   C    N    H    O
    1    20     5    12     1
Selective dynamics
Direct
  0.0128492186523869 -0.0008903878223419 -0.0000000000000000
  0.0013899033113488  0.2104822217174566  0.0341755719078552
  0.0029885424746709  0.1411837059167124  0.0550211241501534
  0.0029822824221596  0.1205872997149022  0.1214513918880318
  0.9980082202396686  0.7872183794923703  0.9658164217590459
  0.0006096155914250  0.8565258390336780  0.9449843014298326
  0.0008971799646800  0.8773906804083623  0.8786285217094124
  0.9980082202396686  0.7872183794923703  0.0341835782409536
  0.0006096155914250  0.8565258390336780  0.0550156985701676
  0.0013899033113488  0.2104822217174566  0.9658244280921452
  0.0008971799646800  0.8773906804083623  0.1213714782905877
  0.0003174887972120  0.0332090047585123  0.7887497176214325
  0.0029885424746709  0.1411837059167124  0.9449788758498460
  0.0021367304207136  0.0541738185481227  0.8578839821820355
  0.9996175193968176  0.9647848355214917  0.7888866882159697
  0.0010987942663634  0.9439120700581263  0.8580931767359331
  0.0029822824221596  0.1205872997149022  0.8785486081119684
  0.0003174887972120  0.0332090047585123  0.2112502823785670
  0.0021367304207136  0.0541738185481227  0.1421160178179640
  0.9996175193968176  0.9647848355214917  0.2111133117840307
  0.0010987942663634  0.9439120700581263  0.1419068232640668
  0.0037999778575057  0.0990295749403779 -0.0000000000000000
  0.0019783839815871  0.8985591147250809 -0.0000000000000000
  0.0021945981276495 -0.0008927601480392  0.9001050601234714
  0.0021945981276495 -0.0008927601480392  0.0998949398765286
  0.1036239257015601  0.9939259675287601 -0.0000000000000000
  0.9978745655038168  0.9307205781439238  0.2535130162707381
  0.9994131414413062  0.0671698087656161  0.7462421498423417
  0.9978745655038168  0.9307205781439238  0.7464869837292621
  0.0000082169733837  0.2528881331614328  0.9317552118073898
  0.0000082169733837  0.2528881331614328  0.0682447881926096
  0.9994131414413062  0.0671698087656161  0.2537578501576578
  0.9959781985106949  0.7448107069493911  0.9317682862511625
  0.9959781985106949  0.7448107069493911  0.0682317137488376
  0.0026913937112667  0.1591913157206166  0.8399783191383625
  0.0026913937112667  0.1591913157206166  0.1600216808616375
 -0.0000031620097676  0.8389706865218000  0.8398880149169061
 -0.0000031620097676  0.8389706865218000  0.1601119850830938
  0.1397855355201117  0.0413400820591821 -0.0000000000000000
```

Cr-porphyrin*NO

```
   1.00000000000000
   20.00   0.00   0.00
    0.00   20.00   0.00
    0.00   0.00   20.00
   Cr   C    N    H    O
    1    20     5    12     1
Selective dynamics
Direct
  0.0191206273521284  0.9999103707349416  0.0000000000000000
  0.9987428911624816  0.2125733073854283  0.0342570577756803
  0.0011342025403736  0.1433618277784142  0.0554061428274522
  0.0012064715043554  0.1217696865760422  0.1218592671147588
  0.9987221676735984  0.7872584155454387  0.9657425814723567
  0.0011019195583318  0.8564697651284412  0.9445933547309274
  0.0011715388314070  0.8780630232185569  0.8781404841303109
  0.9987221676735984  0.7872584155454387  0.0342574185276433
  0.0011019195583318  0.8564697651284412  0.0554066452690727
  0.9987428911624816  0.2125733073854283  0.9657429422243197
  0.0011715388314070  0.8780630232185569  0.1218595158696890
  0.9992518416874334  0.0341739687322406  0.7873113999330911
  0.0011342025403736  0.1433618277784142  0.9445938571725481
  0.0013455029776666  0.0553220117925798  0.8565338818340149
  0.9992340050684373  0.9656600703796838  0.7873118240859750
  0.0013226810948824  0.9445109570156170  0.8565336031274499
  0.0012064715043554  0.1217696865760422  0.8781407328852412
  0.9992518416874334  0.0341739687322406  0.2126886000669090
  0.0013455029776666  0.0553220117925798  0.1434661181659923
  0.9992340050684373  0.9656600703796838  0.2126881759140252
  0.0013226810948824  0.9445109570156170  0.1434663968725503
  0.0023331042574899  0.1015885568258273  0.0000000000000000
  0.0022968605109074  0.8982418874327415  0.0000000000000000
  0.0024039676390260  0.9999157313226312  0.8983080984546358
  0.0024039676390260  0.9999157313226312  0.1016919015453642
  0.1025361761576968  0.9998767666039710  0.0000000000000000
  0.9974991039008339  0.9318891124180825  0.2553364483105537
  0.9975398130315991  0.0679441135125428  0.7446614853128364
  0.9974991039008339  0.9318891124180825  0.7446635516894464
  0.9968190546963493  0.2552127293653297  0.9319713008911772
  0.9968190546963493  0.2552127293653297  0.0680286991088230
  0.9975398130315991  0.0679441135125428  0.2553385146871637
  0.9968100377145043  0.7446189259120213  0.9319707980685462
  0.9968100377145043  0.7446189259120213  0.0680292019314541
  0.0002024852607418  0.1603775460793854  0.8395405146986276
  0.0002024852607418  0.1603775460793854  0.1604594853013725
  0.0001579709328670  0.8394552942236718  0.8395405513786259
  0.0001579709328670  0.8394552942236718  0.1604594486213742
  0.1628254211719864  0.9998464456302764  0.0000000000000000
```

Fe-porphyrin*NO

```
   1.00000000000000
   20.00   0.00   0.00
    0.00   20.00   0.00
    0.00   0.00   20.00
   Fe   C    N    H    O
    1    20     5    12     1
Selective dynamics
Direct
  0.0176270376115350  0.9994212922143568  0.0000000000000000
  0.0014849097310376  0.2102901875305442  0.0342310633086711
  0.0031647463391185  0.1411380898426102  0.0552239508930018
  0.0027955326911213  0.1203296544454619  0.1217103721360095
  0.9973767519124916  0.7867133804630739  0.9657733766565187
  0.0002909753347188  0.8559947547107216  0.9449338708104468
  0.0005245250407476  0.8771193260065994  0.8784807507283537
  0.9973767519124916  0.7867133804630739  0.0342266233434812
  0.0002909753347188  0.8559947547107216  0.0550661291895534
  0.0014849097310376  0.2102901875305442  0.9657689366913287
  0.0005245250407476  0.8771193260065994  0.1215192492716462
  0.9994020262378575  0.0328575533793493  0.7880708842918779
  0.0031647463391185  0.1411380898426102  0.9447760491069982
  0.0018224800419680  0.0538920027355642  0.8572717698648293
  0.9987915828000237  0.9644189152461499  0.7882651311249627
  0.0008457777429798  0.9435985240973476  0.8575620034840532
  0.0027955326911213  0.1203296544454619  0.8782896278639907
  0.9994020262378575  0.0328575533793493  0.2119291157081223
  0.0018224800419680  0.0538920027355642  0.1427282301351707
  0.9987915828000237  0.9644189152461499  0.2117348688750372
  0.0008457777429798  0.9435985240973476  0.1424379965159466
  0.0039279057540125  0.0990433102551529  0.0000000000000000
  0.0019486290248803  0.8978890032293086  0.0000000000000000
  0.0023762702822449  0.9988843499135960  0.8993922405821257
  0.0023762702822449  0.9988843499135960  0.1006077594178741
  0.1031137166885116  0.0051542488698778  0.0000000000000000
  0.9967478932277677  0.9303909776341214  0.2541528978895639
  0.9980298281207732  0.0667222236920321  0.7454911877741265
  0.9967478932277677  0.9303909776341214  0.7458471021104364
  0.9999734198047029  0.2528001036791244  0.9318300559183545
  0.9999734198047029  0.2528001036791244  0.0681699440816454
  0.9980298281207732  0.0667222236920321  0.2545088122258735
  0.9951067271988010  0.7442286693760132  0.9318329967727699
  0.9951067271988010  0.7442286693760132  0.0681670032272302
  0.0023098994834062  0.1590215525609597  0.8397999772394641
  0.0023098994834062  0.1590215525609597  0.1602000227605359
  0.9994379521316709  0.8386230374718897  0.8397879305991686
  0.9994379521316709  0.8386230374718897  0.1602120694008313
  0.1508636146781420  0.0410625398609645  0.0000000000000000
```

Mn-porphyrin*NO

```
   1.00000000000000
   20.00   0.00   0.00
    0.00   20.00   0.00
    0.00   0.00   20.00
   Mn   C    N    H    O
    1    20     5    12     1
Selective dynamics
Direct
  0.0181948146303155  0.9999015625104288 -0.0000000000000000
  0.9990488384820454  0.2119250372566524  0.0342315014911573
  0.0009924155424324  0.1426823379835110  0.0551792922107734
  0.0011717136121396  0.1215412907098187  0.1216158515636787
  0.9990359886019925  0.7879412942093111  0.9657677243550026
  0.0009504849550698  0.8571826411440205  0.9448178099299683
  0.0011663307014773  0.8783283974059929  0.8783821990941464
  0.9990359886019925  0.7879412942093111  0.0342322756449974
  0.0009504849550698  0.8571826411440205  0.0551821900700318
  0.9990488384820454  0.2119250372566524  0.9657684985088426
  0.0011663307014773  0.8783283974059929  0.1216178009058535
  0.9996167211233900  0.0341709557003818  0.7879642205148503
  0.0009924155424324  0.1426823379835110  0.9448207077892263
  0.0012305391411005  0.0551152518102331  0.8572127572003848
  0.9996300306474707  0.9657045733562201  0.7879614010969884
  0.0012400521195600  0.9447558372370191  0.8572101486486049
  0.0011717136121396  0.1215412907098187  0.8783841484363210
  0.9996167211233900  0.0341709557003818  0.2120357794851498
  0.0012305391411005  0.0551152518102331  0.1427872427996158
  0.9996300306474707  0.9657045733562201  0.2120385989030113
  0.0012400521195600  0.9447558372370191  0.1427898513513953
  0.0017955281429337  0.1005194156121742 -0.0000000000000000
  0.0017177214913000  0.8993437625041572 -0.0000000000000000
  0.0018890907251888 -0.0000661064706729  0.8993796964624818
  0.0018890907251888 -0.0000661064706729  0.1006203035375178
  0.0995326325067948  0.9996746433398933 -0.0000000000000000
  0.9983240560182121  0.9318491285355659  0.2546368133841069
  0.9982929822426223  0.0680338678159621  0.7453719585638749
  0.9983240560182121  0.9318491285355659  0.7453631866158936
  0.9975226713993631  0.2545062602331641  0.9319029487732793
  0.9975226713993631  0.2545062602331641  0.0680970512267209
  0.9982929822426223  0.0680338678159621  0.2546280414361248
  0.9975531220535138  0.7453560583688774  0.9319045144587197
  0.9975531220535138  0.7453560583688774  0.0680954855412804
  0.0006002633501938  0.1601633022024340  0.8397841535323246
  0.0006002633501938  0.1601633022024340  0.1602158464676754
  0.0006157455437211  0.8397080565147930  0.8397790958296618
  0.0006157455437211  0.8397080565147930  0.1602209041703380
  0.1594407107096432  0.9993812480067871 -0.0000000000000000
```

Ti-porphyrin*NO

```
   1.00000000000000
   20.00   0.00   0.00
    0.00   20.00   0.00
    0.00   0.00   20.00
   Ti   C    N    H    O
    1    20     5    12     1
Selective dynamics
Direct
  0.0257150080204603  0.9999107212046860  0.0000000000000000
  0.9979978248131126  0.2129213966919160  0.0344308649462801
  0.0003946329351346  0.1440416724053549  0.0558792727859382
  0.0001932126179554  0.1219017849721332  0.1226752459052120
  0.9979827712930737  0.7869379099840931  0.9655692043398949
  0.0003197410292218  0.8558193357992619  0.9441193823800563
  0.0001283343668756  0.8779575714710754  0.8773220174408083
  0.9979827712930737  0.7869379099840931  0.0344307956601051
  0.0003197410292218  0.8558193357992619  0.0558806176199409
  0.9979978248131126  0.2129213966919160  0.9655691350537180
  0.0001283343668756  0.8779575714710754  0.1226779825591892
  0.9973891876366241  0.0341896666146811  0.7853930466522735
  0.0003946329351346  0.1440416724053549  0.9441207272140657
  1.0010706760169172  0.0556358647731676  0.8547266774188891
  0.9973657233236123  0.9656759543398326  0.7853903772157278
  0.0010341774107885  0.9442225802506747  0.8547226132960416
  0.0001932126179554  0.1219017849721332  0.8773247540947905
  0.9973891876366241  0.0341896666146811  0.2146069533477282
  1.0010706760169172  0.0556358647731676  0.1452733225811130
  0.9973657233236123  0.9656759543398326  0.2146096227842700
  0.0010341774107885  0.9442225802506747  0.1452773867039559
  0.0018864663767053  0.1026831344513819  0.0000000000000000
  1.0017699208099500  0.8971773108134420  0.0000000000000000
  1.0042190520759342 -0.0000735979362427  0.8959343156166878
  1.0042190520759342 -0.0000735979362427  0.1040656843833121
  0.1179621140592882  0.9996958613513132  0.0000000000000000
  0.9945774203597779  0.9321606698865215  0.2574047315540673
  0.9946263278751506  0.0677112824872102  0.7426011299982495
  0.9945774203597779  0.9321606698865215  0.7425952684459339
  0.9963551225137985  0.2558595988381941  0.9321638076934253
  0.9963551225137985  0.2558595988381941  0.0678361923065752
  0.9946263278751506  0.0677112824872102  0.2573988700017509
  0.9963945254360915  0.7439980237334938  0.9321633308617010
  0.9963945254360915  0.7439980237334938  0.0678366691383040
 -0.0018860741496824  0.1607720657503707  0.8390045158669366
 -0.0018860741496824  0.1607720657503707  0.1609954841330654
 -0.0019439765036817  0.8390866672288603  0.8390022703501359
 -0.0019439765036817  0.8390866672288603  0.1609977296498620
  0.1786726326321420  0.9995130775979836  0.0000000000000000
```

V-porphyrin*NO

```
   1.00000000000000
   20.00   0.00   0.00
    0.00   20.00   0.00
    0.00   0.00   20.00
   V    C    N    H    O
    1    20     5    12     1
Selective dynamics
Direct
  0.0202905567846675  0.9999067199019509  0.0000000000000000
  0.9983333997268166  0.2131026433686728  0.0342696035703359
  0.0011889089539626  0.1439398807228910  0.0556169590137305
  0.0011656468542586  0.1219821816269407  0.1220710485743302
  0.9983589593355253  0.7867086499436383  0.9657289224677876
  0.0012507472706879  0.8558676241695399  0.9443799091759605
  0.0011273546045156  0.8778279703346251  0.8779252112791505
  0.9983589593355253  0.7867086499436383  0.0342710775322124
  0.0012507472706879  0.8558676241695399  0.0556200908240390
  0.9983333997268166  0.2131026433686728  0.9657303964296641
  0.0011273546045156  0.8778279703346251  0.1220747887208496
  0.9989787467784169  0.0341953529291448  0.7867527632282270
  0.0011889089539626  0.1439398807228910  0.9443830409862697
  0.0015416162733217  0.0555365816703300  0.8559103571327438
  0.9989283480749155  0.9656293091802802  0.7867493063524255
  0.0014945076977320  0.9442728862521843  0.8559023237199350
  0.0011656468542586  0.1219821816269407  0.8779289514256700
  0.9989787467784169  0.0341953529291448  0.2132472367717725
  0.0015416162733217  0.0555365816703300  0.1440896428672558
  0.9989283480749155  0.9656293091802802  0.2132506936475748
  0.0014945076977320  0.9442728862521843  0.1440976762800654
  0.0028896375331558  0.1023984820394498  0.0000000000000000
  0.0029989932558350  0.8974059061421686  0.0000000000000000
  0.0030962910493026 -0.0000987549092725  0.8974373948258965
  0.0030962910493026 -0.0000987549092725  0.1025626051741036
  0.1053350612076646  0.9999822322045323  0.0000000000000000
  0.9967595609007234  0.9320038768466046  0.2559805328751731
  0.9968812188518039  0.0678365293350111  0.7440292601570624
  0.9967595609007234  0.9320038768466046  0.7440194671248268
  0.9960938592719328  0.2558082363300015  0.9320744890425346
  0.9960938592719328  0.2558082363300015  0.0679255109574655
  0.9968812188518039  0.0678365293350111  0.2559707398429378
  0.9960600769221202  0.7440037847130804  0.9320796840291137
  0.9960600769221202  0.7440037847130804  0.0679203159708863
 -0.0002904224604110  0.1605884275430665  0.8393312340425548
 -0.0002904224604110  0.1605884275430665  0.1606687659574450
 -0.0004416080989937  0.8392158069177417  0.8393411124104586
 -0.0004416080989937  0.8392158069177417  0.1606588875895413
  0.1658748272053896  0.0000816857629484  0.0000000000000000
```

Co-porphyrin*NO2

```
   1.00000000000000
   20.00   0.00   0.00
    0.00   20.00   0.00
    0.00   0.00   20.00
   Co   C    N    H    O
    1    20     5    12     2
Selective dynamics
Direct
  0.0098861392594063  0.0016285160900753  0.9998220259797015
  0.9966825147687686  0.2127646126922178  0.0346227898235645
  0.9987668028951737  0.1436120182437660  0.0555210908890266
 -0.0004308771648163  0.1230363520245502  0.1218738651924945
  0.9968305620109286  0.7905329977947162  0.9650810102675749
  0.9986210713118839  0.8596935038699570  0.9441648338378091
 -0.0008903322403378  0.8802768906434558  0.8778041231455322
  0.9978845252204862  0.7905717908791392  0.0334352868406112
  0.0001815025800124  0.8597608669742062  0.0542369478047016
  0.9975499209966351  0.2127235817440776  0.9662616642231449
  0.0018641546367917  0.8802222275913906  0.1206024052460478
  0.0014182643631639  0.0363908332482135  0.7889234977818405
  0.0000393658995421  0.1435402037925747  0.9454659288128223
  0.0019228798379435  0.0568175277696952  0.8582507820497792
  0.0002773096621188  0.9680347637371461  0.7884892611132638
  0.0002905346791432  0.9467324066546687  0.8575587820998264
  0.0017545278992288  0.1230811490914270  0.8791008297652430
```

0.0007934180599416 0.0353009304605898 0.2112009966793808
0.0007749590457593 0.0565862779909738 0.1421250505112352
0.0016641483536803 0.9669404081113666 0.2107722726723026
0.0020693979803593 0.9464918881853424 0.1414488484747677
0.9999307064064319 0.1012380433780860 0.0005035078937177
0.9998690260083257 0.9020445215708941 0.9991914841778951
0.0008941474516150 0.0015509258411372 0.9002286042150245
0.0011593831278216 0.0017615029845948 0.0994625698154724
0.1050129920863745 0.0015342257700099 -0.0003968348376537
0.0016089590649828 0.9325842056202795 0.2529665549766288
0.0015211447361324 0.0707231736499521 0.7467146647249530
-0.0007188816556677 0.9342168233479734 0.7458639528867291
0.9963490281203198 0.2550997236509460 0.9321466227299486
0.9946862859485025 0.2551736644773095 0.0686637222797023
-0.0000244236682706 0.0690943054302116 0.2538436018739415
0.9948864462563694 0.7481236698747790 0.9310482813346306
0.9969657867190836 0.7482107319669894 0.0675700172008841
0.0022077362063838 0.1617864487191804 0.8406417695587146
-0.0010414507648810 0.1615168706940900 0.1605492642322633
-0.0016213023078077 0.8418059123343188 0.8391169015422855
0.0022976567474031 0.8415389266927464 0.1590779061445931
0.1331658144614866 0.0423751426401741 0.9625022529225774
0.1331846549995377 0.9606024337667906 0.0365928631169827

Fe-porphyrin*NO2
1.00000000000000
20.00 0.00 0.00
0.00 20.00 0.00
0.00 0.00 20.00
Fe C N H O
1 20 5 12 2
Selective dynamics
Direct
0.0138065099442817 0.9998875008914120 0.0000756986577782
0.9890488689118764 0.2098299488622627 0.0343883919231082
-0.0058342050326117 0.1410656723644870 0.0553001444994087
-0.0000314149163955 0.1209323221302817 0.1217430603645614
0.9891097749547835 0.7899295397564626 0.9657103824042467
-0.0056945699624305 0.8586913547457878 0.9448090859078343
0.0002094877433171 0.8788219298278042 0.8783758471433983
0.9891402674707206 0.7899342535629262 0.0342892786368479
-0.0056418126177718 0.8587011879779032 0.0551871451682676
0.9892448806062478 0.2098271844287738 0.9658065321684559
0.0002890125744325 0.8788260814860491 0.1216168883707468
0.0082148348055851 0.0340867176974659 0.7881012344898283
-0.0055352566300213 0.1410590205866112 0.9449145775500430
0.0064571687557260 0.0549558493281845 0.8574349657473047
0.0080685586958965 0.9657431661483141 0.7880560403808369
0.0062390676780415 0.9448019319940181 0.8573654667369811
0.0005464984146972 0.1209294737396286 0.8784945714094815
0.0076813860245282 0.0340110199737154 0.2120754604206603
0.0059783202482244 0.0549588393917993 0.1427649395378772
0.0078156181303375 0.9656666862692779 0.2120266847832425
0.0061799122756364 0.9448017275843406 0.1426891735627476
-0.0044732249442665 0.0986985016646684 0.0001069235545450
0.9955768572100114 0.9010608510875225 0.0000009608311992
0.0067383861972009 -0.0001436344903042 0.8996867669364222
0.0065657655294845 -0.0000984228696608 0.1004416659931327
0.1090375870170020 -0.0000003427989921 0.0001081371431537
0.0077645447957794 0.9316498610758600 0.2544988455752074
0.0082371011522433 0.0680972906334083 0.7456284820575657
0.0079562395442265 0.9317750774962748 0.7455422525778304
0.9869172504039327 0.2523078623416948 0.9318768109437667
0.9865364389132582 0.2523101970221159 0.0682976523023266
0.0074935312492725 0.0679707120279852 0.2545905380730630
0.9866673047366887 0.7474450052202500 0.9317957948050077
0.9867343102784791 0.7474536760945933 0.0682106009846807
-0.0000589624901549 0.1598570759170864 0.8402388074570942
-0.0008648645533973 0.1598363750964336 0.1600170077736479
-0.0005321566958681 0.8399139653000012 0.8401030242479711
-0.0004143290086890 0.8399018163227633 0.1598745390745284
0.1374067743917270 0.0552381471955511 -0.0020521933532594
0.1374530381979495 0.9447855769152548 0.0023078131584571

Cr-porphyrin*NO2
1.00000000000000
20.00 0.00 0.00
0.00 20.00 0.00
0.00 0.00 20.00
Cr C N H O
1 20 5 12 2
Selective dynamics
Direct
0.0136286166155656 0.9998906134504681 0.0000532638605017
0.9967490370373886 0.2127728011980250 0.0343173787088577
-0.0017321571951485 0.1435612424976004 0.0554465291156458
-0.0004474569981419 0.1219110994357989 0.1219259592088438
0.9967196113601862 0.7869921460053331 0.9657629996254719
-0.0017455897741235 0.8561979236787053 0.9446350614781202
-0.0004427875688622 0.8778523859963897 0.8781582640514997
0.9966869033317661 0.7869931345048586 0.0343215674692595
-0.0017955360968474 0.8561992699158946 0.0554492044568171
0.9967808800730226 0.2127732363294955 0.9657646573985303
-0.0005346909710996 0.8778537211572071 0.1219264707005224
0.9992878667254934 0.0341881703353329 0.7874088962932906
-0.0016834251780908 0.1435616947175326 0.9446362557276470
0.0008268533868203 0.0554208018676245 0.8564869213923350
0.9992486003710995 0.9655620101357388 0.7874130983339762
0.0007760557940986 0.9443371595518741 0.8564904230408178
-0.0003577636923564 0.1219106379368799 0.8781575942094619
0.9991983339736171 0.0341896494420729 0.2126803704606563
0.0007349887052284 0.0554222900889281 0.1436016280244562
0.9991580283414028 0.9655632582659467 0.2126765214373255
0.0006828057728642 0.9443376254216651 0.1435986672835569
-0.0013704556633371 0.1019164970261676 0.0000411836577194
0.9985836461641299 0.8978460231182539 0.0000415871805215
0.0020407148674628 -0.0001234995239416 0.8982018202988197
0.0019498413920332 -0.0001227273808606 0.1018901330479430
0.1159517260957232 0.9999835738836417 0.0002562213391191
0.9977325265358147 0.9318974749435915 0.2554129516156949
0.9979068447503645 0.0678562573191955 0.7446696375576013
0.9978129444301265 0.9318955805418129 0.7446776954305566
0.9960019129136093 0.2554944237048001 0.9320602871228330
0.9959381183023273 0.2554957717486321 0.0680187915349956
0.9978286427005190 0.0678576238923318 0.2554202195124686
0.9959441679646093 0.7442641408225732 0.9320617076394151
0.9958785219887474 0.7442638500284033 0.0680205069818705
-0.0009057357674590 0.1604757081200698 0.8395143415931008
-0.0010159574178082 0.1604784797156485 0.1605657990994475
-0.0010336131403902 0.8392856956017114 0.8395210674958503
-0.0011469771516710 0.8392852408589165 0.1605607495327167
0.1450871642216884 0.0551003921320318 0.0003122254653942
0.1451112927996098 0.9448796215136579 0.0003413416163361

Mn-porphyrin*NO2
1.00000000000000
20.00 0.00 0.00
0.00 20.00 0.00
0.00 0.00 20.00
Mn C N H O
1 20 5 12 2
Selective dynamics
Direct
0.0141337517580195 0.0008448245053857 0.0001468330317468
0.9970351643575300 0.2130687008227836 0.0343691223683890
-0.0008365687420396 0.1439573328848302 0.0555230223917066
-0.0009595221753226 0.1227343830574211 0.1220213555822388
0.0006241058322344 0.7886854111291060 0.9658671975581476
0.0017078318920710 0.8577851891222594 0.9447149894086417
0.0015296706184644 0.8789515912851863 0.8782139154199003
0.0005662779181515 0.7886879631774399 0.0343921896318386
0.0016019113455685 0.8577907886384929 0.0555427341209953
0.9970693492991671 0.2130650259322155 0.9658423146183013
0.0012379982107641 0.8789710982319546 0.1220413255347694
0.9983543220277850 0.0350972060318072 0.7877601834078477
-0.0007696712067937 0.1439539283746924 0.9446995128297710
0.9997194832680997 0.0562513277908284 0.8568567598319162
0.9989884070178308 0.9665425754016318 0.7877526157492557
0.0007318559121916 0.9454237552786082 0.8568453421683617
-0.0007351524291633 0.1227228785572844 0.8782063824538902
0.9978998038627616 0.0351248761846580 0.2124845299597071
0.9993732742013772 0.0562658826391271 0.1433858959375204
0.9985035027294833 0.9665692429217303 0.2124961726109175
0.0003395314271125 0.9454408214998640 0.1434119282332295
0.0004703093290126 0.1023575093136532 0.0001137777959536
0.0020509652576118 0.8994441818182122 0.0001258612128385
0.0009645449796323 0.0008245576526117 0.8984532031303170
0.0006196855990830 0.0008374312522571 0.1018042705987627
0.1584917969787414 0.9522501486112627 -0.0012836805188248
0.9976707382573589 0.9327789566670077 0.2551304971430132
0.9969706111068586 0.0688781512873806 0.7451322216098706
0.9982730107182477 0.9327545891328887 0.7451159237688352
0.9955429036812293 0.2556806582762747 0.9320574040604799
0.9954376033883463 0.2556886240555616 0.0681423796426049
0.9964260817095444 0.0689137652775278 0.2550978847350034
0.9998897779497664 0.7460681362942412 0.9320569264532176
0.9997333774581304 0.7460717879765382 0.0681993426195252
-0.0016509325253150 0.1613752069480374 0.8396858685508768
-0.0019421904082995 0.1613889408001151 0.1605321608157191
0.0014669497791001 0.8402985456936201 0.8396889064824232
0.0010679900684911 0.8403252295420562 0.1605726438424020
0.1157864384214919 0.0052357785999485 0.0006598247033192
0.2176995111256544 0.9681139973335042 -0.0006097394954318

Ti-porphyrin*NO2
1.00000000000000
20.00 0.00 0.00
0.00 20.00 0.00
0.00 0.00 20.00
Ti C N H O
1 20 5 12 2
Selective dynamics
Direct
0.0352024405027494 -0.0006596576175249 -0.0001897122901169
0.9928917687079069 0.2131936135020556 0.0341019660968311
-0.0022824979478031 0.1440276234983625 0.0551740205316867
-0.0015297196652774 0.1225090362680668 0.1218484265171832
0.9943323225058028 0.7867377124899285 0.9655091148385446
-0.0007525212454619 0.8558467702906959 0.9444024978428585
0.0000263481583304 0.8782911599889416 0.8780431565765597
0.9943747815618381 0.7867353136429559 0.0341604883321169
-0.0006829228925732 0.8558480713513206 0.0552540709875951
0.9928503905693602 0.2131899363180572 0.9655609038456504
0.0001776462822622 0.8782942877343983 0.1216168401517979
0.9963071656973150 0.0345628577242342 0.7869433465720304
-0.0023519290918161 0.1440265707377916 0.9444778031856235
0.0003040624909815 0.0560953026938658 0.8558409304944841
0.9971650159934119 0.9659178848416773 0.7872798795564587
0.0014508096826601 0.9449589449314526 0.8564001099358264
-0.0016847439699131 0.1225089563779816 0.8778060595193877
0.9965820089578413 0.0345674061148336 0.2127166846952215
0.0004837810026659 0.0560988193283371 0.1438150361549498
0.9974364124437316 0.9659214674583254 0.2123813229407086
0.0016249832738715 0.9449596347296153 0.1432556980024921
0.0002953885373727 0.1024300667572960 -0.0001762484409747
0.0020812442547223 0.8972461814800033 -0.0001737252675066
0.0042548358191194 0.0008270231424503 0.8976887002354340
0.0043704225339616 0.0008276413536489 0.1019639761149995
0.1304396878645987 0.0322906063357790 0.9997961266288681
-0.0054026984048651 0.9321203260655126 0.2549488233892280
0.9925563608798165 0.0678962776367924 0.7440830849019967
-0.0057326684688362 0.9321167745913619 0.7447196335934715
-0.0105544047474031 0.2558180936826139 0.9318972591679757
-0.0104785502772914 0.2558169672031776 0.0677630027198291
0.9928929996092277 0.0679039341449228 0.2555831391824209
0.9910812772227290 0.7440079871938663 0.9319451610376263
0.9911597377830411 0.7440117948701218 0.0677253081716985
-0.0042160896305223 0.1611930034951230 0.8393490034810965
-0.0040179184294606 0.1611963238272629 0.1603020926642771
-0.0023866605391258 0.8400790895290038 0.8390914957030211
-0.0021901427924372 0.8400804928998324 0.1605660429027693
0.1835147878401491 0.0628030708370904 0.9997931311083762
0.1286912879272938 0.9634236325487793 0.9997853482174998

V-porphyrin*NO2
1.00000000000000
20.00 0.00 0.00
0.00 20.00 0.00
0.00 0.00 20.00
V C N H O
1 20 5 12 2
Selective dynamics
Direct
0.0273835746631555 0.0008514968084412 0.0000685776073855
0.9913344140599781 0.2123488141848939 0.0343666156141447
-0.0038361886889267 0.1434405054473585 0.0555151114336455
-0.0006158399948485 0.1225881894588814 0.1223059136881817
0.9934447681223403 0.7898732615893731 0.9657307225973149
-0.0019497327329933 0.8587072178932009 0.9444981799080717
0.0008833267317537 0.8796942695645444 0.8777476310534932
0.9934372699885211 0.7898726939843177 0.0343978005508345
-0.0019641210984117 0.8587075750114551 0.0556330654091245
0.9913549544173723 0.2123488324938277 0.9657470181512505
0.0008436297134950 0.8796949859162915 0.1223849320708418
0.9999565025748238 0.0354317930217213 0.7861637414526491
-0.0038033821470437 0.1434390189111619 0.9446043164808621
0.0037523706734362 0.0565681382685951 0.8553789675744147
0.0004197092199818 0.9668865001677996 0.7861575217270975
0.0044372686958589 0.9457876702031158 0.8553550903097605
-0.0005451073656350 0.1225860135617034 0.8778152072833666
0.9998358281418266 0.0354366364989067 0.2139586988666259
0.0036769647268857 0.0565708336020734 0.1447455884004896
0.0003111258023502 0.9668913801009128 0.2139695162111049
0.0043790294872263 0.9457901652966731 0.1447752462348727
-0.0023370394976009 0.1015577592707011 0.0000608207459241
-0.0004246408428582 0.9006494720139355 0.0000659106579946
0.0081543712176721 0.0012111766280763 0.8969487080038175
0.0081157126953353 0.0012120502314022 0.1031814501154659
0.1221783528798246 0.9753241060989215 0.0000601274607961
0.9968028660001922 0.9332218925494511 0.2565781322377857
0.9959645310793535 0.0690632714585097 0.7435663478014269
0.9969355235703855 0.9332151834093113 0.7435473592657219
-0.0113915572346014 0.2550289362917925 0.9320989395625792
-0.0114294164564693 0.2550293751868081 0.0680129954861478
0.9958093410900791 0.0690710079288445 0.2565516250506386
-0.0092058580720091 0.7471719656173030 0.9320923011397153
-0.0092188861542775 0.7471730793040358 0.0680367847850392
-0.0030350023112455 0.1616521851850036 0.8397391213635934
-0.0031287170052571 0.1616550973543593 0.1603805553056357
-0.0014953237618098 0.8406702687314190 0.8396169538958906
-0.0015454456514706 0.8406716783517014 0.1605155995911041
0.1259973474839353 0.0406745964100081 0.0000917582533821
0.1705519759796559 0.9377519059931778 0.0000350466518051

Co-porphyrin-with-NO3
1.00000000000000
20.00 0.00 0.00
0.00 20.00 0.00
0.00 0.00 20.00
Co C N H O
1 20 5 12 3
Selective dynamics
Direct
0.0117695251552704 0.9981494130280862 0.0013714354233582
0.0049707497416018 0.2092067872634487 0.0357143038748453
0.0061947483639051 0.1400228888026161 0.0565426676626902
0.0074491746316331 0.1194137232852271 0.1228648649135589
0.9957394963480563 0.7872524141206648 0.9667023902624222
0.0009613848943877 0.8563228797975077 0.9460499745741179
0.0075150383431822 0.8768547237396955 0.8799674325216069
0.9900651743037979 0.7874635040942722 0.0348368383308903
0.9924195550947037 0.8566370419222004 0.0557092918533864
0.0023028779940811 0.2091627694771904 0.9674171515594853
0.9912183640627164 0.8772546185401179 0.1220673591971782
0.0078768604481340 0.0324657546590270 0.7904103782636910
0.0027243442141469 0.1399971837053805 0.9465383745204067
0.0056795898444940 0.0532352587469564 0.8595798543604533
0.0104648258199119 0.9641574273699779 0.7903586930877751
0.0090911223308802 0.9432170682944822 0.8595114303431031
0.0031978582773613 0.1195117649547352 0.8801971037666959
0.0010081646877783 0.0321610130156789 0.2124919047859134
0.0034925718570705 0.0531109867205503 0.1433656736786323
0.9953930293830787 0.9640200031792426 0.2122029133232547
0.9950289805348603 0.9434447129799093 0.1429034297196610
0.0040281094475235 0.0975756704769776 0.0015381481428123
0.9975104657640671 0.8988145470457819 0.0008066626160607
0.0060846604747099 0.9982057794088769 0.9019184080095122
0.9990779839848501 0.9984870161821563 0.1008025666532383
0.1554349129686312 0.0184350075546955 0.9838135042413776
0.9917580679876959 0.9299392645321507 0.2544655076500906
0.0073697076955514 0.0665654465050073 0.7480216076298993
0.0122082269103032 0.9301510038361200 0.7479298202010978
0.0004777776331876 0.2515743473180191 0.9333886371242298
0.0054508122710191 0.2516421045631963 0.0697587176386854
0.0027883016888671 0.0660716163513941 0.2550106266627498
0.9961754974318265 0.7448086096289004 0.9326551184950669
0.9849607897591940 0.7451891490055305 0.0687189302278527
0.0025911487630578 0.1581127547590999 0.8416323441504271
0.0101926226562470 0.1578926402092132 0.1614606999489057
0.0100193104671255 0.8383026809620969 0.8414297074270592
0.9879793962825065 0.8387076461151012 0.1605705305131012
0.1047770624680855 0.9831755110557940 0.0137782809931891
0.2115207857688108 0.9980579596698671 0.0013713324329237
0.1433154232456576 0.0644503071231035 0.9444253832185621

Fe-porphyrin-with-NO3
1.00000000000000
20.00 0.00 0.00
0.00 20.00 0.00
0.00 0.00 20.00
Fe C N H O
1 20 5 12 3
Selective dynamics
Direct
0.0097206621444604 0.9977967771216008 0.9982813020967258
0.9981991886620493 0.2093356021420515 0.0325033195407051
0.9988558959444341 0.1403232941959987 0.0535337624870348
-0.0003509008983990 0.1197063844100655 0.1199853592804716
0.9868814860141265 0.7865681586203785 0.9648509837739795
0.9887399559516170 0.8555944954347265 0.9437851629851122
0.9863614346473514 0.8763154773603622 0.8773492255094593
0.9907263622491518 0.7865259688693267 0.0332718322741637
0.9947429568217211 0.8555322524653374 0.0540450970843509
0.9977603704327803 0.2093042478916635 0.9639758937847817
0.9986639359383134 0.8763415982213687 0.1203889447469435
0.9883429294663241 0.0320049144807264 0.7870872093360631
0.9977571622698120 0.1402409601508205 0.9430554401413364
0.9925476653369636 0.0530299358208217 0.8560213540186560
0.9852546853432691 0.9635559788010174 0.7873496756068387
0.9877872283581450 0.9427154208064615 0.8564280859557476
0.9958944049736058 0.1194734640550127 0.8766337310190965
0.0006181604786528 0.0324411824869741 0.2098660657615340
0.0000488803641442 0.0533108240622856 0.1407941518298622
0.0009574578252815 0.9639206649397657 0.2099980501937333
0.0001911353557497 0.9428245948741761 0.1409819075032326
-0.0014711574372270 0.0981864898749701 -0.0016434378058109
-0.0067131813253358 0.8975641934275023 -0.0011523348798775
0.9919380578937548 0.9979363655561523 0.8982143488449034
-0.0002591888802908 0.9980280142955559 0.0988048053869268
0.1609975470643157 0.0214191081761158 0.0130171420896223

```
 0.0013397927055554  0.9300921388922748  0.2526178328504280
 0.9877965773624450  0.0658217100330342  0.7444602803915816
 0.9816417471580875  0.9296084429140016  0.7449843826079835
 0.9972115400010274  0.2518244069507699  0.9300299716502536
 0.9982473695503375  0.2518856711021430  0.0664142274354392
 0.0008372987724769  0.0664071099526746  0.2523688502790087
 0.9831342274406895  0.7440841038107598  0.9310605564216369
 0.9907039998589572  0.7440023633713797  0.0672206055686431
 0.9960703187627982  0.1580335016195510  0.8380357824507682
 0.0002352533264571  0.1583553403089150  0.1584764850050709
 0.9831947485162483  0.8377161121096424  0.8389132761177758
 0.9995948530454623  0.8378233717070006  0.1590192258109173
 0.2170596678303745  0.0078488296971807  0.9899413383413523
 0.1090869526173859  0.9868721593222162  0.9859032638234029
 0.1491870180569144  0.0628993696672116  0.0579768426801678
```

Mn-porphyrin-with-NO3

```
   1.00000000000000
    20.00    0.00    0.00
     0.00   20.00    0.00
     0.00    0.00   20.00
   Mn   C    N    H    O
     1   20    5   12    3
Selective dynamics
Direct
  0.0160791679029574 -0.0018968500429196  0.0014428026700665
  0.0040151276693047  0.2104626884396769  0.0361929245337064
  0.0043238278298629  0.1413548621339722  0.0572525479268899
  0.0028195047698884  0.1200396089382526  0.1236749267732411
  0.9920577089347796  0.7865132466318195  0.9662069193736919
 -0.0028476628019783  0.8554857029224319  0.9453129380257469
  0.0003685002381086  0.8768072379199312  0.8789491878463146
  0.9902319974919345  0.7864307451094906  0.0347312434312248
 -0.0058387764449439  0.8553508785240315  0.0560732579189943
  0.0048416359993075  0.2105356307290260  0.9676532307041705
 -0.0061543789069588  0.8763929285642952  0.1226050769914575
  0.0059783259583307  0.0333004960641754  0.7893938530221661
  0.0059010860419613  0.1415138335921749  0.9464270383754674
  0.0063857616525839  0.0541540462893563  0.8585351691557163
  0.0043558535838480  0.9647724711349811  0.7890725062270127
  0.0035881149528900  0.9433032276279552  0.8580397075407474
  0.0068377169417594  0.1205154767560286  0.8799466862818126
  0.9969316785451402  0.0321700723141126  0.2135687843240714
  0.0003794238538739  0.0535246151483310  0.1446468914624767
  0.9943453871032355  0.9636665479932913  0.2132259193931934
 -0.0037792948129687  0.9427480191326452  0.1440921564473758
  0.0053784698601931  0.0997578493796969  0.0017734487523723
 -0.0020291202692037  0.8969931935816707  0.0008194367873719
  0.0050314666449817 -0.0014406791276902  0.8999476296060522
 -0.0001995800187701 -0.0017221519993124  0.1027557922836516
  0.1682716965512741  0.0154404537110360 -0.0153250082429037
  0.9913189154835677  0.9297196675761872  0.2556356524721521
  0.0067593402749853  0.0673084586104744  0.7469372939414950
  0.0034941486374520  0.9311635593282243  0.7462974640522144
  0.0047251832213143  0.2531756419635882  0.9338659821150549
  0.0030802136936369  0.2530365882032659  0.0700577676271644
  0.9963832765651953  0.0658299114017449  0.2563102208468813
  0.9901082104918404  0.7440436923194930  0.9322491200294087
  0.9866068393246737  0.7438596371340631  0.0684163950815666
  0.0076232333080333  0.1592150572513671  0.8414948516617374
  0.0025959842338968  0.1585494071571331  0.1623302374694159
 -0.0005627277574723  0.8383636618283570  0.8402387622317363
 -0.0090087854871718  0.8377366237238993  0.1610094603839874
  0.1181240155390957  0.9812519612922390  0.0140408751432263
  0.2261675451255933  0.9987760399290625  0.0001645074808428
  0.1535954680739375  0.0610169408124611  0.9442363418510235
```

Cr-porphyrin-with-NO3

```
   1.00000000000000
    20.00    0.00    0.00
     0.00   20.00    0.00
     0.00    0.00   20.00
   Cr   C    N    H    O
     1   20    5   12    3
Selective dynamics
Direct
  0.0152965583827193  0.9971254893611164 -0.0000959533444557
  0.0008819459240859  0.2102427492948182  0.0341966699925647
  0.0009481661864730  0.1410468503602597  0.0553153072436015
  0.0018220440795298  0.1193895032817829  0.1218097498383716
  0.9877777221873183  0.7855007094468772  0.9656153137470417
 -0.0069790041689066  0.8544416779032561  0.9444735069882594
 -0.0048208932403523  0.8757085918809865  0.8779624012450197
  0.9877512087589032  0.7854999014718637  0.0341639269941525
 -0.0070100576829702  0.8544411160299247  0.0553136163718592
  0.0008837644232267  0.2102465125915428  0.9656191370520768
 -0.0048478625661340  0.8757039147201156  0.1218274283948988
 -0.0006337314783903  0.0318811959915957  0.7871438315989512
  0.0009433381083510  0.1410497048514250  0.9444918843564678
  0.0013103023345655  0.0529544137077982  0.8563035184715577
  0.9976224192015809  0.9633191466025603  0.7870639593813915
 -0.0015761595758309  0.9420642422769916  0.8561928175062327
  0.0017871178918606  0.1193971711923528  0.8779927339466206
  0.9994939137570121  0.0318618606622457  0.2126585502866544
  0.0013591521169573  0.0529442839031309  0.1434966154636432
  0.9977238373339222  0.9633005438985549  0.2127323727286702
 -0.0015637283734948  0.9420538036884258  0.1436028641024587
  0.0001648279763208  0.0993855909349156 -0.0000979742840039
  0.9955558439646058  0.8962070833562344 -0.0001062683135785
  0.0009247838255913 -0.0026052210382725  0.8979723844179701
  0.0009142560744905 -0.0026132195061938  0.1018255724329330
  0.1610615329776404  0.9677519506719791  0.0001200677244653
  0.9955959206298312  0.9296632065137972  0.2554650079576118
 -0.0011273800218327  0.0656401129397263  0.7444696428813639
  0.9954347325346007  0.9296768365993974  0.7443350537557082
  0.0009539960968462  0.2529740470893101  0.9319168149624484
  0.0009465742823930  0.2529678391895250  0.0678982174015039
 -0.0009143077036721  0.0656171867921331  0.2553390056206085
 -0.0156390068393031  0.7429260536617730  0.9318957497533004
 -0.0156944962897039  0.7429224699399460  0.0678810417319252
  0.0022353275339633  0.1580120308740731  0.8393965202845858
  0.0022965420564369  0.1579986170851161  0.1604114451526161
 -0.0065880184327198  0.8370075215680562  0.8394967825575439
 -0.0066177119397101  0.8369995919045593  0.1602888732923909
  0.1104613644656506  0.0148071382235103 -0.0000903488326049
  0.1462532503864215  0.9075566190239703  0.0003507054932146
  0.2179464148216858  0.9921021610588212  0.0000514536439653
```

V-porphyrin-with-NO3

```
   1.00000000000000
    20.00    0.00    0.00
     0.00   20.00    0.00
     0.00    0.00   20.00
    V    C    N    H    O
     1   20    5   12    3
Selective dynamics
Direct
  0.0307017377755257  0.9996132494735835  0.0002139259347068
  0.9885036716120874  0.2094934393959065  0.0344846555484368
 -0.0050658055046714  0.1407972620319288  0.0556356730160458
 -0.0000403613604594  0.1205930888344336  0.1224732848951714
  0.9882251998539122  0.7898356811084549  0.9659006974935107
 -0.0052343935826103  0.8585221076206271  0.9447597899075411
 -0.0000739631563014  0.8787170831366220  0.8779343142277231
  0.9879983268911807  0.7897961743855489  0.0345256962205970
 -0.0056060246927508  0.8584701198569474  0.0557602650549203
  0.9884316933855372  0.2095232177427501  0.9658603708599620
 -0.0007762519535048  0.8786347112810604  0.1226213015763434
  0.0026683722106964  0.0339030532424013  0.7858443681913521
 -0.0051953169021731  0.1408439929608968  0.9446384115600490
  0.0060717750079715  0.0549517350418891  0.8551173111059737
  0.0027439389040406  0.9653718898393133  0.7858917182384313
  0.0061953447082535  0.9444214498198421  0.8551914499710760
 -0.0001813774627456  0.1206767833230975  0.8777955897281993
  0.0022308236009081  0.0339362339642582  0.2145005513869446
  0.0060066245065247  0.0548729275205729  0.1452128000091662
  0.0019576783050371  0.9654066993672497  0.2145574794388705
  0.0055672659872809  0.9443447857912394  0.1453012124265396
 -0.0031828537175723  0.0987080859567170  0.0001119892428510
  0.9966210452561334  0.9006050922271812  0.0002903583248142
  0.0106568891275424 -0.0002773713693700  0.8967563951872730
  0.0104627237578895 -0.0004454466898164  0.1036625629832827
  0.1589886017592044 -0.0004448248660701  0.0000374075951570
  0.9978264905867610  0.9317552509012282  0.2571316762299580
  0.9988656885126400  0.0675589505606492  0.7432408990500650
  0.9989761758264336  0.9316431691844629  0.7433480055549855
 -0.0151016178559251  0.2521435358079788  0.9322034185710900
 -0.0149491868527717  0.2520778289138804  0.0681868851635737
  0.9983939649334022  0.0676738792723343  0.2570405244892370
 -0.0151818900640262  0.7472504248480828  0.9321835703243656
 -0.0156418008371990  0.7471925760969792  0.0681808124936738
 -0.0031144493953286  0.1599742105898414  0.8399806769212199
 -0.0029545045717925  0.1598961242334783  0.1602765381654293
 -0.0029795594591302  0.8394095381492053  0.8401320708015957
 -0.0039204990881424  0.8393516029811077  0.1604249603841289
  0.1216807937448642  0.9457984067566559 -0.0021169939158678
  0.1217242809413086  0.0533351054088066  0.0023238869853199
  0.2201852492619459 -0.0004608247019561 -0.0000665113437197
```

Ti-porphyrin-with-NO3

```
   1.00000000000000
    20.00    0.00    0.00
     0.00   20.00    0.00
     0.00    0.00   20.00
   Ti   C    N    H    O
     1   20    5   12    3
Selective dynamics
Direct
  0.0346834182467221  0.9977043376700092  0.0002180599545620
  0.9924491026555583  0.2112252806034360  0.0344928646091567
 -0.0031057205070744  0.1422980190643319  0.0555946393457117
 -0.0013722104577059  0.1216158120075172  0.1225899568861721
  0.9907871197079021  0.7873682809089397  0.9657493064199928
 -0.0030436641661195  0.8560361614908332  0.9445932258821040
 -0.0004797330619374  0.8782109004141189  0.8780913882585004
  0.9907194318768919  0.7873645548234207  0.0345830394373336
 -0.0031581999294983  0.8560315883606309  0.0557577474861183
  0.9924899908380640  0.2112248089496301  0.9658615884504943
 -0.0007345766441453  0.8782014877792319  0.1222681435678917
  0.9972308092685305  0.0339403204945535  0.7865989499879583
 -0.0030300192319704  0.1423011583462473  0.9447626670596924
  0.0023549128698384  0.0556653364246352  0.8554095082966896
  0.9982624717277903  0.9653747305235314  0.7870656166528975
  0.0034560335079172  0.9444759487394457  0.8562115376290219
 -0.0011887866828417  0.1216203835344600  0.8777720817016186
  0.9968679656704638  0.0339353227606143  0.2137614495994156
  0.0021094851177802  0.0556593800837823  0.1449594364709964
  0.9978675816164183  0.9653687960582259  0.2132955178480477
  0.0031676479932870  0.9444657984070058  0.1441576698346498
 -0.0013517187489638  0.1003200940649809  0.0001802395227276
  0.9997072665844434  0.8976450791488477  0.0001810355398485
  0.0080779860191943  0.0005729193272568  0.8973923037028121
  0.0078805220735748  0.0005626425117842  0.1029869157173043
  0.1642316710195323  0.0008152702453116  0.0002317924407123
  0.9938627842521430  0.9313988436103097  0.2556265665572938
  0.9921158459730801  0.0672077808171072  0.7438302221707610
  0.9943348352368910  0.9314018025361528  0.7447315916006830
 -0.0105118695853706  0.2538449108645616  0.9321520332837969
 -0.0105865695955462  0.2538435884770034  0.0681998568905220
  0.9917070518990255  0.0672027679616147  0.2565249207708440
 -0.0126725031837799  0.7446600602940888  0.9321854553368893
 -0.0127972746989551  0.7446506876969147  0.0681319658121449
 -0.0042060638019281  0.1607430098088473  0.8398119442071619
 -0.0044199138867427  0.1607439101234628  0.1605400845302617
 -0.0040792476914298  0.8398950213890857  0.8393475009432160
 -0.0043997144858495  0.8398797258640209  0.1609979095076395
  0.1263435372734934  0.9460465585259598  0.0001659572329193
  0.1275659516423337  0.0544076660665708  0.0003493414841188
  0.2253488632889611  0.9995402532215236  0.0001879673693125
```

Co-porphyrin*N2H

```
   1.00000000000000
    20.00    0.00    0.00
     0.00   20.00    0.00
     0.00    0.00   20.00
   Co   C    N    H
     1   20    6   13
Selective dynamics
Direct
  0.0127491374132892  0.0021224263304459  0.9978921179338071
  0.9949787359383534  0.2134736866236498  0.0316247330209349
 -0.0024547485708061  0.1442847239345275  0.0526686598375202
 -0.0030512440860662  0.1236758274787321  0.1190872566679246
  0.0036426375926778  0.7910913279979037  0.9639734851817058
  0.0014474206930939  0.8602570074444693  0.9429260837069203
 -0.0034456184300024  0.8807176882357314  0.8766435116221152
  0.0119122738635073  0.7912459794082285  0.0318654339877552
  0.0135434542854071  0.8605886437413687  0.0524270827895023
  0.9987789376363652  0.2134029389778957  0.9633635199225039
  0.0191270560752393  0.8816970822530334  0.1186046677628501
  0.9989250748340441  0.0362167008707469  0.7865362227002943
  0.0027705947099436  0.1441161773360988  0.9427713152901878
  0.0028051280164640  0.0569676558651700  0.8557868043484764
  0.9950101419672797  0.9679613562419078  0.7866172551879291
 -0.0026150289564251  0.9470826422010841  0.8558687982209889
  0.0048025200049529  0.1233739428118748  0.8763994586121167
  0.0044477569830778  0.0365751722642513  0.2088870155566598
  0.0019806422368755  0.0574539360281246  0.1396913328063773
  0.0126894131627366  0.9686737774900854  0.2088706721481893
  0.0139644884261937  0.9479553446489641  0.1395743812194915
  0.0018506128891577  0.1020215025685783  0.9978286239240629
  0.0068405663687611  0.9026956814343142  0.9976620545231990
  0.0017211272260428  0.0020069392591135  0.8980045646530858
  0.0071851716897376  0.0026170964437802  0.0973602283325544
  0.1064610616858229  0.0054913991189572  0.9939747694889000
  0.1445240936190803 -0.0281447029328027  0.0270291954986814
  0.0165161008209014  0.9347976807561176  0.2512736621557523
  0.9985300910573098  0.0702916479627638  0.7440943914910432
  0.9909824787771996  0.9339986253683542  0.7442815842361272
  0.9982090320662378  0.2557532740819504  0.9291956914260944
  0.9908948634764878  0.2558821664539151  0.0654922302279460
  0.0005311773896415  0.0703283959164289  0.2513980005386030
 -0.0004147864172077  0.7485337415174201  0.9303163094659305
  0.0155637787505091  0.7489118925849351  0.0658634638392331
  0.0056338166825337  0.1619262732929023  0.8377854200342726
 -0.0066457946389737  0.1621164259031654  0.1576563560675820
 -0.0071252175050318  0.8420599674355714  0.8383052366348409
  0.0246882638255519  0.8433400436574281  0.1570307447407619
  0.1249792884400109  0.9372089109928080  0.0614176641970863
```

Fe-porphyrin*N2H

```
   1.00000000000000
    20.00    0.00    0.00
     0.00   20.00    0.00
     0.00    0.00   20.00
   Fe   C    N    H
     1   20    6   13
Selective dynamics
Direct
  0.0173055016714633 -0.0000057727785484  0.0019395677743692
  0.0006600857861731  0.2118897581593414  0.0364833682175570
  0.0026076733489568  0.1425126974535721  0.0573251332996637
 -0.0000439273633215  0.1215489791502706  0.1236898390909434
  0.0024242300595487  0.7878301979018149  0.9681282374674135
  0.0054806600714398  0.8571166751839008  0.9471271592933208
  0.0061551313966085  0.8781206485463211  0.8807441987071152
  0.0006197162783028  0.7880822984990273  0.0364812024617492
  0.0025902977983548  0.8574677385701052  0.0573144939759837
  0.0025256849979677  0.2121354889949653  0.9681333677461044
 -0.0000219704342247  0.8784348788164664  0.1236821599357183
  0.0040036673429890  0.0342731926607240  0.7908711276094840
  0.0055904750831697  0.1428529125307373  0.9471387171936276
  0.0057387751509469  0.0552866232311093  0.8599152848560735
  0.0039501246424842  0.9656989431374384  0.7908685446471004
  0.0056556429455050  0.9446764763139519  0.8599062752596458
  0.0062964078852492  0.1218436040161072  0.8807588259070979
  0.9945165188827586  0.0342316758808937  0.2138727628554532
  0.9985274381726578  0.0550654615957090  0.1447067876886300
  0.9945282367619133  0.9657475055019877  0.2138682963387226
 -0.0014594130085947  0.9449189473950070  0.1447039292937922
  0.0057584123501459  0.1005996931570313  0.0021409081123414
  0.0056985584508042  0.8993685532583957  0.0021251692359120
  0.0061713788417031 -0.0000197152847724  0.9021448037495362
  0.0005190649644995 -0.0000058825940997  0.1027435230445060
  0.1050115407221366  0.0000445165986146 -0.0015101538423456
  0.1536605711523192  0.0005148776946299 -0.0381937276345159
  0.9919554324853719  0.9318299808888086  0.2563577625014367
  0.0026179291578335  0.0681459826182974  0.7482904582383452
  0.0025222691670256  0.9318214773866801  0.7482858641056889
  0.0011730889057763  0.2547346148706883  0.9342809119924561
  0.9974829001818406  0.2542573503505566  0.0704945375255850
  0.9919306399242223  0.0681336846895978  0.2563672975769364
  0.0010474196407318  0.7452320700972268  0.9342740791956510
  0.9974751577302787  0.7457220451298440  0.0705069155544435
  0.0059429388808785  0.1603161072171239  0.8420376073165147
 -0.0021497855797204  0.1601769737340024  0.1622160398630156
  0.0057503939823638  0.8396469221342251  0.8420268194630505
 -0.0021197445827272  0.8398049854880619  0.1622042994248634
  0.1445353761541466  0.0011678318041992  0.9102476049570160
```

Mn-porphyrin*N2H

```
   1.00000000000000
    20.00    0.00    0.00
     0.00   20.00    0.00
     0.00    0.00   20.00
   Mn   C    N    H
     1   20    6   13
Selective dynamics
Direct
  0.0181172648309490  0.0000211800528934 -0.0001309313643799
  0.9995228388694853  0.2125336128754210  0.0340908945093983
  0.0021366020963525  0.1431979530912496  0.0550250995888856
  0.0015281997223129  0.1218002075233019  0.1213323867521151
  0.9996074472477420  0.7874931683072630  0.9656776678508447
  0.0021959360258997  0.8568329961658540  0.9447563353002777
  0.0013876593802877  0.8781928552308848  0.8784300286555879
  0.9996377151377599  0.7874985958403380  0.0340874379737729
  0.0022636348782241  0.8568362246574383  0.0550172531767534
  0.9994990383889470  0.2125320255218964  0.9656805662347454
  0.0016306909100286  0.8782349635706402  0.1213262735068463
  0.9991076072805070  0.0342878864327187  0.7882948585374123
  0.0020832061116263  0.1431905545739898  0.9447666540090389
  0.0005186736025858  0.0552714917655412  0.8574334334135802
  0.9991297291426545  0.9657371922631364  0.7882878129727328
  0.0005565173540718  0.9447441326550244  0.8574253493008994
  0.0013051548898847  0.1218237474022828  0.8784433446242722
  0.9994025939784893  0.0342676588521565  0.2113863404523752
  0.0008537144908189  0.0552455157219205  0.1422345719354730
  0.9994297432970167  0.9657624611879037  0.2113860207289633
  0.0009042832533113  0.9447878399063810  0.1422314241842196
  0.0037242988395827  0.1010626036653089 -0.0000863108582092
  0.0038435623907030  0.8989682191581581 -0.0000998866577192
  0.0006951172150344  0.0000056683580701  0.8996062620744425
  0.0010766921965362  0.0000188356770699  0.1000249981329985
  0.1001171856096083  0.0000856436057729  0.9987567305583808
  0.1613741040563318  0.0000716835329257  0.0032940427511058
  0.9984896052959168  0.9319419294848793  0.2540044842671972
  0.9981117027557929  0.0681284191509440  0.7456811202273640
  0.9981506650196906  0.9319068132006324  0.7456647279281140
  0.9971846348151917  0.2550729709640316  0.9318114353063726
```

```
0.9972247815389200 0.2550760580609591 0.0679500583858752
0.9984396169669818 0.0680903851381645 0.2540016008877552
0.9972540249602480 0.7449502781506112 0.9318105206004250
0.9973073628243416 0.7449601208190825 0.0679523200281984
0.0004878321309127 0.1602629559363425 0.8396620858797081
0.0008451693857554 0.1602075561524003 0.1601427162442984
0.0005721869741548 0.8397494800287564 0.8396518967205741
0.0009458987389718 0.8398236444517811 0.1601332037741089
0.1859718073963475 0.0005454708658894 0.9575551714051971
```

Cr-porphyrin*N2H

```
1.00000000000000
20.00 0.00 0.00
0.00 20.00 0.00
0.00 0.00 20.00
Cr C N H
1 20 6 13
Selective dynamics
Direct
0.0190646150837175 0.0000284648800930 -0.0001512846365104
0.9993078554476964 0.2130489976221913 0.0340723772458880
0.0023904192019991 0.1437358431814438 0.0552288469584035
0.0016033002274230 0.1220348920124338 0.1215329665165228
0.9992593489954442 0.7869664315575414 0.9656411259374620
0.0021885084008939 0.8562782646275036 0.9444468888327030
0.0011197503090294 0.8779583352659807 0.8781352946576481
0.9994350330059392 0.7869851980491073 0.0340681683696032
0.0024739187019077 0.8563025251423205 0.0552224717277436
0.9991350420755652 0.2130661181876657 0.9656438234220216
0.0016777320392779 0.8780005014564792 0.1215265090043914
0.9982597241327862 0.0343358729729036 0.7875583288957559
0.0021102035932540 0.1437590681703156 0.9444478451862132
0.0002554671037357 0.0554874607011821 0.8566210520597833
0.9982799218880902 0.9656953192978407 0.7875590641767211
0.0002881866987210 0.9445475231623762 0.8566233156201593
0.0010532214456367 0.1220753402612425 0.8781362539565747
0.9989216677225319 0.0343183698689621 0.2120502430507464
0.0009398986593055 0.0554559878803353 0.1429727856425812
0.9989421117738001 0.9657104294766108 0.2120465138677178
0.0009751879031895 0.9445797632307997 0.1429674548296727
0.0043987624661505 0.1019535912057599 -0.0001661393133582
0.0044549584205041 0.8980882008770991 -0.0001704579021479
0.0008541083366872 0.0000190152280902 0.8984241930275471
0.0015434753903057 0.0000203669695284 0.1011378889477844
0.1032050082696159 0.0000886889011514 0.9988360413932674
0.1647789231915582 0.0001119887356867 0.0039581122293674
0.9975703035127974 0.9319735647579630 0.2547322420360250
0.9968524290970611 0.0680330434749029 0.7448418933643807
0.9968794499728520 0.9319927397069299 0.7448447528058749
0.9964648939813643 0.2556927474243898 0.9319162869134312
0.9968392370900803 0.2556283986930739 0.0678762071009787
0.9975406794964572 0.0680472522147371 0.2547394625831290
0.9966053130113252 0.7443394409823421 0.9319123878142083
0.9969891609124567 0.7444059452651651 0.0678746322224492
-0.0002315860666950 0.1605761455207632 0.8394330392832335
0.0004262848336103 0.1605069035193667 0.1602777421016465
-0.0001400737438909 0.8394611246079812 0.8394295579422473
0.0005266117255739 0.8395309665072395 0.1602729185284138
0.1893954456922192 0.0003801684025160 0.9581791935997241
```

V-porphyrin*N2H

```
1.00000000000000
20.00 0.00 0.00
0.00 20.00 0.00
0.00 0.00 20.00
V C N H
1 20 6 13
Selective dynamics
Direct
0.0252327773041334 0.0000253488156699 -0.0001348075700561
0.9980574863783318 0.2131795116486004 0.0342890956958207
0.0017880575476250 0.1440457694625084 0.0554774271476771
0.0008473138000983 0.1220273455840155 0.1219975085935271
0.9980671409149406 0.7868501990243255 0.9656382747575715
0.0016530963876177 0.8559838047277968 0.9444202479138462
0.0004921058266958 0.8779762251898392 0.8778948344115117
0.9981701208919237 0.7868563730383945 0.0342882336537138
0.0018527658937921 0.8559929474986371 0.0554728431686251
0.9979619248374592 0.2131843952961897 0.9656372805337434
0.0009163202428165 0.8780114390171049 0.1219928706232446
0.9967194011660993 0.0343223593233047 0.7867211245614081
0.0016002826391488 0.1440535046594600 0.9444197202114053
0.0002078827416012 0.0555107617239909 0.8559301208858948
0.9967324031182104 0.9657108202090079 0.7867227740020224
0.0002297411463798 0.9445269215144289 0.8559339187393781
0.0004411575381231 0.1220592918753278 0.8778947158615267
0.9972860821454885 0.0343061500020758 0.2131358470896202
0.0007522564480657 0.0554796420150055 0.1438990639872822
0.9973064345002918 0.9657244914202511 0.2131329162089587
0.0007860600098238 0.9445600685841672 0.1438935299403242
0.0044871590242999 0.1025186492421149 -0.0000509313050376
0.0045136111573686 0.8975271402025692 -0.0000550596415931
0.0023729659728381 0.0000216965090099 0.8974410455280302
0.0029210260518321 0.0000248048151625 0.1023570190658834
0.1141076221057077 0.0000554353931091 0.9985532355654095
0.1759410752468543 0.0000143312459998 0.0029030904771860
0.9948460691560472 0.9321022553062214 0.2558684994796078
0.9942116362839880 0.0679192227674790 0.7439715160785815
0.9942269821639486 0.9321028959569346 0.7439796611222576
0.9950081191224183 0.2559475667719514 0.9320967137797674
0.9951914549060500 0.2559085830960815 0.0678949147702565
0.9948169147352712 0.0679147495393648 0.2558797611795656
0.9951444390203641 0.7440841723257546 0.9321002352840086
0.9953429053041516 0.7441257978731873 0.0678940480092583
-0.0018025292945123 0.1606672374863900 0.8393137136809354
-0.0013090839510093 0.1606167680586478 0.1606054400868810
-0.0017197392772030 0.8393738683266992 0.8393108979930713
-0.0012018689215869 0.8394270562043110 0.1606034494583647
0.1984349297144821 0.0004813982489266 0.9553752089705211
```

Ti-porphyrin*N2H

```
1.00000000000000
20.00 0.00 0.00
0.00 20.00 0.00
0.00 0.00 20.00
Ti C N H
1 20 6 13
Selective dynamics
Direct
0.0275898246495018 0.0000174078387026 -0.0000784649493311
```

```
0.9971324738815698 0.2136437489250467 0.0343096833213776
0.0016677777506762 0.1445916578509910 0.0557407149754115
0.0007363688803842 0.1222703547277679 0.1222684648484154
0.9970621529145808 0.7863390031584778 0.9656366305883878
0.0014668297007323 0.8553681300718665 0.9441063825932148
0.0001897112168224 0.8777099374948018 0.8775957054049700
0.9972436423704359 0.7863932721104849 0.0343115712921213
0.0017495690122409 0.8554446252314525 0.0557332686497754
0.9969620099054254 0.2136893622133640 0.9656371532259895
0.0008059533003263 0.8777754887108830 0.1222587664001395
0.9960948148455326 0.0343675429610155 0.7860375786305899
0.0014026510825085 0.1446564948250940 0.9441143234954035
0.0002038763660413 0.0557411280902073 0.8551460257892201
0.9961081829807641 0.9656719247100036 0.7860299435313612
0.0002244358633931 0.9442850077799662 0.8551364504027978
0.0001458114828154 0.1223141426490050 0.8776045342968470
0.9966732936744990 0.0343539863721925 0.2138322491972753
0.0009321689364217 0.0557263331505524 0.1447220482553264
0.9966972591299288 0.9656882315444650 0.2138323460178777
0.0009697472835522 0.9443181934209990 0.1447214226226708
0.0049669944873954 0.1034220575742064 -0.0001044074247527
0.0050191853602591 0.8966145246134135 -0.0001190573004091
0.0029062737492598 0.0000073882552713 0.8963033247320819
0.0037443022045348 0.0000237173257545 0.1035553799696577
0.1194545092731969 0.0000257186670793 0.9983895206480283
0.1816274786541903 -0.0000322925069611 0.0029847846008850
0.9936248641181028 0.9322295714610530 0.2566486481259939
0.9930438377580959 0.0678450561330223 0.7432270841867517
0.9930837431663064 0.9322014790175975 0.7432133314675756
0.9933265728954623 0.2565199772473501 0.9322531118473661
0.9937243731572404 0.2564379376256706 0.0677643716375049
0.9935618685308009 0.0678100465981713 0.2566489274198790
0.9934474324356539 0.7435032814978209 0.9322561027347195
0.9938667505450796 0.7436016907057887 0.0677736328508445
-0.0026296059094557 0.1609466711912416 0.8390882180960305
-0.0019151740254965 0.1609018486084174 0.1607933154110403
-0.0025758250741646 0.8390725818803599 0.8390814009326574
-0.0018292739632687 0.8391390598047582 0.1607822450628121
0.2041276374086311 0.0005847104626601 0.9554632664114924
```

Co-porphyrin*NH2

```
1.00000000000000
20.00 0.00 0.00
0.00 20.00 0.00
0.00 0.00 20.00
Co C N H
1 20 5 14
Selective dynamics
Direct
0.0080070891836179 -0.0000522317103796 0.0011622709349478
0.0000645392096772 0.2109073384450155 0.0357866654714439
0.0007559840161629 0.1416835526343835 0.0565885404827927
0.0001489081141215 0.1211926094964786 0.1230036486103996
0.0004198810965231 0.7886462353507706 0.9673796538664249
0.0013808566062968 0.8578372835348846 0.9464091671035951
0.0021449730596915 0.8783965039318964 0.8800366456337340
0.0000104301877190 0.7888546284506307 0.0357504634711370
0.0007678161255064 0.8581411941660045 0.0564660504860953
0.0007160771090607 0.2110566701206295 0.9674027429640368
0.0004149644351283 0.8786636609572197 0.1228679768600224
0.0022601531992251 0.0340869166831890 0.7903528111501459
0.0017572602108517 0.1418918471307991 0.9464465572930411
0.0024490509108620 0.0549665446954724 0.8595322305637881
0.0021343868230211 0.9657042800926592 0.7903487546919640
0.0022380862762264 0.9448107080466071 0.8595323268105149
0.0025346949506300 0.1213470957650910 0.8800702053491777
0.9982390629483788 0.0340386723336558 0.2129191645682757
0.9992390547287147 0.0548521808552508 0.1436006472468162
0.9983668202201429 0.9656981967184253 0.2128533004389560
-0.0005685330857071 0.9449952054147784 0.1434984103154319
0.0013209165775192 0.0993249175327086 0.0014623748300568
0.0010945382212060 0.9004110749129141 0.0013509323351431
0.0018319447253127 -0.0001065523926627 0.9020160772211400
0.9991601324874188 -0.0000455053264427 0.1012224623225771
0.1038823509283869 0.9993554425057546 0.0023405248504430
0.9977135285198304 0.9315594757862689 0.2552419713568656
0.0019425007257530 0.0681428694369703 0.7479152333907992
0.0016905437248020 0.9316461355888431 0.7479077824704283
0.0002499660509994 0.2535576247781863 0.9334160049236163
0.9988683177540660 0.2532955976094476 0.0699012393292199
0.9974814142178658 0.0681069127903660 0.2553635833559807
0.9998870464649345 0.7461324854596804 0.9334093812799095
0.9989562306491093 0.7465123003667847 0.0699303103332864
0.0027033774166350 0.1599317926436882 0.8414838016463767
-0.0000663840821779 0.1598629388729249 0.1615105202462404
0.0022236396333804 0.8398407914653051 0.8414092829802936
0.0003568549319148 0.8399992526996858 0.1613952835497674
0.1173260675962540 0.0447172781979837 -0.0186831156432744
0.1170799571309183 0.9658570739581369 0.9651481149083876
```

Fe-porphyrin*NH2

```
1.00000000000000
20.00 0.00 0.00
0.00 20.00 0.00
0.00 0.00 20.00
Fe C N H
1 20 5 14
Selective dynamics
Direct
0.0118030821956143 0.0001118288919235 0.0010869675538923
0.0018968604961144 0.2119831568257693 0.0356711027320820
0.0026082633914297 0.1425555096058769 0.0565037231524754
-0.0002446454564714 0.1216332023290803 0.1227349666116292
0.0025924580608764 0.7880696755502037 0.9671559509711367
0.0034990278516190 0.8574555169435493 0.9461453061967440
0.0015230242364945 0.8783894766886750 0.8798785057296350
0.0026814279287573 0.7881952181869357 0.0354759812115832
0.0035023953348765 0.8576088423860238 0.0563424010954593
0.0025213596388089 0.2121470331014473 0.9673507817493908
0.0009633710932340 0.8784704738978721 0.1226007586517839
-0.0015167563197309 0.0344853617340887 0.7901940884895955
0.0036270366511077 0.1427867230015682 0.9463042447345028
-0.0002663785459069 0.0553826367718089 0.8592049453113871
-0.0017648806676255 0.9658942664779721 0.7901593634561626
-0.0006071704536873 0.9449299404596092 0.8591435587416179
0.0020779550447295 0.1218953006191513 0.8800115898248703
0.9950426527468502 0.0342615849964587 0.2125876682719222
0.9970817730289980 0.0551395555040347 0.1435232129281591
0.9954955304552119 0.9657027250143093 0.2125538132376352
-0.0022564651984832 0.9449193986847308 0.1434637495842243
```

```
0.0041748320175506 0.1002540352161532 0.0013419617134687
0.0045373758601386 0.8999488421865559 0.0012026592297067
-0.0009184308615636 0.0001347309465376 0.9015217587929795
0.9973437493656142 0.0000518590864236 0.1012385253206784
0.1031551503300948 0.0004590886973529 -0.0002721899686765
-0.0052392747339878 0.9317564542018769 0.2550969428076610
0.9980788648601481 0.0684198139919403 0.7476449196160038
0.9975937260480189 0.9319892430377767 0.7475898107863674
0.0014120350497461 0.2547240811920188 0.9334635990037616
0.0002363257553200 0.2544075812479438 0.0697282108583743
0.9938640128986500 0.0681192798040343 0.2551807380943277
0.0011344092914059 0.7455141643019717 0.9332495575727968
0.0013632636469358 0.7457539357894566 0.0695199458090765
0.0020307665682015 0.1604048906262740 0.8412984954104682
-0.0009420792759186 0.1601497080565688 0.1614288972857085
0.0011199951065033 0.8399080489355431 0.8411432266479951
0.0007378439824354 0.8399218106315972 0.1612656250172399
0.1234133644740047 0.0427172002278962 -0.0212517672538316
0.1238286481038630 0.9591688041509937 0.9772664030200040
```

Mn-porphyrin*NH2

```
1.00000000000000
20.00 0.00 0.00
0.00 20.00 0.00
0.00 0.00 20.00
Mn C N H
1 20 5 14
Selective dynamics
Direct
0.0129148924270581 0.0001394666335729 0.0022933507626733
0.0007912418752622 0.2120843530803002 0.0357928372348711
0.0022158084081102 0.1427472421818563 0.0567862766829842
0.0002688332960967 0.1217587187562484 0.1231179161287881
0.0001325564504219 0.7877745096622297 0.9671985441665061
0.0017719210790872 0.8569799662783456 0.9458399043685077
0.0013052207597873 0.8780960486908401 0.8796020134311940
0.0011765035139843 0.7880067945601600 0.0355790431801118
0.0026454230801590 0.8573232948367469 0.0566177601265811
0.9998601049301844 0.2123640515642291 0.9674047402244458
0.0006179103267021 0.8782242330482046 0.1229673808944842
0.0007558700370412 0.0344907201428404 0.7896122464725001
0.0015163236947012 0.1431881890888902 0.9459812609261881
0.0007223539531696 0.0554432863541572 0.8585704326088140
0.0007100240314290 0.9657950067580064 0.7895751779237991
0.0006995967958026 0.9447782757438815 0.8585141313264895
0.0012535364361464 0.1221039040177364 0.8797304247092899
0.9956299298065610 0.0342919773341542 0.2136191261982820
0.9982089989914683 0.0552989021445187 0.1447058500867248
0.9957023556988859 0.9655682171083068 0.2135705295494875
-0.0016413868592392 0.9446481954896842 0.1446374537232108
0.0030647158344791 0.1007409479268398 0.0012040060613291
0.0034601126992718 0.8993960290664407 0.0010754651885106
-0.0000634917053037 0.0000854004537959 0.9006545888221084
0.9989031875825779 -0.0000002018280370 0.1027344289566027
0.1070659693913621 0.0007757631371860 -0.0001747583108330
-0.0053652058863346 0.9317635915681546 0.2562037427884992
0.0004179105625647 0.0683221305668665 0.7469796932358658
0.0003122513722612 0.9320076639211035 0.7469136605347714
0.9977495298501324 0.2550950135908412 0.9337524096028428
0.9997722427243725 0.2545521929070121 0.0698190606967157
-0.0055039627247488 0.0680328805457390 0.2562989754777604
0.9978921330714279 0.7450783428727255 0.9335128901262774
0.0001406755650553 0.7455194926911730 0.0695780953215375
0.0011611500478753 0.1605981986549515 0.8410195063866188
-0.0007565381815742 0.1605333038787347 0.1615322430956820
0.0011347719468689 0.8396304484468180 0.8408618524547387
-0.0003603639814330 0.8393990428386775 0.1613346428332010
0.1251744766559288 0.0428728142918450 -0.0234579151324543
0.1257269164423771 0.9603125909942267 0.9741910111342940
```

Cr-porphyrin*NH2

```
1.00000000000000
20.00 0.00 0.00
0.00 20.00 0.00
0.00 0.00 20.00
Cr C N H
1 20 5 14
Selective dynamics
Direct
0.0152850788870417 -0.0000435657453841 0.0013076857536982
0.9988678207580608 0.2127825808483713 0.0357394675580873
0.0009277904271780 0.1436345639424746 0.0569148121464142
-0.0001955158539790 0.1218259122810305 0.1234697191191756
0.9990655711263242 0.7869617583627555 0.9670529002981030
0.0013835155689098 0.8560856736566443 0.9458083578294517
0.0014111957543361 0.8779113656275315 0.8792909464147388
0.9989850123209102 0.7870796788948652 0.0356884617185478
0.0011459861630993 0.8562610577793157 0.0567998314670708
0.9992563780944907 0.2128595018465698 0.9670947540736579
0.0002105977239770 0.8780685739099062 0.1233451929578649
-0.0000267576339193 0.0342367894613126 0.7883904007167802
0.0016409250116149 0.1437377374560484 0.9458915975860880
0.0016403744154633 0.0554048992176391 0.8576506439322742
-0.0001776794062033 0.9656752594779234 0.7883730445221208
0.0014089340830514 0.9444416964857687 0.8576148692373655
0.0018022112968295 0.1219046900064080 0.8793725675527724
0.9961250236992137 0.0341562832451153 0.2144379162178212
0.9988422639343881 0.0553521873953827 0.1451629297302511
0.9962982164791329 0.9656260291834601 0.2143843266329175
-0.0008943231548002 0.9445343321942513 0.1450805211272521
0.0027271405190774 0.1020429415735400 0.0014145901236324
0.0027140941313648 0.8978032518677578 0.0012756588584477
0.0023065204440192 -0.0000974830134947 0.8993119236139577
0.0002280689615687 -0.0000259416941923 0.1035447936176636
0.1117387741020720 0.0000688885443251 0.0014359353749518
-0.0055772998838688 0.9319047872589716 0.2570702664909710
0.9987578677593123 0.0679165400439641 0.7456487360883973
0.9984713654264231 0.9320240412466286 0.7456112864077713
0.9976010552576622 0.2556400040216223 0.9334821576747717
0.9968177007285117 0.2555109048706737 0.0693978394288190
-0.0058969413620019 0.0678067063961208 0.2571739175308563
0.9972826662831048 0.7441622222694987 0.9334686237107822
0.9970740773025211 0.7443768835302400 0.0693879226003509
0.0009964786707367 0.1604995466460027 0.8407382444183652
-0.0013293498466372 0.1604640429410063 0.1620503569615059
0.0004414488732342 0.8393383964610521 0.8406387817213826
-0.0007590059701130 0.8394264002793270 0.1619282515578570
0.1301651553589043 0.0445972595492203 -0.0166700666964781
0.1304220635489645 0.9638646016803505 0.9699698339235414
```

V-porphyrin*NH2

```
   1.00000000000000
    20.00    0.00    0.00
     0.00   20.00    0.00
     0.00    0.00   20.00
   V    C    N    H
     1    20     5    14
Selective dynamics
Direct
  0.0242201316328928  0.0001660434857085  0.0001359663394993
  0.9978884573564129  0.2140997991680069  0.0345062949333393
  0.0027693621773389  0.1448613172635417  0.0557083737017385
 -0.0001324642756943  0.1224934249384031  0.1218204102809521
  0.9985567815317120  0.7860917377886342  0.9657912444724884
  0.0033067550816331  0.8553466207924910  0.9445794975309210
  0.0003157929208868  0.8777157204294016  0.8784699670302047
  0.9985552660009808  0.7860753609171754  0.0343170817642014
  0.0033183087484821  0.8553039309401246  0.0555762740667523
  0.9979797045857987  0.2141484450410454  0.9659754755267707
  0.0003653550017356  0.8775995582843346  0.1217072154027004
  0.9952633194970570  0.0345863043587466  0.7882449684803607
  0.0028824276712135  0.1449212253587334  0.9447046351032380
  0.9982224983914658  0.0557358252371957  0.8571738486290611
  0.9953271726227754  0.9657908349696209  0.7882190541412814
 -0.0016648574090720  0.9445927700698253  0.8571302039904932
  0.0000430120452870  0.1226019170647552  0.8785708298675347
  0.9950273946227309  0.0343929749964996  0.2120751646693723
  0.9981248171392847  0.0556112808456338  0.1431726952667121
 -0.0048102302456435  0.9655941619655877  0.2120419084198245
 -0.0016282864361219  0.9444554176059278  0.1431175906384995
  0.0073881410536895  0.1033371543242722  0.0001896180473052
  0.0078130451716780  0.8968930610263213  0.0000956482806622
  0.9993030030303761  0.0001459872518283  0.8988866754421564
  0.9993636490239262  0.0000541688947448  0.1014094954850104
  0.1171742945838136  0.0005827353627234  0.0001927570429084
 -0.0065361730903740  0.9319985417979310  0.2548340921368135
 -0.0063484684233634  0.0681713199811332  0.7454387515813349
 -0.0062300847230314  0.9322347883441069  0.7453846566993954
  0.9936511775921573  0.2567960700061465  0.9324059821552331
  0.9934817448067293  0.2567109421255758  0.0681067518714582
 -0.0068425856397928  0.0679293109814415  0.2549023922013005
  0.9942368271721371  0.7434696998678842  0.9321939147181054
  0.9942423526815805  0.7434266344396845  0.0678772831972633
 -0.0024741370236790  0.1608178124957399  0.8396104867048449
 -0.0028675124548581  0.1606674381223066  0.1607886701512123
 -0.0021768315207109  0.8395253342104724  0.8394775800057290
 -0.0022331869149901  0.8393849625837190  0.1606477102994434
  0.1459178235271813  0.0428804882058404  0.0000294547806220
  0.1463907024863536  0.9586098784567398  0.0002393789432569
```

Ti-porphyrin*NH2

```
   1.00000000000000
    20.00    0.00    0.00
     0.00   20.00    0.00
     0.00    0.00   20.00
   Ti   C    N    H
     1    20     5    14
Selective dynamics
Direct
  0.0293109195099176  0.0002249300272060  0.0012368435471463
  0.9973362151171339  0.2132134267841466  0.0340438020410677
  0.0024426247552804  0.1440361101271164  0.0551320246959788
  0.0003306362140345  0.1223209283193086  0.1215449568016152
  0.9960066095130679  0.7865726350460711  0.9653432186985307
  0.0004589888034826  0.8554174048837756  0.9433318441300240
 -0.0015505960996678  0.8774962449453989  0.8772369057260559
  0.9980502940431760  0.7870118132867449  0.0338922072325403
  0.0029897016567730  0.8561837590862645  0.0550113652476155
  0.9952527385918043  0.2136687107860588  0.9654909185155766
  0.0007076696789607  0.8778065861276541  0.1214406765396025
  0.9954495567639917  0.0346095616766623  0.7869637423849374
 -0.0001421842941068  0.1448556227525103  0.9434424323325086
  0.9982341647797068  0.0557182053356466  0.8558244727089844
  0.9955637496772518  0.9657263478666547  0.7869186807587308
 -0.0015652949859701  0.9445561487176067  0.8557574674365372
 -0.0020332359951918  0.1227629374069516  0.8773505130744824
 -0.0057211211007292  0.0345546609745766  0.2131397434606320
  0.9990914343873996  0.0557448884560368  0.1445054244801543
 -0.0056566640709083  0.9654769379012822  0.2131189562531546
 -0.0007739907910127  0.9443425286447230  0.1444799945538761
  0.0055929839661509  0.1028998110102907 -0.0012600874635728
  0.0060960984135316  0.8973722448090474 -0.0013572073738693
  0.9999073845209577  0.0001042717550347  0.8975241374851494
  0.0018499844123090  0.0000588237440235  0.1031570944600020
  0.1253380593817930  0.0006309916570579  0.0031276385607867
 -0.0083035865009800  0.9320160494578899  0.2559695723722817
 -0.0070160205624912  0.0681963521199438  0.7441945433118650
 -0.0067972420424921  0.9321770863833194  0.7441166954566533
  0.9901755780937425  0.2565922342205587  0.9323858313456652
  0.9943566397822846  0.2557188470855361  0.0679202866401958
 -0.0084367187870444  0.0679679428980572  0.2560178230304111
  0.9909846878243568  0.7436641504413050  0.9322100101405716
  0.9951097329760487  0.7444966788885894  0.0677600297855787
 -0.0049566641079888  0.1609828412555537  0.8384588545008586
 -0.0028332066907303  0.1612856895985615  0.1596975772316724
 -0.0043245125092520  0.8392666532681945  0.8383473118332174
 -0.0023653429046629  0.8387734622191579  0.1595324072906324
  0.1543565713455758  0.0425017721526100  0.0074167784965023
  0.1546678572344763  0.9588147078828768  0.0053245122756
```

S6.3 NO to hydroxylamine

Co-porphyrin*NOH

```
   1.00000000000000
    20.0000000000000000    0.0000000000000000    0.0000000000000000
     0.0000000000000000   20.0000000000000000    0.0000000000000000
     0.0000000000000000    0.0000000000000000   20.0000000000000000
   Co   C    N    H    O
     1    20     5    13     1
Selective dynamics
Direct
  0.0098791989278784  0.0010046751627080  0.0003489382960443
  0.9978620751486187  0.2130184796836569  0.0341618125542140
  0.9992687098571199  0.1437752306127104  0.0551892925634655
  0.9995279441817932  0.1234135170084575  0.1216689606239325
  0.0011435008548808  0.7902118913581840  0.9668753222807572
  0.0015989037449148  0.8593994216833687  0.9458070511779273
  0.0018174147165648  0.8797734389945798  0.8793237595053351
  0.0010643012499316  0.7903611412085207  0.0352352806831333
  0.0013968657560065  0.8596727142309816  0.0559200823602013
  0.9984071338272320  0.2128200067764410  0.9658085931963998
  0.0015851319545318  0.8802748916045465  0.1223564965694406
  0.9999967792317529  0.0351955378474894  0.7891929378136691
  0.0000121770933087  0.1434543772697038  0.9452070768615477
  0.0008739314676663  0.0561322826103451  0.8584754281331651
  0.0001073698038988  0.9668504928115497  0.7892538947665035
  0.0011377562244651  0.9460998454218355  0.8585684332206872
  0.0009522704122474  0.1226018497573508  0.8788683070981799
  0.9979640392273120  0.0364039656876405  0.2118520160093873
  0.0000094249281237  0.0570824173852543  0.1426551455771594
  0.9986125588275591  0.9679425767578771  0.2122412187474237
  0.0008566766388515  0.9467182326605521  0.1431980342662605
  0.0000052322398429  0.1014189442288400  0.0002715748075846
  0.0012444784586153  0.9018022594953456  0.0007119977134013
  0.0010152258627937  0.0011629733805727  0.9007131588485249
  0.0017928188551720  0.0017451792730395  0.1006080711526549
  0.1018721943170851  0.0034271020967120  0.9967041667059234
  0.9971509587688999  0.9341661499111393  0.2548797097110101
  0.9990148504129593  0.0692386860929535  0.7467483215106711
  0.9993221632666204  0.9327364949903002  0.7468693432234960
  0.9975274039543258  0.2551455444774297  0.9316100588700094
  0.9964523923813591  0.2555479597106911  0.0680816599243998
  0.9958944899999050  0.0705839377040067  0.2541540070024568
  0.0007169949348126  0.7476930197815083  0.9329136168045180
  0.0005307208314507  0.7480130493469715  0.0693991375861671
  0.0010842649082474  0.1610519547000008  0.8401414897132495
  0.9985708943053445  0.1621527220051468  0.1600977233512659
  0.0020788617496081  0.8410645624179645  0.8408620262948835
  0.0014578963011309  0.8416327181534673  0.1608955616652473
  0.1148595301849089  0.9660560840739965  0.0845015207430702
  0.1355189641922196  0.9626246716261621  0.0397287720666238
```

Cr-porphyrin*NOH

```
   1.00000000000000
    20.0000000000000000    0.0000000000000000    0.0000000000000000
     0.0000000000000000   20.0000000000000000    0.0000000000000000
     0.0000000000000000    0.0000000000000000   20.0000000000000000
   Cr   C    N    H    O
     1    20     5    13     1
Selective dynamics
Direct
  0.0198421017602101  0.9998343176289610  0.9998014212342142
  0.9967823677196445  0.2125085450890463  0.0341163965877766
  0.9997922450779704  0.1433191220181023  0.0553358488830252
  0.0005113689075923  0.1218480062838638  0.1218923104850740
  0.9972244502707391  0.7872573695286086  0.9656478065980564
  0.0000846775448013  0.8565016670852091  0.9445450850282591
  0.0005998519816733  0.8780018263212482  0.8779268005544687
  0.9972473025963432  0.7871398713798103  0.0341816529212436
  0.0001991287343748  0.8563322214708232  0.0553842167681087
  0.9968341726901048  0.2123738404820474  0.9655837118125786
  0.0008833569052153  0.8778102582582004  0.1219421244244686
  0.9985301785605837  0.0340588951245490  0.7866984646377123
  0.9997764459672339  0.1431190800951042  0.9445041480510802
  0.0012317148236119  0.0551702200712967  0.8559879400637251
  0.9985720255454303  0.9655088254445394  0.7867091054950552
  0.0013135089803447  0.9444176166721997  0.8559956688448991
  0.0004017815718045  0.1215976350784629  0.8778930672647742
  0.9976011511337576  0.0341823574887011  0.2128468759245181
  0.0019178346312362  0.0554131404817342  0.1438787857626275
  0.9977155279231369  0.9655145780831745  0.2128783795830637
  0.0020900134745792  0.9442577565028852  0.1439156355112697
  0.0012721342427216  0.1016163822060640  0.9999418769438156
  0.0015928023056887  0.8980293516299618  0.9999758164683977
  0.0032263991635274  0.9998030804404414  0.8974559647262129
  0.0055678712545560  0.9998301747013690  0.1022254224380573
  0.1070561675104685  0.0000300452935375 -0.0002775692847942
  0.9942714018275748  0.9318963176166937  0.2555337498916886
  0.9963122929179965  0.0677498860871830  0.7440109722258368
  0.9964023165600242  0.9318023851580199  0.7440231388228452
  0.9948119463546358  0.2549928074834952  0.9317968667264614
  0.9947985442014680  0.2552358700146994  0.0677675517966004
  0.9940517079693517  0.0678192089118915  0.2554850408037432
  0.9951976341064355  0.7446298535591243  0.9318747598305293
  0.9953224768204122  0.7444196289026818  0.0678467625144485
  0.9989950974510715  0.1603485112611783  0.8394350751203469
  0.9982962798650825  0.1605364040033451  0.1603732992022929
  0.9991996386507013  0.8392320229151471  0.8394863066950888
  0.9987671715432918  0.8391196262331965  0.1604235263253663
  0.1247413341927201  0.9975104118824509  0.0943578794163683
  0.1511500762618521  0.9987018811109455  0.0526981129007152
```

Cu-porphyrin*NOH

```
   1.00000000000000
    20.0000000000000000    0.0000000000000000    0.0000000000000000
     0.0000000000000000   20.0000000000000000    0.0000000000000000
     0.0000000000000000    0.0000000000000000   20.0000000000000000
   Cu   C    N    H    O
     1    20     5    13     1
Selective dynamics
Direct
  0.9981267620224594  0.0001406200426961  0.9978226421841629
  0.9987703389610418  0.2125727609926580  0.0329057632486395
  0.9991450130176270  0.1431783618226538  0.0539043643271484
  0.0000873869241032  0.1219206188730708  0.1204954569265043
  0.9969404133699892  0.7876995941032955  0.9641061513654987
  0.9970210929890061  0.8571002316322860  0.9431257790176379
  0.9966049912130828  0.8784755516482313  0.8765860480298807
  0.9972357799032017  0.7876125277331006  0.0325895625173835
  0.9977102743771825  0.8569636048235779  0.0537256060001026
  0.9976900588651721  0.2126134387361186  0.9644315160409936
  0.9982216979442774  0.8780727009981325  0.1203703689815842
  0.9958045110557623  0.0345440877964564  0.7856835213197136
  0.9973001025521504  0.1432543243627937  0.9433191229177397
  0.9963094853921047  0.0555490007831523  0.8550847229168292
  0.9959480839839356  0.9660459776817869  0.7856413202895516
  0.9964834919215589  0.9449567687504217  0.8550210579646529
  0.9964189625588136  0.1219953046399561  0.8767472894143986
  0.9974840726600892  0.0342913828876287  0.2112375645947285
  0.0006592954622611  0.0555118222546147  0.1422029967632673
  0.9967714347610011  0.9656639735331726  0.2112142235041774
  0.9995605292936502  0.9444360163880551  0.1421777451225189
  0.9980364956104163  0.1016706059275424  0.9985673442144569
  0.9974007431481801  0.8985813344324115  0.9984514301809904
  0.9967605271646258  0.0002259292031715  0.8964821599159218
  0.0031839162088956  0.9999589772793670  0.1004412461299918
  0.1832092513941776  0.9993624389412388  0.0244204593040673
  0.9942526006807969  0.9319992263083799  0.2539037219899103
  0.9954686587581241  0.0683675876007961  0.7430476113512637
  0.9957452753115180  0.9322619834819672  0.7429733209404250
  0.9972135039430147  0.2552968674674016  0.9306707943655387
  0.9994485870148262  0.2552104761024772  0.0667216151037894
  0.9956181982494068  0.0679717813795662  0.2539473752179354
  0.9966594221467359  0.7450847720593141  0.9302626699329406
  0.9973872751863861  0.7449234348833494  0.0663591022101657
  0.9959482566362264  0.1607443448492073  0.8382971810404665
  0.9995202707339575  0.1606467406762589  0.1589631859125732
  0.9963498732409728  0.8397985141947515  0.8380585835992391
  0.9969887268293217  0.8392843963460478  0.1587672812456943
  0.1104877052347606  0.9946902846383437  0.0867011012992331
  0.1602114332791442  0.9907926337445644  0.0866709925982812
```

Fe-porphyrin*NOH

```
   1.00000000000000
    20.0000000000000000    0.0000000000000000    0.0000000000000000
     0.0000000000000000   20.0000000000000000    0.0000000000000000
     0.0000000000000000    0.0000000000000000   20.0000000000000000
   Fe   C    N    H    O
     1    20     5    13     1
Selective dynamics
Direct
  0.0155414166655600  0.9998094365412529  0.9991174071398801
  0.9979702813548117  0.2107264517307177  0.0347370136771197
  0.0000465778194860  0.1416572629146567  0.0556982643349522
  0.0015128617329773  0.1212402198929799  0.1223147553718746
  0.9981054790209871  0.7890085446385247  0.9662525082220710
  0.0000095290863056  0.8580891207180463  0.9452892444392840
  0.0012864344333260  0.8786024848762155  0.8786009911092832
  0.9980927256120921  0.7889438973585566  0.0347564061466029
  0.0000954074834527  0.8580033732628952  0.0557325788385464
  0.9978908257806735  0.2106795195808185  0.9662377999847308
  0.0014077571787459  0.8784058100691660  0.1223621088842033
  0.0001211275194324  0.0339943881462257  0.7878179951058855
  0.9997943659049979  0.1415904131743166  0.9452669434633313
  0.0020739844330229  0.0549062209574883  0.8572625253115135
  0.0001863132584153  0.9656731153795531  0.7878251343205551
  0.0021871248755868  0.9447778417768021  0.8572702203192665
  0.0010705573107180  0.1210885078010393  0.8785790111729119
  0.9992178969327566  0.0340863695542662  0.2129343029598821
  0.0030518199380993  0.0550422962636508  0.1438196518872465
  0.9991461915121589  0.9656107449718445  0.2129685709517788
  0.0029347839564702  0.9446041135042277  0.1438662569054503
  0.0002073030686991  0.0994330468060994  0.0003907492620067
  0.0003420293981725  0.9002488250110100  0.0004238648359487
  0.0035201447345829  0.9998514276686243  0.8992764057185770
  0.0065701399215973  0.9998123291105022  0.1015377151460154
  0.1001405192563345 -0.0000178993978895  0.9921998665422538
  0.9957690484004706  0.9317657829257805  0.2554486898349559
  0.9981942133142391  0.0679279826120791  0.7453137522267134
  0.9983311805866341  0.9317270967759420  0.7453266875921200
  0.9964589639722247  0.2532017160298345  0.9323086505705466
  0.9967443228919003  0.2532593981253669  0.0686563873095156
  0.9959145054150986  0.0679691371963024  0.2553906393720581
  0.9967358980374644  0.7464890336244947  0.9323169144906603
  0.9968160444783780  0.7464041599382962  0.0686640277215391
  0.0004373742051929  0.1600335642905232  0.8403353935726167
  0.0001400371339100  0.1602146031199233  0.1605149698857656
  0.0006896032027191  0.8396523172024886  0.8403613639606302
 -0.0000285243878549  0.8394234134442077  0.1605511924719030
  0.1110446817802580  0.9972923695977299  0.0871970761311719
  0.1404135527798559  0.9982425628054439  0.0471759628086390
```

Mn-porphyrin*NOH

```
   1.00000000000000
    20.0000000000000000    0.0000000000000000    0.0000000000000000
     0.0000000000000000   20.0000000000000000    0.0000000000000000
     0.0000000000000000    0.0000000000000000   20.0000000000000000
   Mn   C    N    H    O
     1    20     5    13     1
Selective dynamics
Direct
  0.0190910159520146  0.9998598145741817  0.9997422029312810
  0.9970005025741213  0.2118522463680609  0.0339652814453000
 -0.0002505209718913  0.1426247270174151  0.0550342400753659
  0.0007606495809878  0.1216842693244170  0.1215857326668352
  0.9973584657532680  0.7879863740708068  0.9655695437240881
  0.9998352005873893  0.8572867999320728  0.9446880327334171
  0.0004699917112939  0.8782630123688413  0.8780996695252695
  0.9976117463661660  0.7878287606855305  0.0340335065442692
  0.0002400959382740  0.8570653619330035  0.0550736715164792
  0.9968983806812888  0.2116681584172279  0.9655007482854375
  0.0012065453453570  0.8780036084493877  0.1216299731039084
  0.9992024311119757  0.0340475351158586  0.7872732989449240
  0.9995564263532241  0.1423557528349224  0.9446561060951633
  0.0012646750199825  0.0549247226782673  0.8565941335398166
  0.9991594919251504  0.9655353700577179  0.7872705578165639
  0.0012395390081117  0.9446590353269129  0.8565863130407499
  0.0003868421253428  0.1213332542189505  0.8780833264647712
  0.9988042999330546  0.0341887479133613  0.2120796026347402
  0.0019785491340402  0.0552464090158213  0.1430865416349146
  0.9989095520125157  0.9655408888780295  0.2121083838169549
  0.0021562629322155  0.9444513052295430  0.1431274837920281
  0.0008741845128540  0.1004932627575308  0.9999053554581527
  0.0011802384582086  0.8991900989103896  0.9999230711865070
  0.0027931191039576  0.9997909129285442  0.8984536191410342
  0.0047936574507764  0.9998409805669287  0.1009201773229901
  0.1037498661305187 -0.0000036810737818  0.0010128588417506
  0.9963981625983406  0.9318064897419513  0.2547564679013866
  0.9975570064531718  0.0678762870176619  0.7446625859071166
  0.9974712749379671  0.9317115604458190  0.7446574902572262
  0.9950279404056732  0.2541607359421893  0.9315375372875349
```

```
 0.9953526858844228  0.2545181176776416  0.0677170923707248
 0.9962106133799142  0.0679435786207742  0.2547067062920252
 0.9954688771547798  0.7454762275793837  0.9316297297918198
 0.9960808362363521  0.7451751935321017  0.0678063066576026
 0.9992741574799368  0.1601025836567575  0.8396499945904256
 0.9992444376861473  0.1604002286962044  0.1600683216926155
 0.9993421856657468  0.8394664333101692  0.8396935240701268
-0.0001578136606747  0.8392776189629421  0.1601066280449361
 0.1197530237409010  0.9972938701200932  0.0951911921334824
 0.1468899033070821  0.9985443461963858  0.0539129907202854
```

Ni-porphyrin*NOH

```
   1.00000000000000
    20.0000000000000000    0.0000000000000000    0.0000000000000000
     0.0000000000000000   20.0000000000000000    0.0000000000000000
     0.0000000000000000    0.0000000000000000   20.0000000000000000
   Ni   C    N    H    O
     1   20     5   13     1
Selective dynamics
Direct
 0.9973981248994260  0.9997226705789297  0.9999213323338367
 0.9970866063908768  0.2103755968739856  0.0340829971803341
 0.9969873689544442  0.1412245582569605  0.0549141652250253
 0.9962113106475167  0.1210691040616255  0.1212671087875756
 0.9973666080396535  0.7890649944177295  0.9657394174502848
 0.9979231913618519  0.8582181827387958  0.9449094856758566
 0.9988537860747849  0.8783538492226880  0.8785485607726869
 0.9964249794877951  0.7890735689635442  0.0340561395534542
 0.9964067063108225  0.8582268758800168  0.0548873758208464
 0.9977042392773161  0.2103876406083773  0.9657667511343848
 0.9954820546233187  0.8783645369933603  0.1212468027020368
 0.0000654015818661  0.0338866275190553  0.7892595370601565
 0.9980408889301184  0.1412319293377872  0.9449268245171070
 0.9991640899714245  0.0547259131322620  0.8584096093009339
 0.0001445479463533  0.9655597256691898  0.7892692029894121
 0.9992689363675088  0.9447218287175071  0.8584161202530665
 0.9987461744024673  0.1210935204756246  0.8785635590720804
 0.9947176881895149  0.0338677815171049  0.2105568264427070
 0.9958644407007073  0.0547047805309229  0.1414082557351306
 0.9944478589096805  0.9655527723211538  0.2105408919291973
 0.9954706895521372  0.9447196088352173  0.1413935932120689
 0.9975668489603653  0.0987209712780679  0.9999218066238166
 0.9972877112427844  0.9007273707476056  0.9998999108310354
 0.9986973839588046  0.9997235865533943  0.9009195091110391
 0.9963440116386483  0.9997136903561065  0.0989070184591866
 0.2007383630788347  0.0214513208878470  0.0416367785716452
 0.9937203973543144  0.9313766575336975  0.2528596631145259
 0.0006709472188108  0.0680877342370552  0.7469384834786372
 0.0007424169054473  0.9313831250498912  0.7469359294327985
 0.9980192886524893  0.2527181503475928  0.9315894166736709
 0.9968429867599023  0.2526927543158035  0.0682692823189908
 0.9943218570499689  0.0680552442530414  0.2528775174670123
 0.9976615116496761  0.7467365670600243  0.9315551555120074
 0.9958008581984310  0.7467434806210916  0.0682240009488593
 0.9991099272143835  0.1596768293389596  0.8400058604330994
 0.9958390076361272  0.1596256131560133  0.1598390399027084
 0.9992400683269337  0.8397821015959613  0.8399780544491571
 0.9947266052763265  0.8398125125151170  0.1598217602159200
 0.1185518894716426  0.0010099159931401 -0.0039891396227309
 0.1678767267864829  0.9987373075077488  0.9880253949304333
```

Ti-porphyrin*NOH

```
   1.00000000000000
    20.0000000000000000    0.0000000000000000    0.0000000000000000
     0.0000000000000000   20.0000000000000000    0.0000000000000000
     0.0000000000000000    0.0000000000000000   20.0000000000000000
   Ti   C    N    H    O
     1   20     5   13     1
Selective dynamics
Direct
 0.0323961159347016  0.9998888395149644  0.9984907702106641
 0.9964578192728247  0.2138647632092398  0.0334178239091665
 0.0023670613006313  0.1447025359575895  0.0546582122648945
 0.0021400506975178  0.1221985939316687  0.1211269811267151
 0.9955383567326794  0.7859609833077651  0.9647873705893503
 0.0010207083791983  0.8551248477070075  0.9434430699828690
 0.9993140023640473  0.8776382337497368  0.8769897094377747
 0.9961627801553253  0.7859444720180951  0.0333800858697542
 0.0021013268957415  0.8550949345056442  0.0546497465303182
 0.9957525130027236  0.2138662489291492  0.9648271332437371
 0.0018663293487897  0.8775839357606525  0.1211258346157808
 0.9939339452160582  0.0342572267262569  0.7857035303078638
 0.0011753251118674  0.1447072254110838  0.9434553047834618
 0.9994389946248103  0.0555740260383941  0.8547631357034153
 0.9939141972926383  0.9655986566695374  0.7856993702839861
 0.9994015913751884  0.9442758238256568  0.8547604426582701
 0.9994234830167363  0.1222096463000376  0.8769982141899493
 0.9986650436783294  0.0342409635088458  0.2125213301435153
 0.0029023506135163  0.0555451305312751  0.1433732890415069
 0.9985597668046775  0.9655578841954444  0.2125232638561169
 0.0027478986610111  0.9442347086757136  0.1433793032645952
 0.0058404900951985  0.1036997151546476  0.9990031368033733
 0.0056647701839464  0.8961116620116147  0.9990116825908746
 0.0027807163968236  0.9999246950928058  0.8958453012843741
 0.0056235063775394  0.9998820546460983  0.1022471685598543
 0.1195403603012774  0.9997676699136137  0.9970211991484825
 0.9951834664200965  0.9320756856700310  0.2553003452120396
 0.9899645551822396  0.0677623263682640  0.7430003505818256
 0.9899125205348287  0.9320982163984965  0.7429952964761383
 0.9909801265579474  0.2565165676779267  0.9313499204152246
 0.9923482545677910  0.2565160034332475  0.0669814267724254
 0.9953848120484056  0.0677384395262327  0.2552935232610494
 0.9907759640457816  0.7433233703452057  0.9312931227499275
 0.9919993908183616  0.7432832814619149  0.0669261403867973
 0.9959798344563640  0.1606711492736890  0.8383648287447584
 0.9992284277551847  0.1606478176119432  0.1598229055261930
 0.9958488492982670  0.8391855250775023  0.8383495568124186
 0.9988521606443879  0.8391331196540779  0.1598127809347386
 0.2058624678560314  0.9967534140729895  0.0351812700595585
 0.1876341659804870 -0.0000393938640225  0.9897261216662403
```

V-porphyrin*NOH

```
   1.00000000000000
    20.0000000000000000    0.0000000000000000    0.0000000000000000
     0.0000000000000000   20.0000000000000000    0.0000000000000000
     0.0000000000000000    0.0000000000000000   20.0000000000000000
   V    C    N    H    O
     1   20     5   13     1
Selective dynamics
Direct
 0.0282854216235704  0.9999279367887572  0.9986017526503517
 0.9979425761963401  0.2132973448543668  0.0333493795748365
 0.0018326767255991  0.1440147343949099  0.0544197018344200
 0.0018025601834209  0.1220138027852705  0.1208918795366486
 0.9971565207657856  0.7866054480493416  0.9647812824572324
 0.0006971967604215  0.8558864168114171  0.9436497470135583
 0.9994424113690735  0.8779129825454967  0.8771905943756068
 0.9977483369478767  0.7866062673648093  0.0333465031985477
 0.0016938153927595  0.8558867072675734  0.0544117280242312
 0.9974232830762888  0.2132908340798554  0.9647863588061971
 0.0017503840074149  0.8778981711428232  0.1208855972724694
 0.9953774852855793  0.0342526115015274  0.7863056517437966
 0.0009548101702544  0.1440051686730354  0.9436630007891011
 0.9993927551118956  0.0553805176997817  0.8554801079158042
 0.9952987245366908  0.9656570049129500  0.7862992978890887
 0.9992609256685129  0.9445133100194616  0.8554719841605701
 0.9997119511408481  0.1219790610522903  0.8772039864332165
 0.9994305693860449  0.0342711510214601  0.2118827474696660
 0.0021178896846407  0.0553999755545834  0.1426516781862666
 0.9994230083197073  0.9656460570558453  0.2118824284316576
 0.0021019440166332  0.9445141824124789  0.1426510909118223
 0.0039620755668769  0.1025262374432150  0.9990086179939462
 0.0037828383193325  0.8973666145972727  0.9989941944121492
 0.0016521676514861  0.9999433160698314  0.8970139660069359
 0.0037617799897051  0.9999563944785463  0.1010848132251246
 0.1121649875452336  0.9997630980896413  0.9972044338840188
 0.9972754613843502  0.9320198193446867  0.2546372207489026
 0.9925280748972305  0.0679027813977176  0.7436153050529555
 0.9923669249025564  0.9320208612004116  0.7436031669579024
 0.9942386027676897  0.2559681513637466  0.9311519613349372
 0.9952483604989797  0.2559832273950924  0.0670177997411640
 0.9972841287829769  0.0679006591766644  0.2546341050193551
 0.9939108100804451  0.7439256549018695  0.9311556989600730
 0.9950779433409738  0.7439212276695213  0.0670187753067601
 0.9975455234829098  0.1604699937613633  0.8385118428057148
 0.0000944057010559  0.1605120870164403  0.1596044897073131
 0.9971997130910273  0.8394272899777399  0.8384985055447609
 0.0000403809587241  0.8394015266646989  0.1596005784132766
 0.1980332356007561  0.9951600888425717  0.0353736061520300
 0.1796718390683073 -0.0000077153790565  0.9900644200575833
```

Co-porphyrin*NHO

```
   1.00000000000000
    20.0000000000000000    0.0000000000000000    0.0000000000000000
     0.0000000000000000   20.0000000000000000    0.0000000000000000
     0.0000000000000000    0.0000000000000000   20.0000000000000000
   Co   C    N    H    O
     1   20     5   13     1
Selective dynamics
Direct
 0.0096733842063591  0.0010784259037533  0.0001369641829601
 0.9984823636828710  0.2121872522222386  0.0351850323623028
 0.9992847917897607  0.1428345562145397  0.0559108598285761
 0.9983758199117871  0.1222199015390845  0.1223000831411630
 0.0012951640922386  0.7894918408240510  0.9664221802444202
 0.0018967116292471  0.8586538131753658  0.9454192877046420
 0.0007649607514119  0.8793843355383384  0.8790303959649589
 0.0023092175428715  0.7895921474805144  0.0348283594814859
 0.0033202727347151  0.8588286089364640  0.0556735423706760
 0.0000827953684399  0.2123545703987521  0.9668676852654865
 0.0029726253629236  0.8794322439481331  0.1221274093684167
 0.0010280317956057  0.0353533489710088  0.7896526576355462
 0.0016393951770034  0.1430907967863490  0.9459349212301035
 0.0019464228618178  0.0560935865284851  0.8588913129638257
-0.0002026877280695  0.9669731663476258  0.7894615737553319
 0.0002988694525581  0.9458624298880872  0.8585994301704075
 0.0028755219476210  0.1224888647544019  0.8795711688789910
 0.9972776774853025  0.0348931070145624  0.2121875346888189
 0.9985327792336008  0.0558038171169073  0.1429487448148941
 0.9988240572406596  0.9665218818349922  0.2121665936808446
 0.0008587477864816  0.9457884628310108  0.1428826113170787
 0.0009053419720313  0.1007782618048263  0.0008056241217900
 0.0031478774343060  0.9010632716459503  0.0004816564331424
 0.0008380932723982  0.0009164440582400  0.9009833467394401
 0.0001572946572415  0.0008477300705977  0.1007078739943303
 0.1049297647187392  0.9986392751137120  0.9966857560697520
 0.9984292118890730  0.9324198775989780  0.2545721545762613
 0.0011027052070629  0.0695113400171496  0.7473031471362187
 0.9987747147602598  0.9330354549142008  0.7469313263861537
-0.0000556619283946  0.2548377960842815  0.9328701294889016
 0.9968127012457793  0.2545027774619751  0.0693554855704726
 0.9954299437766965  0.0689038757045353  0.2546327026040451
-0.0000677336886694  0.7469893695730156  0.9324798788776502
 0.0018422402647020  0.7471962981382255  0.0689442332963399
 0.0036976913495138  0.1610490629913764  0.8409700943163840
 0.9972193228120658  0.1608180141360999  0.1608570720226499
-0.0000116345579150  0.8408967700936194  0.8403495329312132
 0.0032492798968679  0.8407617241798985  0.1606366180305699
 0.1215082105305837  0.9492133676211413  0.9901004601274298
 0.1417382140623810  0.0401131305375247  0.9673345582263290
```

Cr-porphyrin*NHO

```
   1.00000000000000
    20.0000000000000000    0.0000000000000000    0.0000000000000000
     0.0000000000000000   20.0000000000000000    0.0000000000000000
     0.0000000000000000    0.0000000000000000   20.0000000000000000
   Cr   C    N    H    O
     1   20     5   13     1
Selective dynamics
Direct
 0.0157917733078081  0.0008631430940891  0.9993923097324152
-0.0009063574462822  0.2132944169693495  0.0346267286405514
 0.0003020323408372  0.1440407617819509  0.0556226850705979
 0.9993342372727505  0.1221335176828687  0.1220486422376584
-0.0001394884670363  0.7876559444584743  0.9647140242576802
 0.0015730110233741  0.8568863350508691  0.9437372386154802
 0.0007296745225234  0.8788311135956317  0.8773242042453557
 0.0000050470575307  0.7874639159009674  0.0333225468221474
 0.0016972632799867  0.8565701630466575  0.0547383194952266
 0.0001419631800578  0.2134377478483340  0.9660596216513400
 0.0009764626455882  0.8781470891219312  0.1213302020033547
 0.9994598550316289  0.0353682484638602  0.7869966235131389
 0.0018412303525274  0.1442815399024147  0.9447361998660989
 0.0015608797687354  0.0563039844857691  0.8562870253867182
 0.9986978230903074  0.9668186655272761  0.7867338868149458
 0.0005438910427951  0.9453947920379018  0.8558711259477646
 0.0021874121871797  0.1227148926215403  0.8782033012649204
 0.9962881963920267  0.0341840034225456  0.2126003190815636
 0.9994240199284345  0.0555887549344134  0.1435197449398758
 0.9967538025018906  0.9656082058529580  0.2123988784693528
 0.0001147282802847  0.9446043761911647  0.1431931709278232
 0.0018543883387731  0.1025246684658317  0.0000701862102589
 0.0029402572072845  0.8984378795417193  0.9994114839194380
 0.0019352609795209  0.0007298208240482  0.8977276481025765
 0.0017635370161128  0.0001860295138146  0.1016619231882850
 0.1088440306500328  0.0010041374290833  0.9986936000066376
 0.9944835828830868  0.9317824516167704  0.2549934811847656
 0.9984079287470103  0.0692559562300011  0.7444188727258685
 0.9969075219355601  0.9332732039664944  0.7439142731312030
-0.0004855858898702  0.2562234453894171  0.9324211559323520
 0.9974672474557958  0.2559305699531613  0.0684099585771197
 0.9936028196811015  0.0677574082820862  0.2553615788154719
 0.9982474453733460  0.7450372952416551  0.9308924688934993
 0.9985133238772914  0.7446163080552635  0.0668712769977577
 0.0020562117557014  0.1614273487122670  0.8396883853838150
 0.9976692412808280  0.1606045618039847  0.1607682730241371
-0.0005348922860506  0.8403537684139917  0.8385934410007461
-0.0002859784638187  0.8394381435528144  0.1598337810985807
 0.1315025059337566  0.9645437222275746  0.0285079477907781
 0.1499181962315484  0.0381026687890499  0.9675034650327317
```

Cu-porphyrin*NHO

```
   1.00000000000000
    20.0000000000000000    0.0000000000000000    0.0000000000000000
     0.0000000000000000   20.0000000000000000    0.0000000000000000
     0.0000000000000000    0.0000000000000000   20.0000000000000000
   Cu   C    N    H    O
     1   20     5   13     1
Selective dynamics
Direct
-0.0035485167107660  0.9986778625364467  0.0001643833615011
 0.9943734283736565  0.2115922197118590  0.0344188849569435
-0.0042082697323632  0.1422802373708010  0.0555082169746833
-0.0039358074943629  0.1207559905931834  0.1220338080472492
 0.9964939298374469  0.7861821580320368  0.9659547631163042
-0.0037330077335673  0.8555895733407153  0.9449094910247877
-0.0038332615578504  0.8770529172051970  0.8783884488481999
 0.9965856448746083  0.7861894945592849  0.0344468954763812
-0.0035530496860772  0.8556024917941001  0.0554809938743318
 0.9940310111558776  0.2115849472562326  0.9658625890065770
-0.0033560739345136  0.8770828720859212  0.1219970850202589
 0.9953898193451907  0.0330842245814556  0.7874649926655428
-0.0047482898470165  0.1422642173488460  0.9447784249134523
 0.9953943196336268  0.0541609022485228  0.8568591975435321
 0.9958054615707279  0.9645830305030207  0.7875141044741497
-0.0040134813835906  0.9435778093047943  0.8569137310777080
-0.0050057127820244  0.1207132203930031  0.8782562038995676
 0.9966897842396965  0.0331583779459058  0.2128546214295608
 0.9964927582341642  0.0542087920695847  0.1434570950717389
 0.9968553134535918  0.9646543895499986  0.2128347046462148
-0.0032723167862639  0.9436138816889971  0.1434456982519333
-0.0033742902336056  0.1007085731297334  0.0001462442012970
-0.0037558245220925  0.8970615443548030  0.0001942643361987
 0.9958049247333415 -0.0011211381487444  0.8983960878795887
 0.9965831360712880 -0.0010978062925495  0.1019376129902839
 0.1880543777091538  0.0714782321824853  0.9894342082161245
-0.0029794997886751  0.9308911588948870  0.2555256965905930
-0.0048586204902148  0.0668225691875834  0.7447487508171601
-0.0040443016642603  0.9308024276102608  0.7448327816512478
 0.9929183634504165  0.2543126736412836  0.9321523057428851
 0.9935880021758150  0.2543302794837448  0.0681254940648948
-0.0033272973617603  0.0669265554634003  0.2555508883010232
-0.0033798631438828  0.7434941054463929  0.9321859507027136
-0.0031657204472174  0.7435042921055312  0.0682160451029271
-0.0058407066902073  0.1592918012187574  0.8396315713179054
-0.0044305503709393  0.1593520666680966  0.1606536325527025
-0.0037894191818689  0.8384364583949717  0.8397872468318707
-0.0032714353400095  0.8384797821063060  0.1606045772716730
 0.1421018353639247  0.0989013708828659  0.9939671944962580
 0.1753477066605843  0.0113364435502884  0.9900151132520356
```

Fe-porphyrin*NHO

```
   1.00000000000000
    20.0000000000000000    0.0000000000000000    0.0000000000000000
     0.0000000000000000   20.0000000000000000    0.0000000000000000
     0.0000000000000000    0.0000000000000000   20.0000000000000000
   Fe   C    N    H    O
     1   20     5   13     1
Selective dynamics
Direct
 0.0124263101803725  0.0009775730213381  0.9991465166686146
 0.0002302209390662  0.2121418003392764  0.0339473263215156
 0.0012496333963630  0.1428182667005090  0.0546530357944092
 0.0012648693765975  0.1218290244859169  0.1210457164204547
 0.0004698735806784  0.7889188166329353  0.9650411384228998
 0.0018208965517015  0.8581923945505333  0.9443059483969227
 0.0016940320187036  0.8794003582834430  0.8779770004638464
 0.0004449321524703  0.7886596345217445  0.0334825496961199
 0.0016317345986022  0.8578410568130935  0.0546252651546637
 0.0004986963690244  0.2124206826097112  0.9655457849315311
 0.0016687045690112  0.8786523054566411  0.1211108409320688
 0.9995643668318862  0.0351882366599957  0.7878925392535360
 0.0014849626769049  0.1432343752675403  0.9444161696100143
 0.0010166725645393  0.0561305626583054  0.8571152730187418
 0.9994310181923539  0.9667789236905523  0.7879769395259528
 0.0009845685471017  0.9458693074430792  0.8572161218139407
 0.0016234247360617  0.1225218936418328  0.8779815906379880
 0.9988645401265116  0.0344836382969194  0.2112051317420439
 0.0008705231347218  0.0554220687991039  0.1420192881051251
 0.9989710242227405  0.9660370983176871  0.2112690046599976
 0.0008874401738151  0.9450942252385111  0.1420551014574418
 0.0015859184632062  0.1007726546165872  0.9993597694236930
 0.0022375192370884  0.9002967223745514  0.9996129551729681
 0.0013501910466020  0.0010560804205135  0.8994236389447645
 0.0016627707498111  0.0002357602934003  0.0997592802076595
 0.0998092763166675  0.0002404215692344  0.9998237333537936
 0.9973911483198059  0.9321222173737880  0.2537979300258514
 0.9984687032996128  0.0691093302819435  0.7453606163352111
 0.9983149273034833  0.9328021993910892  0.7454795680244133
 0.9997402227694714  0.2550526431940121  0.9317410312781569
 0.9993104486045774  0.2545452948198757  0.0680478546058751
 0.9973001029179350  0.0684227640635301  0.2537197526460607
 0.9993369336695529  0.7464869330384136  0.9309807501693466
 0.9991689444268317  0.7460137533029775  0.0672625820321932
 0.0018021542248617  0.1611550953102162  0.8394233757862968
 0.0008312191006746  0.1604253618507198  0.1596500464540277
 0.0013963650193730  0.8409360063117780  0.8392400997848042
 0.0014216820618823  0.8400158535576434  0.1596726835337514
 0.1211724238304802  0.9656225371170908  0.0329664443124846
 0.1417851036987863  0.0334971276839628  0.9678496048808102
```

Mn-porphyrin*NHO

```
   1.00000000000000
    20.0000000000000000    0.0000000000000000    0.0000000000000000
     0.0000000000000000   20.0000000000000000    0.0000000000000000
     0.0000000000000000    0.0000000000000000   20.0000000000000000
   Mn   C    N    H    O
     1    20     5    13     1
Selective dynamics
Direct
  0.0123367174100927  0.0012827733020155  0.9989587967679618
  0.9998308789584325  0.2130267301641492  0.0340478919033723
  0.0004119799710163  0.1437703704981139  0.0551653310854259
  0.9986347760568175  0.1223801840385233  0.1213742710095501
  0.0027193423033123  0.7885235060602115  0.9650304418777192
  0.0027273786346576  0.8577632859283705  0.9439876443604581
  0.0005458251687850  0.8791020073077103  0.8777101557357021
  0.0037890741925556  0.7882828008740072  0.0334469195083212
  0.0043310959774573  0.8574996021876712  0.0547470450883956
  0.0016064244617233  0.2131342673011188  0.9656962586328052
  0.0037814480500082  0.8786420698378541  0.1211492717877803
  0.9978564352657416  0.0354658137255148  0.7877227310109352
  0.0027966757048584  0.1438632150757300  0.9445197205457634
  0.0005781079814136  0.0563798128701431  0.8567994803016549
  0.9965672606049379  0.9669076385145448  0.7876147715402397
  0.9989530605937156  0.9455758597965918  0.8565227532787442
  0.0023954423059472  0.1228000543492075  0.8781868685757499
  0.9962008062410419  0.0345868542585279  0.2113926464653388
  0.9981517767387829  0.0558225637451419  0.1424347296581437
  0.9979832970282712  0.9660474571791500  0.2114308170390510
  0.0008390016586364  0.9450797391823588  0.1423715039826020
  0.0023981433303045  0.1017566522702382  0.9998305367237810
  0.0038486414035384  0.8997650726030550  0.9995235658676632
  0.0004822516319666  0.0008861089017490  0.8986453233422800
  0.0001765690324124  0.0004875371870904  0.1003402712386899
  0.1023967223212058  0.9984427403272143  0.0000374311589101
  0.9969819397163674  0.9321561665790912  0.2539861349635738
  0.9968323823852347  0.0694020790439932  0.7452067013729505
  0.9944768735611945  0.9332206812773379  0.7449228609677780
  0.0015638160964064  0.2557218594879779  0.9318312906369108
  0.9981812807341875  0.2555750192335629  0.0679409305520280
  0.9936890375133337  0.0683436290963167  0.2540014070506239
  0.0014915435023138  0.7460013272089338  0.9310866282023884
  0.0033314331866667  0.7455996502497038  0.0671897494602759
  0.0024874652100667  0.1614300197372612  0.8396177338333493
  0.9972187298589552  0.1608136455302415  0.1601224153470374
  0.9995936092244866  0.8405534328789477  0.8390560545763234
  0.0044987209832998  0.8400632075553570  0.1597628999260438
  0.1238356533505284  0.9571356982560956  0.0239884182231309
  0.1446628816492843  0.0381298663791743  0.9757995964005727
```

Ni-porphyrin*NHO

```
   1.00000000000000
    20.0000000000000000    0.0000000000000000    0.0000000000000000
     0.0000000000000000   20.0000000000000000    0.0000000000000000
     0.0000000000000000    0.0000000000000000   20.0000000000000000
   Ni   C    N    H    O
     1    20     5    13     1
Selective dynamics
Direct
  0.0017369836664738  0.0007547875949993  0.0016744362077976
  0.9963209500700878  0.2114447345749237  0.0359606761463421
 -0.0037885976211741  0.1421997361245488  0.0567356448860188
 -0.0053369176920380  0.1219633162238709  0.1230865602876733
  0.9993787025365751  0.7897798829212526  0.9674548055086817
 -0.0009252543135477  0.8589323647963428  0.9465311980426498
 -0.0015661172028221  0.8792023381731764  0.8801213045862553
  0.0007292832790242  0.7898729952941999  0.0358054828273222
  0.0009751841307906  0.8590793018055902  0.0565699413292548
  0.9992399248770736  0.2114843940410920  0.9677083611977846
  0.0010265824239604  0.8793585749667152  0.1229625171983014
  0.0024348641424935  0.0349615413573367  0.7910035259061436
  0.0005652490750798  0.1422569819080394  0.9469060929188455
  0.0022625732940604  0.0556879772994952  0.8602016675698791
  0.0004485991785949  0.9666389636747632  0.7908410331934467
 -0.0005746066587728  0.9456245060302303  0.8599581318704947
  0.0028577232385956  0.1220407327253644  0.8805524932127026
  0.9952596527300812  0.0347256117954388  0.2125026307487437
  0.9957680737219493  0.0555793929729493  0.1432878297318765
  0.9975521075034532  0.9664453630927348  0.2124724339062475
 -0.0007981558612572  0.9457107060955212  0.1432285377612826
 -0.0014813604817387  0.0999284985715652  0.0017929496050477
  0.0000528309004499  0.9013615859040205  0.0015192680390541
  0.0004552336349027  0.0006073658399597  0.9024420751619804
  0.9979746495343939  0.0006888150021720  0.1009663080619606
  0.1305179758888864 -0.0029951963612557 -0.0066638979481615
 -0.0020532361852431  0.9322615295758466  0.2547973579494056
  0.0037508058619142  0.0692129673695381  0.7487437049341921
 -0.0000269682940282  0.9325703557818410  0.7484253650191329
  0.0002896665647189  0.2538188636896264  0.9335468911039289
  0.9946134953123387  0.2537327933477216  0.0701603542750940
 -0.0065066553871770  0.0688426696433686  0.2548608944773587
  0.9986846760275606  0.7473789908397855  0.9333594333279648
  0.0012173163708292  0.7475646311674643  0.0700248743184596
  0.0044045366390439  0.1606385989185381  0.8420391037803373
 -0.0070063426711480  0.1605147994461562  0.1616348881511600
 -0.0022757835974034  0.8406448933159456  0.8415295807992961
  0.0016654502381423  0.8407621950883397  0.1615080364818932
  0.1319700190203876  0.9555994601983728  0.9589478885633118
  0.1613713861044569  0.0445429791924435  0.9679996188608353
```

Ti-porphyrin*NHO

```
   1.00000000000000
    20.0000000000000000    0.0000000000000000    0.0000000000000000
     0.0000000000000000   20.0000000000000000    0.0000000000000000
     0.0000000000000000    0.0000000000000000   20.0000000000000000
   Ti   C    N    H    O
     1    20     5    13     1
Selective dynamics
Direct
  0.0368810556515893  0.9998276630571926  0.0019306632531426
  0.9926588045430119  0.2141538229863441  0.0348747239187615
  0.9979620416647075  0.1448898567805481  0.0560801515642519
  0.9976734357916439  0.1227163983421113  0.1225627614530373
  0.9935845726389969  0.7856502079517481  0.9667695700699497
  0.9995077935088996  0.8546932408287169  0.9452432430846772
  0.0002115562595103  0.8771996454619206  0.8789255643815547
  0.9944440455686057  0.7861141504933762  0.0353368288850128
  0.0006118110196227  0.8554438902996281  0.0559482267776220
  0.9941196685461465  0.2141359333387200  0.9663546266130274
  0.0011327360434193  0.8780968312024574  0.1222722394709857
  0.0011003522499358  0.0341879048570771  0.7889842942956699
  0.0003737635166473  0.1448546224403307  0.9455286160707397
  0.0041020218674669  0.0553627896009999  0.8580442280220393
  0.0001049754740770  0.9655393314472571  0.7888596034932081
  0.0026181222892734  0.9440500585936292  0.8578447595645181
  0.0025217345406630  0.1221640711008522  0.8792947397525197
  0.9957993064409636  0.0345603887038777  0.2132172051265532
  0.9995305948347819  0.0560847058497699  0.1443111323389928
  0.9970901445962229  0.9658793113333142  0.2130571509732091
  0.0015100637249013  0.9447792365920484  0.1440456092707991
  0.0024445084087905  0.1036719322193244  0.0008381409199365
  0.0037509745146449  0.8962500406122497  0.0002227843754576
  0.0056791569792308  0.9996172553827344  0.8995838746352808
  0.0033624693700963  0.0006015878722343  0.1027461105132841
  0.1287837769135045  0.9669775109997171  0.0036645633241781
  0.9946924080360736  0.9322201969494757  0.2557533940429291
  0.9991812552442317  0.0678421586464879  0.7462550149942273
  0.9972730230103001  0.9320981590234579  0.7460160818732687
  0.9907649387446166  0.2567388718807010  0.9326499787794255
  0.9879972737268006  0.2567798255645714  0.0683945332696277
  0.9921783182837207  0.0679185266386606  0.2560673448142491
  0.9888250160929509  0.7428240901318469  0.9335178629131371
  0.9905416854213347  0.7437338151121035  0.0692623881215312
  0.0009714313115086  0.1601328116021348  0.8400695395189979
  0.9944576477759034  0.1610208216216439  0.1613566997742133
  0.9973016786948570  0.8392831043107518  0.8397309472865564
  0.9989751063068902  0.8398791199541406  0.1612327213860687
  0.1485097118196847  0.9496393882690071  0.9589220682912389
  0.1226055185737386  0.0382527219468300  0.9979800127861175
```

V-porphyrin*NHO

```
   1.00000000000000
    20.0000000000000000    0.0000000000000000    0.0000000000000000
     0.0000000000000000   20.0000000000000000    0.0000000000000000
     0.0000000000000000    0.0000000000000000   20.0000000000000000
   V    C    N    H    O
     1    20     5    13     1
Selective dynamics
Direct
  0.0292180372026456  0.9991288682455474  0.9998975377210690
  0.9920440593290026  0.2117866569775453  0.0342414445935404
  0.9969813071242516  0.1427741485806985  0.0552467946350440
  0.0000424950365782  0.1217113046397163  0.1219951801173552
  0.9930262794741584  0.7883505635566158  0.9654430169996133
  0.9981010574552401  0.8571839080387309  0.9442470539210310
  0.0005546273295598  0.8786563357693824  0.8775721108161089
  0.9931697452123467  0.7883325519699496  0.0341788231656155
  0.9983090127219965  0.8571552853040543  0.0553942003370783
  0.9922006115859088  0.2118127316251983  0.9656323584967499
  0.0008994155836449  0.8785844162069910  0.1220809894391852
  0.0003759231153569  0.0345966042342501  0.7863626479273518
  0.9972158647431258  0.1428157243660529  0.9445975683278373
  0.0040100739015455  0.0556819037703792  0.8556151588359845
  0.0003316979668973  0.9660639514514212  0.7863268552184091
  0.0039611769343194  0.9448739699111334  0.8555592465525113
  0.0004702591843350  0.1217900794819080  0.8778486502162272
  0.0001476023095569  0.0344430827326027  0.2134206610508976
  0.0037266573278691  0.0556000051613753  0.1441874303505612
  0.0004251766515193  0.9659111974383020  0.2134032878437318
  0.0041428005855713  0.9447896769795037  0.1441539554036975
  0.9988151790510008  0.1009834966661534  0.9999105335824428
  0.0002472664611183  0.8989512366803424  0.9998269633232070
  0.0076978998127526  0.0002895403416419  0.8971866230868522
  0.0076633150796704  0.0002548677978243  0.1025748348003537
  0.1161746030325534  0.9715605928408995  0.9998169625848060
  0.9971377452700999  0.9322377129466785  0.2560348660596021
  0.9969047555719251  0.0682773534920882  0.7437507616285607
  0.9968883296719206  0.9324375440827321  0.7436707090466690
  0.9892943271627468  0.2544626609808160  0.9319407762600890
  0.9889824758556777  0.2544100921427913  0.0679524681042720
  0.9965131475688961  0.0680729722347026  0.2560585023513693
  0.9897900128468379  0.7456269880229873  0.9318765202276632
  0.9900711714645941  0.7455906296814215  0.0677340018844570
  0.9984238889137252  0.1607503084902741  0.8396262621869659
  0.9978122292874454  0.1606429585068804  0.1602363094638465
  0.9979218288051956  0.8399038611029724  0.8391707674717785
  0.9984313933335007  0.8397925119928678  0.1604534447949619
  0.1578523860381727  0.9415470567330559  0.9988549202247163
  0.1258586639967231  0.0380306488215332  0.9996688009477589
```

Co-porphyrin*HNOH

```
   1.00000000000000
    20.0000000000000000    0.0000000000000000    0.0000000000000000
     0.0000000000000000   20.0000000000000000    0.0000000000000000
     0.0000000000000000    0.0000000000000000   20.0000000000000000
   Co   C    N    H    O
     1    20     5    14     1
Selective dynamics
Direct
  0.0086075222689945  0.0008058917064900  0.0012404070085170
  0.9968314819827825  0.2127530970297209  0.0350874279081474
  0.9981470991343808  0.1434798943476744  0.0560875331317192
  0.9979756215012339  0.1230780719979436  0.1225607342917661
  0.0016200496234910  0.7899023320257478  0.9674274595297369
  0.0023881401051268  0.8590812675348279  0.9463208827478958
  0.0027735559404042  0.8794382213412552  0.8798481029575300
  0.0012064818200542  0.7901742542879392  0.0357774374430428
  0.0017021327721454  0.8594946891798249  0.0564242399886352
  0.9980720409841530  0.2125760371203333  0.9667533831419554
  0.0008387620714488  0.8801434359403237  0.1227876090512018
  0.0019082858900883  0.0351665943235932  0.7900755634280128
  0.0000884502455849  0.1431999852718497  0.9461822098936823
  0.0024404877950087  0.0560100548603019  0.8593084289597051
  0.0017632263733285  0.9667677185972696  0.7899636814005048
  0.0023642039473673  0.9458208173759804  0.8591410237324443
  0.0019881818746868  0.1224463549759467  0.8798649803167542
  0.9962004005503501  0.0358522357648295  0.2125458716740433
  0.9983408757002911  0.0566792052599956  0.1433151245193870
  0.9968425627172940  0.9674279061873380  0.2126326018933822
  0.9994763633189276  0.9465145305245345  0.1434748877790991
  0.9995083143653244  0.1010329344680324  0.0012422166528274
  0.0020745733310751  0.9016863723354863  0.0012397272557198
  0.0023479007892192  0.0008731907299856  0.9016280943411962
  0.0002647096810223  0.0015377721096988  0.1010560714988372
  0.1056285617283860  0.0039483934821527  0.9962117000907212
  0.9952559516591797  0.9334038274239587  0.2550822900801254
  0.0012730982063954  0.0692748324954256  0.7476709159624240
  0.0010331731896516  0.9327430497695304  0.7475004021562144
  0.9974442081495765  0.2548983800493271  0.9325432324955444
  0.9949755607243336  0.2552464600575417  0.0690482818858156
  0.9939921878927039  0.0699281165738065  0.2549328367001829
  0.0011050984674351  0.7473647842680596  0.9334913865820795
  0.0002514886780540  0.7478861037474673  0.0700211012628111
  0.0022933321682536  0.1609482536761299  0.8411795792340431
  0.9968095831537447  0.1617374881113512  0.1610670196689886
  0.0029040055098593  0.8407495426817937  0.8413597980041748
  0.0003130974837178  0.8415271255816898  0.1613492064924615
  0.1147986375743606  0.9744093984353809  0.9545847696073061
  0.1385052776455271  0.9933562575674962  0.0846245151611545
  0.1389298129850000  0.9643561207819901  0.0452472640702087
```

Cr-porphyrin*HNOH

```
   1.00000000000000
    20.0000000000000000    0.0000000000000000    0.0000000000000000
     0.0000000000000000   20.0000000000000000    0.0000000000000000
     0.0000000000000000    0.0000000000000000   20.0000000000000000
   Cr   C    N    H    O
     1    20     5    14     1
Selective dynamics
Direct
  0.0144416261038770  0.0012636861382746  0.0015302595195212
  0.9950440463784200  0.2147808642020654  0.0353279661819907
  0.9977145363458190  0.1455476369429833  0.0566573590184075
  0.9974178615813301  0.1239731856254937  0.1232525422200823
 -0.0002740644379003  0.7884621506669874  0.9675435633872143
  0.0013612557222507  0.8576746772084000  0.9461661404966333
  0.0020810276050343  0.8792919021915365  0.8795995732956353
  0.9991563254128416  0.7886990496762173  0.0360945832131952
  0.0004078441114526  0.8580328229124490  0.0571066145276241
  0.9959412511081186  0.2146345444855852  0.9667845526699383
 -0.0004322393003923  0.8798892586469659  0.1236015018810298
  0.0007515623931172  0.0356088616860113  0.7884578515557660
  0.9991590502018518  0.1453259394286897  0.9458219331671071
  0.0019883747514302  0.0568727924090518  0.8576159543838918
  0.0009550301785242  0.9669647279031092  0.7884591020542723
  0.0023861081260673  0.9458056289872545  0.8576380288367168
  0.0006910587147018  0.1234572990000359  0.8793724408830176
  0.9951340366223250  0.0363477005304810  0.2144337512560606
  0.9982364008465296  0.0574595423664114  0.1452688627003333
  0.9955937511296196  0.9676872267030046  0.2145445513205120
  0.9990299235216684  0.9464049263177817  0.1454636299267188
  0.0001091667617444  0.1038866794752861  0.0013469224694660
  0.0016630158039526  0.8994410837308484  0.0015404269076313
  0.0032160908746017  0.0013918663618018  0.8992442305409005
  0.0010085670227472  0.0018870307312455  0.1038393280720191
  0.1128378202427942  0.0011196941814296  0.9957486442495702
  0.9933574185121699  0.9341340322440834  0.2573442272009083
  0.9994072055351516  0.0692167073476712  0.7456672173604322
 -0.0001566178247734  0.9333320824657598  0.7456837797635648
  0.9943367207026461  0.2572888224241119  0.9330103308236392
  0.9925120512840538  0.2575641994651481  0.0688791982587542
  0.9923701648966586  0.0700247576420456  0.2571063754167158
  0.9989649990334439  0.7455988963713558  0.9340164464709573
  0.9978323773073645  0.7460476578812656  0.0698782277862108
 -0.0001844223090646  0.1619806565580815  0.8406698881877116
  0.9953316452226900  0.1626599052977560  0.1617447305002254
  0.0015821059762384  0.8406004930268683  0.8410607483263965
  0.9982972934135639  0.8412652442664919  0.1621948624898131
  0.1261520064057401  0.9778188375607970  0.9516052557389288
  0.1501136124559239  0.9866375163807952  0.0828089196701634
  0.1457485115656497  0.9576404125584034  0.0437694772703121
```

Cu-porphyrin*HNOH

```
   1.00000000000000
    20.0000000000000000    0.0000000000000000    0.0000000000000000
     0.0000000000000000   20.0000000000000000    0.0000000000000000
     0.0000000000000000    0.0000000000000000   20.0000000000000000
   Cu   C    N    H    O
     1    20     5    14     1
Selective dynamics
Direct
  0.9965868499515110  0.0001118256095564  0.9980507314643708
  0.9966107584614087  0.2126753570526674  0.0329113768789509
  0.9969156813246584  0.1432470281914056  0.0539341283574240
  0.9979581440034342  0.1219151040649258  0.1205171751079858
  0.9953846783365858  0.7875164956626909  0.9642809076940108
  0.9950849804017747  0.8569409983207142  0.9432862745689692
  0.9939582491693483  0.8783755313574044  0.8767549788546336
  0.9960900494959797  0.7874463199740112  0.0327663989625641
  0.9964259544891343  0.8568346416261696  0.0538643230816521
  0.9952993324158859  0.2126799828104691  0.9644363138761911
  0.9970897129798415  0.8780815824582457  0.1204845545177874
  0.9922072241468640  0.0344159519921380  0.7858116464836137
  0.9946795608708831  0.1432794863159860  0.9433823460402934
  0.9927987921187891  0.0554221768305749  0.8552468571056465
  0.9924608504453772  0.9659155680887678  0.7857911361048553
  0.9931797832165127  0.9448712390270328  0.8552112872610662
  0.9932893296496542  0.1219103978261913  0.8768384992621480
  0.9964576724927620  0.0343165975469327  0.2113741633521992
  0.9984909154529851  0.0554573664067245  0.1421901079228302
  0.9960849032599571  0.9657160380186526  0.2113954421710764
  0.9979346739631112  0.9445265239456631  0.1422060418485780
  0.9956405132404156  0.1017785281376145  0.9986321807963229
  0.9957399092431373  0.8983860258715900  0.9985962918290082
  0.9932729684378958  0.0001345196543802  0.8966385138557484
  0.0002599521714031  0.9999869550903349  0.1005225560743452
  0.1692297621802778  0.9996013365939006  0.0098897760615600
  0.9944713162194619  0.9320415697719808  0.2541349737768305
  0.9919348221223492  0.0682132378699446  0.7431371774030283
  0.9924246294073764  0.9321433463350280  0.7431044638931916
  0.9947681651259208  0.2553655888083498  0.9306715359407043
  0.9974042283538471  0.2553395827538104  0.0667070780180099
  0.9951909330680294  0.0680083704598600  0.2541088465280197
  0.9949811594796529  0.7448689256022194  0.9304602594280634
  0.9964337150861140  0.7447461053011374  0.0665118885621948
  0.9927917284861327  0.1605991575470097  0.8383171967691148
  0.9977131133074716  0.1606210410548332  0.1590264142282560
  0.9937195820644008  0.8397071799358378  0.8381986500609541
  0.9964490991454837  0.8393389538750425  0.1589586170912055
  0.2207225182337960  0.9969562374244153  0.0027185751349427
  0.1174271334556422  0.9932259288736780  0.0880553153118456
  0.1657211545246740  0.9910021959121472  0.0787749983197900
```

Fe-porphyrin*HNOH

```
   1.00000000000000
    20.0000000000000000    0.0000000000000000    0.0000000000000000
     0.0000000000000000   20.0000000000000000    0.0000000000000000
     0.0000000000000000    0.0000000000000000   20.0000000000000000
   Fe   C    N    H    O
```

```
   1   20    5   14    1
Selective dynamics
Direct
  0.0129679398115995  0.0011078122903170  0.0011355372656176
  0.9938908599036510  0.2132343656783846  0.0350884190206885
  0.9971191913963925  0.1441061332392387  0.0560185404495117
  0.9989690709312998  0.1233992481107068  0.1226029957994629
  0.9994922108779897  0.7904421170028967  0.9677803867017404
  0.0014891438498746  0.8594479379161801  0.9466763788763436
  0.0040621895243045  0.8800874129076499  0.8801066893720915
  0.9985735194525915  0.7905846754360217  0.0363108722946702
 -0.0000578199354079  0.8597095206840741  0.0571054637889956
  0.9942687246874611  0.2132236788993496  0.9665740093751209
  0.0004474496535592  0.8805002897126882  0.1236787376113877
  0.0031180568107986  0.0355427962186216  0.7893593409289574
  0.9976515224373033  0.1440558059756004  0.9458371093436468
  0.0036377204929639  0.0567028333094897  0.8586683028224632
  0.0041852866125936  0.9671812048818770  0.7895038137389622
  0.0053912569949515  0.9463532500608827  0.8588816722164554
  0.0005171014563012  0.1230574472469028  0.8793893244513686
  0.9985633917075689  0.0365848868512879  0.2133706774686462
  0.0012101155256912  0.0571723731429001  0.1439589181350353
  0.9986932364341167  0.9681728848062070  0.2138719575122150
  0.0013905772155709  0.9468327793778287  0.1446983861361915
  0.9985356177048081  0.1019211657606439  0.0009455627100734
  0.0008263412389148  0.9018317325136104  0.0017658644810930
  0.0054999662822208  0.0016218420284036  0.9011043079492915
  0.0037568273736026  0.0017413551335670  0.1020385389406047
  0.1043230238411115  0.9993061138850686  0.9969184332740322
  0.9962738175684672  0.9345125509855307  0.2565753383119897
  0.0011897665041213  0.0693831684040915  0.7467760324625703
  0.0033566628544186  0.9331237095144120  0.7470581743713609
  0.9924546994100799  0.2557160331654389  0.9326071738536114
  0.9917709825387357  0.2557876409147171  0.0689741843270953
  0.9960553021183010  0.0707915272673780  0.2556449521369706
  0.9990577188766030  0.7478007967215454  0.9339620045154502
  0.9971488306164429  0.7481185038477187  0.0703123041971936
  0.9994360950264769  0.1615714052543631  0.8407066758942824
  0.9972860794677557  0.1621756191277509  0.1610112063511626
  0.0040206464888184  0.8412529448858140  0.8417368867446322
 -0.0007880624171608  0.8417529293922693  0.1621217270385530
  0.1225172602084462  0.9844903698532361  0.9509603945797460
  0.1300135630467497  0.9747615136133969  0.0852015951295608
  0.1429686154098652  0.9585606239819571  0.0408611094211573
```

Mn-porphyrin*HNOH

```
   1.00000000000000
    20.0000000000000000    0.0000000000000000    0.0000000000000000
     0.0000000000000000   20.0000000000000000    0.0000000000000000
     0.0000000000000000    0.0000000000000000   20.0000000000000000
   Mn   C    N    H    O
   1   20    5   14    1
Selective dynamics
Direct
  0.0168824732338507  0.9995870518063670  0.0001410697961413
  0.9918414972743009  0.2101618278731874  0.0354038507572412
  0.9962324517469834  0.1413808999274937  0.0564707448309174
  0.9994200677294540  0.1208028838882174  0.1231949476859052
  0.9923557211569020  0.7890515973290535  0.9667397859098832
  0.9972972064526263  0.8578401308544187  0.9458025368301802
  0.0021426242852019  0.8784176278101067  0.8791314494353544
  0.9914631884251583  0.7890386783985422  0.0354140705875662
  0.9959882728374329  0.8578158208424608  0.0564715473107707
  0.9927670544669592  0.2101387800911671  0.9667294043488110
  0.9992406784761237  0.8783943603176926  0.1231915419240247
  0.0070683200949850  0.0337385508086128  0.7880792993174919
  0.9975960242486434  0.1413418917990628  0.9457976069404969
  0.0071869767571235  0.0548165922040950  0.8575716436817913
  0.0069863445656444  0.9654129188721919  0.7880803704867131
  0.0070534368451813  0.9443388962818853  0.8575727684979303
  0.0024212131531911  0.1207491902375256  0.8791296681887828
  0.0013965294792430  0.0337841380157781  0.2141935972781794
  0.0038487396075761  0.0548415491880499  0.1448244624143949
  0.0013532663897222  0.9654028119157471  0.2141895625206169
  0.0037780500472011  0.9443475173483141  0.1448200690146558
  0.9982414438317128  0.0991258321599944  0.0011276445435976
  0.9980446796347376  0.9000611990597142  0.0011251383863933
  0.0086263749854041  0.9995750149708457  0.8998675734944634
  0.0074008271265393  0.9995923933981362  0.1024695827623024
  0.1038864332148452  0.9995370292282165  0.9962563620626352
  0.9983876747691177  0.9315562231728454  0.2567148708267343
  0.0058270025479040  0.0675979933179886  0.7454825644740476
  0.0056609120064799  0.9315503308674289  0.7454884167860791
  0.9906823191930144  0.2527249707029012  0.9329021979131473
  0.9888795797480587  0.2527728035495009  0.0691337021989532
  0.9984731690535711  0.0676276901802523  0.2567234359648568
  0.9901788496607536  0.7464630935305409  0.9329199361619813
  0.9884484716352320  0.7464352690410980  0.0691492551053853
  0.0011950122064481  0.1597289909097737  0.8409121877053498
  0.9970488034654537  0.1597503314651754  0.1613967687548189
  0.0008259818083367  0.8394360266611240  0.8409189911872343
  0.9968196284546733  0.8394510041835468  0.1613953528439883
  0.1324914237390431  0.9994921159417185  0.9535482733527121
  0.1239126445726763  0.9999142393468297  0.0888748703015971
  0.1519331310724499  0.9999247325024029  0.0485428774158847
```

Ni-porphyrin*HNOH

```
   1.00000000000000
    20.0000000000000000    0.0000000000000000    0.0000000000000000
     0.0000000000000000   20.0000000000000000    0.0000000000000000
     0.0000000000000000    0.0000000000000000   20.0000000000000000
   Ni   C    N    H    O
   1   20    5   14    1
Selective dynamics
Direct
  0.9929520042756165  0.0000949958877309  0.0000798216215769
  0.9924788664565707  0.2108183864831060  0.0342097971219287
  0.9928174415832129  0.1416450355048617  0.0550594842980302
  0.9933365475809813  0.1215206768150784  0.1214502030749508
  0.9930898458329024  0.7893754811494683  0.9660723826043235
  0.9930115276400527  0.8585508662173479  0.9452336380670779
  0.9929385190009201  0.8787095087123649  0.8788635854361666
  0.9932394973282984  0.7893818074965951  0.0343973795204967
  0.9932747046968990  0.8585694982296267  0.0552065815502773
  0.9922503796329747  0.2107679380360040  0.9658892845078750
  0.9937388147739200  0.8787508558993093  0.1215790153636718
  0.9924336771754591  0.0341542425758585  0.7894667888725928
  0.9924343601572282  0.1415721240176636  0.9451000773782048
  0.9925107736977659  0.0549857617236597  0.8586527948779737
  0.9926078260718443  0.9658483595155204  0.7894923596575296
  0.9927847473680289  0.9450767244871171  0.8587076692755073
  0.9923477469603054  0.1213620231040891  0.8787510382596185
  0.9939924876622642  0.0343516849540666  0.2108213424946452
  0.9938633555041314  0.0551697913601404  0.1416779203828212
  0.9940918235022511  0.9660055116924879  0.2108579869136830
  0.9940168285670445  0.9451132794918162  0.1417453671389177
  0.9927054753626596  0.0991230505234570  0.0000958591714269
  0.9930475679986074  0.9010555249115818  0.0002026299666930
  0.9926460477436699  0.0000550219938708  0.9011440984522812
  0.9940766199088632  0.0001186011555643  0.0991481709480899
  0.2062862391353871  0.0006961605554646  0.9998038866175154
  0.9941448072907750  0.9318922401049403  0.2532470003718167
  0.9922812538954124  0.0683129590384988  0.7471265388444255
  0.9925866819443628  0.9316381770927855  0.7471825224656887
  0.9920407589925597  0.2530955107178138  0.9317041338984595
  0.9924610750991624  0.2531793113658881  0.0683485256973024
  0.9939693139448960  0.0685320244655466  0.2531687805048973
  0.9931014763994914  0.7470173585706588  0.9319211928198651
  0.9933739032876437  0.7470438228322033  0.0685675803233769
  0.9922487937869787  0.1598956471823976  0.8401486974948944
  0.9933673728598319  0.1601189551790361  0.1599980833440361
  0.9931261690050925  0.8401435738461964  0.8402834999263815
  0.9940171399203555  0.8401881761520542  0.1601512280206979
  0.2552958870249011  0.9955129213391274  0.0166512779164637
  0.1245008788228932  0.9942699710322169  0.0464137455557674
  0.1717952621077714  0.9900074385868100  0.0592780292420606
```

Ti-porphyrin*HNOH

```
   1.00000000000000
    20.0000000000000000    0.0000000000000000    0.0000000000000000
     0.0000000000000000   20.0000000000000000    0.0000000000000000
     0.0000000000000000    0.0000000000000000   20.0000000000000000
   Ti   C    N    H    O
   1   20    5   14    1
Selective dynamics
Direct
  0.0247610405665462  0.9995703609565917  0.0006787362117612
  0.9946856229080553  0.2127574714726119  0.0350459478420737
  0.9980967212057978  0.1440306926253237  0.0563534542025005
  0.9984170810762264  0.1217411696585892  0.1232642901044056
  0.9944103626291526  0.7864409460648631  0.9660777324907753
  0.9985331833409351  0.8551333158117921  0.9447801531491098
  0.0003047937321104  0.8774606816893902  0.8779031991046288
  0.9938990894861065  0.7864703688670645  0.0350610310295481
  0.9976327846712770  0.8551839040673204  0.0563559409191591
  0.9951750636458461  0.2127699256946457  0.9660613105162807
  0.9980443593797275  0.8774747718129291  0.1232634150513786
  0.9989484058451844  0.0338520122791713  0.7865747050709866
  0.9989507664816707  0.1440566998982801  0.9447744427702401
  0.0034271558809334  0.0555606468045705  0.8556678044882133
  0.9988986267157751  0.9653109715782822  0.7865721312143915
  0.0033271240522709  0.9435925533638636  0.8556661698451296
  0.0005874178287911  0.1217014283974609  0.8779020179947759
  0.9942476388276915  0.0339071459230619  0.2145665533848823
  0.0007387123538246  0.0556546363042410  0.1457067817253297
  0.9941545034880669  0.9653174221571149  0.2145650626248442
  0.0005836825602423  0.9435531558659787  0.1457065018677157
  0.0002194385732465  0.1026295440555915  0.0005362366699490
 -0.0000357026551278  0.8965651295246470  0.0005314759113461
  0.0079909807825362  0.9995695069580832  0.8971156796010602
  0.0067854131943755  0.9995950679382296  0.1043474284102223
  0.1205247532713432  0.9995264381310266  0.0000025921441416
  0.9888255244456591  0.9319039365348880  0.2571911205202779
  0.9950165005254326  0.0672956150119289  0.7438104303696356
  0.9949266305433486  0.9318737216116836  0.7438063751949326
  0.9929675076273594  0.2556542604181627  0.9326178236204118
  0.9920562141692818  0.2556252548047661  0.0684780454008464
  0.9890038221185695  0.0673332208123245  0.2571933237800341
  0.9920011761595189  0.7435630515851842  0.9326387870122108
  0.9910540906979169  0.7436239135328274  0.0685035185398838
  0.9977999899374383  0.1603231650533803  0.8393741579258515
  0.9947850161349090  0.1604210528998936  0.1616607227326690
  0.9974459082285223  0.8388398835710589  0.8393810324001445
  0.9942835949530673  0.8388060140069304  0.1616592258815293
  0.1505528688450095  0.9994067141759995  0.9584249633791502
  0.1394474133709306  0.9998241890434019  0.0939791031471467
  0.1678092224003838  0.9998010390368328  0.0541005757504358
```

V-porphyrin*HNOH

```
   1.00000000000000
    20.0000000000000000    0.0000000000000000    0.0000000000000000
     0.0000000000000000   20.0000000000000000    0.0000000000000000
     0.0000000000000000    0.0000000000000000   20.0000000000000000
   V    C    N    H    O
   1   20    5   14    1
Selective dynamics
Direct
  0.0231032816580832  0.9995734862162609  0.0005145619860968
  0.9938511439985346  0.2120461344958237  0.0351759847601291
  0.9977363486055687  0.1430664795930621  0.0564489971229314
  0.9989279826853682  0.1214646182797001  0.1232568830287979
  0.9940000502142774  0.7871999933254025  0.9665372893518445
  0.9983793778131015  0.8561805549890180  0.9453790101104492
  0.0008058205775716  0.8777699707325426  0.8785747530510610
  0.9934007891749048  0.7871722930895877  0.0352684810790014
  0.9974604110798039  0.8561499282230818  0.0565095800816441
  0.9944783992157854  0.2119852028675391  0.9664448613282887
  0.9987016995973951  0.8777819026782693  0.1233068102197687
  0.0005442402806948  0.0338378634415787  0.7868901342596283
  0.9986991902110065  0.1429832472367213  0.9453196645541500
  0.0042428572434116  0.0551485456152560  0.8561425349970652
  0.0004452970322702  0.9652589577743722  0.7869020462416252
  0.0040901614819888  0.9439618966121028  0.8561623856919887
  0.0011146701093731  0.1213575246615375  0.8785271880128905
  0.9961567466328303  0.0339515119280353  0.2147487106037301
  0.0017550530058554  0.0552517371349975  0.1456805894010736
  0.9960969978456240  0.9653378539661776  0.2147613525130396
  0.0016582804455493  0.9439993102953887  0.1457022721280404
  0.0000496800837833  0.1014407490661381  0.0009228088677700
  0.9998589535090276  0.8977427346586591  0.0009629986377673
  0.0079735210332198  0.9995560928910630  0.8976523001518920
  0.0070245835300032  0.9996122330015554  0.1041962817382604
  0.1158463520301042  0.9995472472012317  0.9979449414228325
  0.9912652217141112  0.9318164531670680  0.2573804128910103
  0.9971472288530056  0.0673789525223630  0.7441436239326711
  0.9969476840204247  0.9317135133148891  0.7441662560080783
  0.9922881122900477  0.2547494148169320  0.9328375889743370
  0.9910631204444542  0.2548536885511914  0.0686822660540089
  0.9913828096645522  0.0674981000028543  0.2573539025021355
  0.9916898945187426  0.7444282426523623  0.9329487911727328
  0.9905276716217870  0.7443864774422800  0.0687952774318121
  0.9987256135563772  0.1602086225670460  0.8401990165480263
  0.9956252256528402  0.1602881470684858  0.1615476201245231
  0.9982977606788477  0.8389088128963746  0.8402642398831208
  0.9953116004390735  0.8389820506837889  0.1616148556531607
  0.1449445201360947  0.9993997664332043  0.9557054949926671
  0.1358762876959061  0.9999039365192565  0.0912836058611780
  0.1637898596185667  0.9998267513868246  0.0510436266287609
```

Co-porphyrin*ONH2

```
   1.00000000000000
    20.0000000000000000    0.0000000000000000    0.0000000000000000
     0.0000000000000000   20.0000000000000000    0.0000000000000000
     0.0000000000000000    0.0000000000000000   20.0000000000000000
   Co   C    N    H    O
   1   20    5   14    1
Selective dynamics
Direct
  0.0117203623234231  0.0021481564215403  0.9970594001942115
  0.9971090493625090  0.2129746464399180  0.0318368957247463
  0.0010379319616533  0.1437361935019558  0.0525755635339971
  0.0044600358751282  0.1232656046350633  0.1189252808862151
  0.0070637868442964  0.7904304653038990  0.9629809657947949
  0.0060813565272653  0.8596648409217627  0.9421265126900040
  0.0036846789388335  0.8803879806961586  0.8757512553174772
  0.0091561863707971  0.7904198962203418  0.0313775516343492
  0.0095133489937482  0.8596688422291722  0.0522352288792608
  0.9951754653809196  0.2129543935612527  0.9635195262100903
  0.0116037342342349  0.8803718894150659  0.1186654439794777
  0.9960826622129275  0.0360077319626167  0.7862823700586101
  0.9978281048689018  0.1437136899918559  0.9426350023052329
  0.9983729994781126  0.0567703344045156  0.8555687137492840
  0.9977325899065221  0.9676832614234786  0.7861417634102980
  0.0011429559462142  0.9468015735549342  0.8553608909374909
  0.9971904860890547  0.1231465506382677  0.8762230941124804
  0.0089343563911784  0.0360070330294792  0.2087489643204181
  0.0074380775945640  0.0568985643760602  0.1395296338798143
  0.0108176100398756  0.9676153979531849  0.2087358072871060
  0.0104941488899398  0.9467781498270683  0.1394647289808714
  0.0007666843314765  0.1014589503989583  0.9975445401725012
  0.0074529485240060  0.9020272273488742  0.9971712245601517
  0.0010551523040284  0.0017767916449549  0.8977576118776626
  0.0083817430043405  0.0018821689031900  0.0971397107413212
  0.1490109521459985  0.9694825106222599  0.0207801466630089
  0.0118032780428298  0.9335966449404819  0.2512048629056581
  0.9932301944000743  0.0701071390350688  0.7439737297878026
  0.9965450640584221  0.9336630621346016  0.7436859179785857
  0.9920192403669379  0.2553095377309094  0.9295005960053844
  0.9958817431932556  0.2553586246463162  0.0659442951652841
  0.0080841097524338  0.0700080569753883  0.2512265758012547
  0.0058044926474936  0.7479627959093714  0.9289749348247393
  0.0099678165094515  0.7479363085028636  0.0653803008930096
  0.9949601256238390  0.1617256648721353  0.8376753612928853
  0.0040821244948374  0.1618683682251575  0.1575054605082346
  0.0030661743298999  0.8418626213152419  0.8370939770440982
  0.0130448200539577  0.8417418518951122  0.1572219290597499
  0.1473410517849428  0.9213824407471055  0.0026331464020514
  0.1465895933710562  0.9712946268621346  0.0721291863373903
  0.1077572628305977  0.0119804107822655  0.9897618980929992
```

Cr-porphyrin*ONH2

```
   1.00000000000000
    20.0000000000000000    0.0000000000000000    0.0000000000000000
     0.0000000000000000   20.0000000000000000    0.0000000000000000
     0.0000000000000000    0.0000000000000000   20.0000000000000000
   Cr   C    N    H    O
   1   20    5   14    1
Selective dynamics
Direct
  0.0154618279198836  0.0029410796391796  0.0023665019217583
  0.9950677372406722  0.2154336451773576  0.0376364241744761
  0.9978568847999353  0.1461555812082931  0.0586080905633667
  0.9976780627750739  0.1241105535949566  0.1250598268206521
  0.9977934393860589  0.7896917821963400  0.9673578137407535
  0.0002553258315942  0.8590078727755786  0.9464536398224608
  0.0010342041596450  0.8809665520847255  0.8799797204863288
  0.9973903473998936  0.7894806390597087  0.0359442423956291
  0.9995737065262905  0.8586538491225492  0.0573402222289625
  0.9957877822387506  0.2156352029490599  0.9690261100828556
  0.9994170064823715  0.8801671853517923  0.1239545639421566
 -0.0001121645053895  0.0376966004334929  0.7896236585058198
  0.9989967989552384  0.1465187730923790  0.9476423172473198
  0.0008224829863645  0.0585395510077374  0.8589630906161765
  0.0001508138627229  0.9690800372833558  0.7892624557029979
  0.0012412808695724  0.9475817051187158  0.8584259508851334
  0.0001263111553670  0.1249493706724149  0.8810646287071240
  0.9959969304121811  0.0360055974399593  0.2157014646978840
  0.9982430190509002  0.0574798347677469  0.1465516750188871
  0.9965486808206588  0.9674178532303419  0.2153733664024588
  0.9990920323448412  0.9465981168754790  0.1460378527725836
  0.0001963791774326  0.1048284928147716  0.0029925880049428
  0.0011708883233768  0.9003906124407126  0.0020485674965529
  0.0017318338965485  0.0028843437196870  0.9002319680777923
  0.0001310509403747  0.0022350593987800  0.1047521835424981
  0.1439308675693826  0.0424284817679808  0.9627845028883116
  0.9949930293993633  0.9335443565688030  0.2579558877120054
  0.9988502269935159  0.0715634180630627  0.7470157217728642
  0.9992766353755087  0.9356343644017366  0.7463246111313885
  0.9939354214739579  0.2584839589924455  0.9354999515191339
  0.9924280043774789  0.2580443230333410  0.0714072842506624
  0.9938354233902313  0.0694490127783007  0.2585912796594215
  0.9961618320436298  0.7470676154254510  0.9335449286140259
  0.9954192680763692  0.7466363776155265  0.0694633359144726
  0.9994441514791679  0.1637207142927422  0.8425901503296102
  0.9959711619953656  0.1625538332471656  0.1638288126702066
  0.0004875320271290  0.8424615832767195  0.8412426503992738
  0.9984265350304652  0.8413845369188668  0.1624093287877460
  0.1745015355574019  0.0715223495197495  0.9923315510981536
  0.1744756609770864  0.0118377931494223  0.9347609397076967
  0.1094945511835266  0.9991883894935364  0.0076501396874663
```

Cu-porphyrin*ONH2

```
   1.00000000000000
    20.0000000000000000    0.0000000000000000    0.0000000000000000
     0.0000000000000000   20.0000000000000000    0.0000000000000000
     0.0000000000000000    0.0000000000000000   20.0000000000000000
   Cu   C    N    H    O
   1   20    5   14    1
Selective dynamics
Direct
```

```
-0.0029229660031183 -0.0004766577634983  0.0000101637091318
 0.9979218665226793  0.2126519222123306  0.0342498454274232
-0.0038664188986339  0.1433088126351043  0.0552676001876162
-0.0028871646180410  0.1217113086083856  0.1217113467959631
 0.9985014861463670  0.7873378916692748  0.9657635904872177
-0.0029688818499206  0.8567125434398056  0.9447648891686886
-0.0022372828165070  0.8782579543391784  0.8783203407833865
 0.9985043671835898  0.7873332803209702  0.0342076496896446
-0.0029649013929925  0.8567065326524416  0.0552134777090702
 0.9979045327611100  0.2126561675073044  0.9657480109838983
-0.0022237374587506  0.8782491431672153  0.1216591814917280
-0.0010353305567128  0.0341883458425408  0.7874769443926335
-0.0038974633493718  0.1433157865048857  0.9447223964633642
 0.9969438312112660  0.0552344564770924  0.8568611678577763
 0.9990138951511703  0.9657485820726198  0.7875140072871060
-0.0028558521481732  0.9447647297488970  0.8569369866361495
-0.0029173754076131  0.1217298230257616  0.8782757164692984
-0.0010205702227935  0.0341742565632246  0.2125020717482578
 0.9969571123180007  0.0552195615036313  0.1431151738478506
-0.0009703439334624  0.9657395905956039  0.2124654222040653
-0.0028454285266933  0.9447560639724505  0.1430405933084165
-0.0053404769255750  0.1018058282200084 -0.0000063705505172
 0.9959533107702769  0.8981169242683262 -0.0000067756031381
 0.9959625813432179 -0.0000176599766446  0.8983072643028055
 0.9959628128844739 -0.0000302428750562  0.1016722780277799
 0.1618515073618043  0.0569735147282888  0.9974195914966685
 0.0012474904803721  0.9319491682903633  0.2550123759023926
 0.0007949723095762  0.0679173938475168  0.7447992117330632
 0.0012399118831193  0.9319572516581979  0.7449690711828365
 0.9992497664516703  0.2552957901806446  0.9320088290018905
 0.9992896168997177  0.2552778899038103  0.0679965263714463
 0.0008114641806465  0.0679061225483299  0.2551743534716397
 0.0001892649358536  0.7447138894495188  0.9320203110903046
 0.0001952009090794  0.7447144505488191  0.0679481906618948
-0.0013343701301407  0.1602757761152452  0.8396261457533410
-0.0012692497177250  0.1602630578710380  0.1603499130310754
-0.0000554947104754  0.8397219927441163  0.8396866494438696
-0.0000315902397418  0.8397112359918244  0.1602893468074451
 0.1600995424291412  0.0854530598973294  0.0400977700731914
 0.1594856582639536  0.0821116004772830  0.9527305026038161
 0.1388492065093319 -0.0034661389841703  0.9998782385495110
```

Fe-porphyrin*ONH2

```
   1.00000000000000
    20.0000000000000000    0.0000000000000000    0.0000000000000000
     0.0000000000000000   20.0000000000000000    0.0000000000000000
     0.0000000000000000    0.0000000000000000   20.0000000000000000
   Fe   C    N    H    O
     1   20     5    14     1
Selective dynamics
Direct
  0.0120845645424124  0.0029566711353420  0.0021244216823585
  0.9978931119550953  0.2141088024747418  0.0373270396119912
  0.9997143240654054  0.1447796521198700  0.0578399002042366
  0.9995096181624173  0.1234880084992634  0.1242010075452427
  0.0003316771266256  0.7910896467329310  0.9671964663260644
  0.0020755148813590  0.8604519262998562  0.9466966472946881
  0.0034624138842705  0.8817414323393445  0.8803811211548641
  0.9984877792551846  0.7906539442295876  0.0356306378301610
  0.9991036303643628  0.8598077571872184  0.0568947296394189
  0.9975289290796843  0.2145943139250929  0.9688437636652472
  0.9976985275238839  0.8803709001334901  0.1234271324243904
 -0.0001738178760205  0.0378615867472413  0.7906039369037795
  0.9991875503415916  0.1455109422513705  0.9475337570911960
  0.0002854920619305  0.0585757963478028  0.8598820011518675
  0.0013190853593360  0.9693916075422830  0.7904985660800747
  0.0026057386556751  0.9482849273399313  0.8596660534311416
 -0.0006251239860265  0.1249217802828349  0.8810160684775136
  0.9955696858410069  0.0358543485418096  0.2140071110507495
  0.9985835871097972  0.0569729961287840  0.1448804806053748
  0.9948302438888853  0.9674028711356998  0.2138325929671923
  0.9973905414625007  0.9467412482755088  0.1445405783832045
  0.0003717668404407  0.1029740492122774  0.0024247372454548
  0.0009822870107272  0.9023103532732306  0.0020501460334033
  0.0017712948245574  0.0033409537091995  0.9019539816887447
  0.9992914393005665  0.0019849535627591  0.1025411989142971
  0.1397116080226521  0.0414173327874669  0.9658684926751516
  0.9923414398375231  0.9333366950610733  0.2562007391681697
  0.9981832153184893  0.0718774549704658  0.7481538235559262
  0.0012486950489409  0.9355324598245766  0.7478719662798604
  0.9958744851521942  0.2573370521498123  0.9351947990030631
  0.9966914375738398  0.2564192503092391  0.0715591753063701
  0.9938912873797844  0.0696968235594385  0.2566121773185388
  0.0002366605332726  0.7487455397115880  0.9329916871736345
  0.9964787208811667  0.7479562730919019  0.0693143462265682
 -0.0016828955494382  0.1636768229592975  0.8425681774150462
  0.9986198216457695  0.1619433706230796  0.1629598184304046
  0.0039357561760560  0.8433190100455505  0.8415836682367099
  0.9962895640355252  0.8416123102828401  0.1618584723474412
  0.1689680706405661  0.0707870167465565  0.9963364325371089
  0.1699375031647113  0.0115988317255031  0.9368160291233339
  0.1032792684632480  0.9985432867241416  0.0079161178000188
```

Mn-porphyrin*ONH2

```
   1.00000000000000
    20.0000000000000000    0.0000000000000000    0.0000000000000000
     0.0000000000000000   20.0000000000000000    0.0000000000000000
     0.0000000000000000    0.0000000000000000   20.0000000000000000
   Mn   C    N    H    O
     1   20     5    14     1
Selective dynamics
Direct
  0.0144061203807544  0.0037507742831861  0.0012390449148894
  0.9934565396954657  0.2145838183213211  0.0371909597929724
  0.9967504653910562  0.1455384784039190  0.0581691153666263
  0.9970765068915809  0.1237237623331005  0.1244342507046549
  0.0019586863746496  0.7905292897553506  0.9672387049143164
  0.0026556718981500  0.8598289065668058  0.9465638949085213
  0.0027986679267426  0.8810192772229769  0.8800457406111885
  0.0003785876045706  0.7901384486407046  0.0357371832504996
  0.0005118943682632  0.8592938707255114  0.0570656355199358
  0.9947701699695014  0.2147686171971003  0.9685996823666089
  0.9996704521360439  0.8801106155528636  0.1236614998814841
  0.9993048883426554  0.0374290296891790  0.7900204967751947
  0.9984301657213039  0.1457142647362991  0.9474230003116957
  0.0006554679261017  0.0581711012959228  0.8592845310421453
 -0.0003763339857354  0.9688272544054423  0.7897070044596359
  0.0016019648127893  0.9474171101085742  0.8586717448962231
  0.9993136138254374  0.1245391659311994  0.8809031417176835
  0.9965577727309446  0.0358985836873877  0.2143273094896282
  0.9981541444951187  0.0570750117609450  0.1451928525190281
  0.9965757275525137  0.9673798688815560  0.2142230571889278
  0.9985953157657141  0.9465569023493114  0.1449457664556791
  0.9996305140076753  0.1037146171617119  0.0027533615075408
  0.0015797760875574  0.9016218752766761  0.0020987487054054
  0.0021131677112764  0.0025836591603470  0.9009375727421972
  0.9992359283008786  0.0018367795497399  0.1030272766329729
  0.1416558951590663  0.0431165717400964  0.9664207940057823
  0.9947484868951588  0.9334625258897089  0.2567480953178200
  0.9980722027005098  0.0714151177496445  0.7475286371035993
  0.9987137548634998  0.9352663387548430  0.7468612593130654
  0.9932261030421604  0.2574749973866229  0.9348955615186892
  0.9906172811129901  0.2571370590522316  0.0710134427089498
  0.9948907764451785  0.0696592934481758  0.2569925267493887
  0.0019641795205455  0.7480812621617031  0.9331644126656564
  0.9986332262806209  0.7473462681023774  0.0693256131805904
  0.9974381929390005  0.1632353176984842  0.8424242172059863
  0.9953524795965535  0.1619664346074317  0.1633812979559525
  0.0028748725007885  0.8423128086007254  0.8415236700819858
  0.9995738081656621  0.8413766414587898  0.1621316517906282
  0.1700303806407674  0.0731974614946199  0.9970577686151906
  0.1731904875881025  0.0140117143811095  0.9380258625558846
  0.1064964966183591 -0.0011398955236877  0.0088436125551981
```

Ni-porphyrin*ONH2

```
   1.00000000000000
    20.0000000000000000    0.0000000000000000    0.0000000000000000
     0.0000000000000000   20.0000000000000000    0.0000000000000000
     0.0000000000000000    0.0000000000000000   20.0000000000000000
   Ni   C    N    H    O
     1   20     5    14     1
Selective dynamics
Direct
  0.9950241455793865  0.0004149899095306  0.9997550970838017
  0.9903791802231253  0.2111561123133418  0.0339212927257525
  0.9920548893611146  0.1420089548463882  0.0547481486721015
  0.9926640051736934  0.1218214826711830  0.1211288566765629
  0.9984471230671675  0.7898189177858774  0.9656096135903931
  0.9975648307258076  0.8590115237575121  0.9448012781382434
  0.9975252050415553  0.8791389510351553  0.8784184524466894
  0.9984255259361999  0.7898205743836924  0.0339155642202506
  0.9975310793690253  0.8590105680098850  0.0547212271465752
  0.9903509830641660  0.2111546607795186  0.9655968387599836
  0.9974641358135589  0.8791385205632485  0.1211037078395711
  0.9947181366738317  0.0346218599568282  0.7890438798355468
  0.9920183521203195  0.1420091016481546  0.9447720009591071
  0.9942042906861970  0.0554506523919030  0.8582174076427114
  0.9961858692733244  0.9663091813772211  0.7890655575349641
  0.9965472643676776  0.9455017726665574  0.8582368939971758
  0.9926016315120054  0.1218208211912254  0.8783933719949385
  0.9947368935226993  0.0346247457953471  0.2104787961081810
  0.9942309362456925  0.0554501409532517  0.1413031042361387
  0.9961552673559692  0.9663125421545498  0.2104570240440735
  0.9965032777190066  0.9455018182749679  0.1412855884989615
  0.9930933038480212  0.0994886625868353  0.9997594225567529
  0.9969128850936390  0.9014966994606743  0.9997608326848912
  0.9954643451859602  0.0004790213858557  0.9007495669030973
  0.9954528121269038  0.0004789400467967  0.0987738358583622
  0.1822206109953909  0.0558434473971728  0.9996030791550260
  0.9968443032147284  0.9321019305471422  0.2527596016249167
  0.9939940698002391  0.0687951451175926  0.7467139459766691
  0.9968923694991779  0.9321023976977674  0.7467645422883671
  0.9893308543122095  0.2534695663608721  0.9314145527700515
  0.9893855576865689  0.2534712334780204  0.0681038979558776
  0.9940387458813984  0.0687997140215110  0.2528026953140914
  0.9990004083969019  0.7475295708704104  0.9313882940423955
  0.9989589467944203  0.7475304557638652  0.0681346961104897
  0.9916620277676897  0.1603713455693850  0.8398035000657407
  0.9917536067619358  0.1603737764454925  0.1597177643022116
  0.9981941218875574  0.8405550040000273  0.8398631257483550
  0.9981094126082709  0.8405533124607159  0.1596599562567069
  0.1554804597953553  0.0528122156568019  0.0434102781083515
  0.1558541782991479  0.0519584962213085  0.9556365842167646
  0.2453084572129527  0.0416621724464378  0.0000061259091396
```

Ti-porphyrin*ONH2

```
   1.00000000000000
    20.0000000000000000    0.0000000000000000    0.0000000000000000
     0.0000000000000000   20.0000000000000000    0.0000000000000000
     0.0000000000000000    0.0000000000000000   20.0000000000000000
   Ti   C    N    H    O
     1   20     5    14     1
Selective dynamics
Direct
  0.0344774933613868  0.0014465418870647  0.9998415745721586
  0.9903218225649191  0.2157731936296210  0.0343879206769904
  0.9958573343270236  0.1468759242046195  0.0554089029151762
  0.9970057632308191  0.1253758140077749  0.1224592887503365
  0.9978864859861901  0.7896792638006120  0.9653925040968008
  0.0021972360484915  0.8585252349779133  0.9442991108180320
  0.0041777109590441  0.8814299246500927  0.8777888623788934
  0.9978144929891899  0.7896619083627368  0.0343833353261422
  0.0021046840084543  0.8584871588657119  0.0555208724884182
  0.9903165120583002  0.2157722407024512  0.9655811155480197
  0.0040641699765238  0.8813899190263054  0.1220239534496801
  0.9972159021267843  0.0373850590012589  0.7863971540374597
  0.9958502377234957  0.1468828378482507  0.9445504969643290
  0.0008519127605582  0.0593327463283378  0.8553433537210948
  0.0007001239459732  0.9688852572670726  0.7867816638599120
  0.0057956697318624  0.9479236009708065  0.8560337402789071
  0.9969954133520661  0.1254095040422580  0.8774926340041727
  0.9970928100986390  0.0372836955686559  0.2134429142248662
  0.0008849350701580  0.0592739410820388  0.1445118848725987
  0.0005366954474402  0.9687906010569796  0.2130085336353761
  0.0057812987617377  0.9478786844653290  0.1437581663015021
  0.9988140377087898  0.1052546665420568 -0.0000125267463563
  0.0040662364986788  0.8998555894357619  0.9999274377742072
  0.0076156802045471  0.0041604832867781  0.8971927904330756
  0.0077500562483299  0.0041481217972651  0.1026193165547156
  0.1355543162627791  0.0377713929433769  0.9980999797602009
  0.9982361976189419  0.9348583717390511  0.2555171633576982
  0.9916404684998270  0.0704544421898670  0.7435089932219801
  0.9985697439643588  0.9349941394382840  0.7442323942795892
  0.9859433911439840  0.2583816046657582  0.9319787778431560
  0.9859455945676764  0.2583767036115771  0.0679960068389563
  0.9913753537039591  0.0703266076980836  0.2563312676778065
  0.9956746871458312  0.7468126910781827  0.9319203717420839
  0.9955460727655786  0.7467748103375156  0.0678258973957865
  0.9932045493702157  0.1642013245971252  0.8392285057899392
  0.9932023422331717  0.1641255354888217  0.1607620537750213
  0.0020896242737872  0.8432177651538066  0.8388001144376452
  0.0018782526211959  0.8431939019740593  0.1610247002566983
  0.1623513973560594  0.0519369328058992  0.0395663489458417
  0.1611666035928211  0.0504687604210141  0.9554172280034988
  0.1247311896903715  0.9671941030498321  0.9994551957375927
```

V-porphyrin*ONH2

```
   1.00000000000000
    20.0000000000000000    0.0000000000000000    0.0000000000000000
     0.0000000000000000   20.0000000000000000    0.0000000000000000
     0.0000000000000000    0.0000000000000000   20.0000000000000000
   V    C    N    H    O
     1   20     5    14     1
Selective dynamics
Direct
  0.0264122454191744  0.9997091507621717  0.9948490512440283
  0.9994034535261106  0.2115109210439137  0.0311853319666971
  0.0035220884944795  0.1427939500761510  0.0520452123677896
  0.0076582600628578  0.1211201516089609  0.1186745758893975
  0.9968464330838828  0.7878755215198749  0.9614256041303713
  0.9988207768205957  0.8566455552174727  0.9404034518313040
  0.9970849383623943  0.8782634091162903  0.8735238597066861
 -0.0002581914149668  0.7875821499836043  0.0302992383077581
  0.0035430432662766  0.8562002194029191  0.0515051025835141
  0.9965258417168366  0.2115360201034264  0.9623280336771896
  0.0077039964564458  0.8775945695227788  0.1182623307830695
  0.9912199361688252  0.0343303261646308  0.7829207716047266
  0.9988074636756349  0.1428665915340423  0.9409881815324295
  0.9982589143790147  0.0555146151105775  0.8519435522021411
  0.9912139700252224  0.9658635160454856  0.7827710996414415
  0.9982377454659690  0.9443896974221118  0.8517129280861958
  0.9970725827497330  0.1215476041926445  0.8740299244027825
  0.0096868679587524  0.0335109437299148  0.2093624539152560
  0.0110305572733551  0.0549678074873494  0.1401898206026213
  0.0096451955213328  0.9649938410471268  0.2093043526591715
  0.0109358243906437  0.9436942556563426  0.1400663730751591
  0.0025954226477716  0.1009105168275033  0.9963771742577839
  0.0024402089802689  0.8983562396590091  0.9960123800696025
  0.0039416514632978  0.9998600954401146  0.8933899533413233
  0.0134990073224316  0.9993839867792782  0.0979830929459392
  0.1658030937638543  0.0027988653500001  0.0401004997929605
  0.0076637003604372  0.9313270562014343  0.2520415009240483
  0.9859601198921534  0.0680676291527125  0.7405187424418063
  0.9859471761382831  0.9323224210455449  0.7402195363504052
  0.9932355758973227  0.2543571780970352  0.9288869355501860
  0.9990301620142547  0.2542738865377901  0.0648531136372975
  0.0077719858380928  0.0671080969000974  0.2521518631216322
  0.9937070567728058  0.7451925358838702  0.9277970821566326
 -0.0004721365759931  0.7446413063101012  0.0637504768745673
  0.9930460284544504  0.1604843328631901  0.8359488156450355
  0.0068218236209540  0.1597882685556401  0.1572273715851250
  0.9931191282407493  0.8394813755086094  0.8352849245500438
  0.0068680576702594  0.8387509430305389  0.1566442035388904
  0.2033425696222106  0.9719852840988200  0.0228227025776263
  0.1459431285187782  0.9797022239005669  0.0817361042557851
  0.1158487959550270  0.9985679411103713  0.9885122761736027
```

S7. References

(1) Hautier, G.; Ong, S. P.; Jain, A.; Moore, C. J.; Ceder, G. Accuracy of Density Functional Theory in Predicting Formation Energies of Ternary Oxides from Binary Oxides and Its Implication on Phase Stability. *Phys. Rev. B* **2012**, *85* (15), 155208. https://doi.org/10.1103/PhysRevB.85.155208.

(2) Granda-Marulanda, L. P.; Rendón-Calle, A.; Builes, S.; Illas, F.; Koper, M. T. M.; Calle-Vallejo, F. A Semiempirical Method to Detect and Correct DFT-Based Gas-Phase Errors and Its Application in Electrocatalysis. *ACS Catal.* **2020**, *10* (12), 6900–6907. https://doi.org/10.1021/acscatal.0c01075.
(3) Bartel, C. J.; Weimer, A. W.; Lany, S.; Musgrave, C. B.; Holder, A. M. The Role of Decomposition Reactions in Assessing First-Principles Predictions of Solid Stability. *Npj Comput. Mater.* **2019**, *5* (1), 4. https://doi.org/10.1038/s41524-018-0143-2.
(4) Monkhorst, H. J.; Pack, J. D. Special Points for Brillouin-Zone Integrations. *Phys. Rev. B* **1976**, *13* (12), 5188–5192. https://doi.org/10.1103/PhysRevB.13.5188.
(5) Kresse, G.; Joubert, D. From Ultrasoft Pseudopotentials to the Projector Augmented-Wave Method. *Phys. Rev. B* **1999**, *59* (3), 1758–1775. https://doi.org/10.1103/PhysRevB.59.1758.
(6) Urrego-Ortiz, R.; Builes, S.; Calle-Vallejo, F. Fast Correction of Errors in the DFT-Calculated Energies of Gaseous Nitrogen-Containing Species. *ChemCatChem* **2021**, *13* (10), 2508–2516. https://doi.org/10.1002/cctc.202100125.
(7) Sargeant, E.; Illas, F.; Rodríguez, P.; Calle-Vallejo, F. Importance of the Gas-Phase Error Correction for O2 When Using DFT to Model the Oxygen Reduction and Evolution Reactions. *J. Electroanal. Chem.* **2021**, *896*, 115178. https://doi.org/10.1016/j.jelechem.2021.115178.
(8) Calle-Vallejo, F.; Martínez, J. I.; García-Lastra, J. M.; Mogensen, M.; Rossmeisl, J. Trends in Stability of Perovskite Oxides. *Angew. Chem., Int. Ed.* **2010**, *49* (42), 7699–7701. https://doi.org/10.1002/anie.201002301.
(9) Nørskov, J. K.; Rossmeisl, J.; Logadottir, A.; Lindqvist, L.; Kitchin, J. R.; Bligaard, T.; Jónsson, H. Origin of the Overpotential for Oxygen Reduction at a Fuel-Cell Cathode. *J. Phys. Chem. B* **2004**, *108* (46), 17886–17892. https://doi.org/10.1021/jp047349j.
(10) Linstrom, P. J.; Mallard, W. G. NIST Chemistry WebBook, NIST Standard Reference Database 69, 1997. https://doi.org/10.18434/T4D303.
(11) Lide, D. R. *CRC Handbook of Chemistry and Physics, 90th Edition (CD-ROM Version 2010)*, 90th ed.; CRC Press/Taylor And Francis: Boca Raton, FL, 2010.
(12) Irikura, K. K. Experimental Vibrational Zero-Point Energies: Diatomic Molecules. *J. Phys. Chem. Ref. Data* **2007**, *36* (2), 389–397. https://doi.org/10.1063/1.2436891.
(13) Computational Chemistry Comparison and Benchmark DataBase https://cccbdb.nist.gov/introx.asp (accessed 2022 -01 -18).
(14) Kurth, S.; Perdew, J. P.; Blaha, P. Molecular and Solid-State Tests of Density Functional Approximations: LSD, GGAs, and Meta-GGAs. *Int. J. Quantum Chem.* **1999**, *75* (4-5), 889–909. https://doi.org/10.1002/(SICI)1097-461X(1999)75:4/5<889::AID-QUA54>3.0.CO;2-8.
